# Reliable Identification and Interpretation of Single‐Cell Molecular Heterogeneity and Transcriptional Regulation using Dynamic Ensemble Pruning

**DOI:** 10.1002/advs.202205442

**Published:** 2023-06-08

**Authors:** Yi Fan, Yunhe Wang, Fuzhou Wang, Lei Huang, Yuning Yang, Ka‐Chun Wong, Xiangtao Li

**Affiliations:** ^1^ School of Artificial Intelligence Jilin University Jilin China; ^2^ School of Artificial Intelligence Hebei University of Technology Tianjin China; ^3^ Department of Computer Science City University of Hong Kong Kowloon Hong Kong; ^4^ Donnelly Centre for Cellular and Biomolecular Research University of Toronto Toronto ON Canada

**Keywords:** dynamic ensemble pruning, optimization direction, single‐cell RNA sequencing, unsupervised clustering

## Abstract

Unsupervised clustering is an essential step in identifying cell types from single‐cell RNA sequencing (scRNA‐seq) data. However, a common issue with unsupervised clustering models is that the optimization direction of the objective function and the final generated clustering labels in the absence of supervised information may be inconsistent or even arbitrary. To address this challenge, a dynamic ensemble pruning framework (DEPF) is proposed to identify and interpret single‐cell molecular heterogeneity. In particular, a silhouette coefficient‐based indicator is developed to determine the optimization direction of the bi‐objective function. In addition, a hierarchical autoencoder is employed to project the high‐dimensional data onto multiple low‐dimensional latent space sets, and then a clustering ensemble is produced in the latent space by the basic clustering algorithm. Following that, a bi‐objective fruit fly optimization algorithm is designed to prune dynamically the low‐quality basic clustering in the ensemble. Multiple experiments are conducted on 28 real scRNA‐seq datasets and one large real scRNA‐seq dataset from diverse platforms and species to validate the effectiveness of the DEPF. In addition, biological interpretability and transcriptional and post‐transcriptional regulatory are conducted to explore biological patterns from the cell types identified, which could provide novel insights into characterizing the mechanisms.

## Graphical abstract

A dynamic ensemble pruning framework (DEPF) is proposed to identify and interpret single‐cell molecular heterogeneity. In particular, a silhouette coefficient‐based indicator is developed and evaluated to determine the optimization direction of the bi‐objective function. Following that, a bi‐objective fruit fly optimization algorithm is designed to prune dynamically the low‐quality basic clustering in the ensemble.

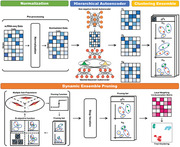

## 1 Introduction

scRNA‐seq technology is a revolutionary technology for analyzing transcriptional profiles at the single‐cell level.^[^ ^1^ ^]^ In recent years, there has been increasing recognition of the important link between cell type heterogeneity analysis and clustering.^[^ ^2^ ^]^ On this basis, clustering has gradually emerged as the most effective method for cell‐type annotation, as it can identify cell types in a bias‐free manner. Earlier studies, including k‐means,^[^ ^3^ ^]^ hierarchical clustering^[^ ^4^ ^]^ and community‐detection‐based algorithms^[^ ^5^ ^]^ have been developed to uncover distinct cell types from single‐cell transcriptome data. However, massive amounts of data and excessive noise render interpretation of scRNA‐seq data by these computational algorithms challenging, greatly restricting the performance of downstream cell type analyses.

Recently, several single‐cell computational models have been proposed to address these challenges; for instance, SINCERA^[^ ^6^ ^]^ uses the z‐score transformed from the scRNA‐seq data to identify the first singleton in the hierarchical clustering structure to target cell clusters. The single‐cell consensus clustering (SC3)^[^ ^7^ ^]^ is suggested to achieve a high level of robustness and accuracy by incorporating several single‐cell clustering algorithms. CIDR^[^ ^8^ ^]^ uses implicit imputation methods to alleviate the effects of dropout in scRNA‐seq data. Seruat^[^ ^9^ ^]^ provides a mechanism for integration of scRNA‐seq data based on common variant sources, allowing the delineation and downstream analysis of cell sub‐populations. SCANPY^[^ ^10^ ^]^ is developed as an extensible toolkit for deconstructing scRNA‐seq data, including clustering, trajectory inference and simulation of gene regulatory networks. However, it is difficult to guarantee all‐round performance of each unsupervised clustering method on all scRNA‐seq data. Indeed, different clustering algorithms have their own advantages and disadvantages, so performance on diverse scRNA‐seq datasets is always inconsistent. In this respect, it is complicated for biologists to determine which single‐cell clustering algorithm is the appropriate choice.

Ensemble clustering is commonly used to yield a better cluster solution from a set of candidate clustering solutions obtained from multiple clustering algorithms, or from multiple implementations of an individual clustering algorithm, and ensemble clustering models have been successfully applied to understand the cellular heterogeneity in scRNA‐seq data. For instance, Yang *et al.* propose an ensemble clustering method called SAFE‐clustering to collect together the clustering results of SC3, t‐SNE+kmeans, CIDR, and Seurat by employing three hypergraph‐based partitioning algorithms.^[^ ^11^ ^]^ Wan *et al.* develop a similarity‐based meta‐clustering called SHARP to handle weighted ensemble clustering for large‐scale scRNA‐seq data.^[^ ^12^ ^]^ Based on the adjusted rand index, SAME‐clustering^[^ ^13^ ^]^ proposes selecting five agents of SC3, t‐SNE+kmeans, CIDR, SIMLR,^[^ ^14^ ^]^ and Seurat as the single‐cell clustering set, and then constructs the final solution using an expectation maximization algorithm. Intuitively, these single‐cell clustering algorithms, including their toolkits, demonstrate generalizability for scRNA‐seq data from different sequencing platforms. However, these ensemble clustering algorithms rely heavily on specific similarity measures for the underlying cluster generation; and, with the accumulation of single‐cell sequencing data, the similarity measures between cells become inactive in the high‐dimensional space,^[^ ^14^, ^15^, ^16^ ^]^ indirectly leading to low‐quality performance.

To settle these issues, a straightforward and reasonable approach would be to transform the high‐dimensional data into a low‐dimensional latent space as a way of capturing the underlying structure of the original data. A recent method called scDHA^[^ ^17^ ^]^ takes advantage of the power of deep learning of potential manifold structures as well as the aggregation ability of ensemble clustering to the several underlying clusters. Indeed, scDHA uses a hierarchical autoencoder to project the data onto a low‐dimensional space, and then employs consensus clustering to determine the outcome by a voting scheme. However, we observe that scDHA has several limitations; for example, the consensus clustering in scDHA may yield unnecessarily large datasets, causing additional time costs and memory consumption. The local diversity of the ensembles cannot be guaranteed, which further affects the performance of the consensus clustering. These problems reduce the reliability of scDHA for identifying and interpreting single‐cell molecular heterogeneity.

Motivated by the above observations, we developed a dynamic ensemble pruning framework called DEPF to identify and interpret the heterogeneity of single‐cell molecules. First, inspired by scDHA, we employ a hierarchical autoencoder as the dimensionality reduction method to project the original data onto several compressed low‐dimensional subspaces. After that, a basic clustering algorithm is applied to yield different clustering results on the learned latent spaces to produce the cluster ensemble. To alleviate the unnecessary costs incurred by the clusters in the ensemble, a bi‐objective fruit fly optimization algorithm is suggested to prune the basic clustering results of the ensemble, enhancing cell type identification and interpretation of single‐cell molecular heterogeneity. In particular, fruit fly optimization algorithm (FOA) is a nature‐inspired biological algorithm that addresses the optimization problem by simulating a swarm of fruit flies foraging for food.^[^ ^18^ ^]^ FOA has several advantages such as the briefness of the calculation process, the convenience of converting the creature concept into computer code, and a straightforward structure. Moreover, FOA is able to converge to the global optimum at a relatively fast rate.^[^ ^19^ ^]^ To guide the optimization, a novel silhouette coefficient indicator is designed to determine the direction of optimization of the bi‐objective function using the mean intra‐cluster distance and the sum of the cell‐to‐center distances of each cell. We conducted multiple experiments on 28 real scRNA‐seq datasets and one large‐scale real scRNA‐seq dataset from diverse yet representative single‐cell sequencing platforms. Results indicated that our proposed DEPF is superior to several state‐of‐the‐art clustering methods. In addition, we carried out gene ontology enrichment analysis, WikiPathways analysis, protein–protein interaction network analysis, transcription factor‐gene interaction analysis, miRNA‐gene interaction analysis, protein‐drug interaction and disease‐gene association analysis to investigate biological insights based on the cell type identification.

## 2 Results and Discussion

### 2.1 Methodology Overview of DEPF

**Figure 1:**
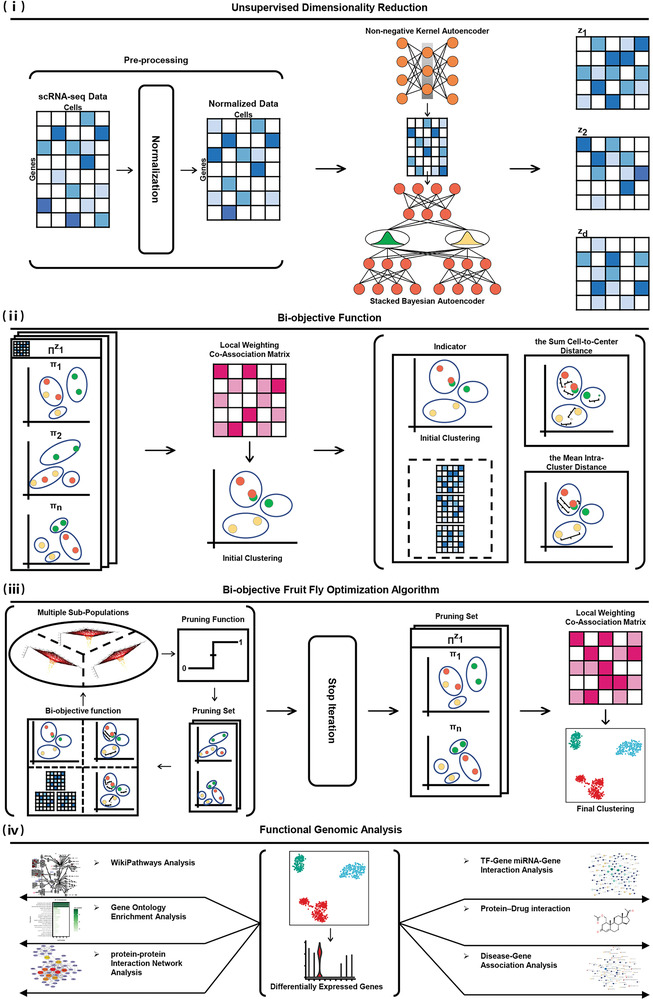
DEPF architecture. i) Multiple low‐dimensional representations of scRNA‐seq data generated by unsupervised methods. ii) The bi‐objective function constructed by an indicator, the mean intra‐cluster distance and the sum of the cell‐to‐center distance for each cell. iii) Ensemble pruning process of a bio‐objective fruit fly optimization algorithm. iv) Diverse functional genomic analyses including gene ontology enrichment analysis, WikiPathways analysis, protein–protein interaction network analysis, transcription factor‐gene interaction analysis, miRNA‐gene interaction analysis, protein‐drug interaction and disease‐gene association analysis.

The DEPF pipeline consists of four components to accomplish the reliable identification and interpretation of single‐cell molecular heterogeneity, as depicted in **Figure** **1**. (i) First, data are processed by removing low quality cells and genes, and then realigning the rest of the data using a logarithmic transformation. Afterward, a hierarchical autoencoder is used to generate multiple potential low‐dimensional space sets to achieve the basic clustering result for subsequent ensemble clustering. (ii) To guide the pruning operation, a new silhouette coefficient indicator is developed to characterize the direction of optimization of the bi‐objective function using the mean intra‐cluster distance and the sum of the cell‐to‐center distance for each cell. (iii) A bi‐objective fruit fly optimization algorithm is designed to prune the ensemble to exploit basic clusterings that are more beneficial to the final result. (iv) Diverse functional genomic analyses, including gene ontology enrichment analysis, WikiPathways analysis, protein–protein interaction network analysis, transcription factor‐gene interaction analysis, miRNA‐gene interaction analysis, protein‐drug interaction, and disease‐gene association analysis were carried out to provide new insights into the interpretation of single‐cell molecular heterogeneity identified in the scRNA‐seq data.

scRNA‐seq data containing *n* cells and *m* genes are first transformed by normalization into a normalized matrix with small standard deviations and no outliers. After that, we take advantage of a hierarchical autoencoder^[^ ^17^ ^]^ to filter out the insignificant genes from the normalized matrix using a non‐negative kernel autoencoder to obtain a subset of features. A stacked Bayesian autoencoder in the hierarchical autoencoder then encodes the gene expression matrix multiple times using a non‐negative kernel autoencoder to provide various low‐dimensional latent spaces that form the ensemble pool *Z* = {*z*_1_, *z*_2_, …, *z*_*d*_}. On this basis, a basic clustering algorithm is employed on each member of the ensemble pool to yield different clustering results for an ensemble clustering set $\Pi =\{\Pi^{z_{1}},\Pi^{z_{2}},\text{…},\Pi^{z_{d}}\}$, where $\Pi^{z_{1}}=\left\{\pi_{1},\pi_{2},\text{…}\pi_{n}\right\}$ indicates that there are *n* basic clustering π in the subspace *z*_1_. Finally, a consensus clustering algorithm is applied to calculate the initial clustering by converting the Π into a local weighting co‐association matrix. Accordingly, a silhouette coefficient is devised as an indicator to evaluate the cells in *Z* to provide an optimization direction to the bi‐objective fruit fly optimization algorithm. Subsequently, the bi‐objective function is constructed using the mean intra‐cluster distance, the sum of cell‐to‐center distance and the optimization direction. Intuitively, the population in our proposed model consists of *FD* fruit flies, where *FD* denotes the number of fruit flies in the whole population. All *FD* fruit flies start from an initial location representing the full ensemble and the initial solutions of the bi‐objective function are calculated to measure the performance of the different individuals. Then, *G* fruit flies form a subswarm while *D* subswarms are assigned to *D* sub‐spaces separately. To find the best basic clustering subsets from the ensemble pool, each fruit fly first uses an olfactory‐based equilibrium search to explore the search space, and then yields a new location. After that, the information of the current location is converted into a smell. Since the smell is a continuous value, a pruning code is designed to transform the smell into a binary representation to indicate the partial ensemble. Then, smells in each subswarm are evaluated in the collaborative vision‐based search. The candidates are calculated by substituting the evaluation clusters and the scRNA‐seq data of the corresponding subspaces into the bi‐objective function. Thereafter, a new location is selected in each subspace to replace the initial location of the subgroup, and if the candidate solution is better than the original solution, the candidate solution is used to replace the original solution. The optimal individuals in each subpopulation will then exchange smells to share information, thus maintaining the diversity of the population. Finally, the bi‐objective fruit fly optimization algorithm completes the dynamic ensemble pruning under the condition that iterative updates are stopped and pruning sets are outputs to calculate the final clusters using a consensus clustering algorithm.

### 2.2 DEPF can Provide Better Performance than Several Single‐Cell Clustering Algorithms

**Figure 2:**
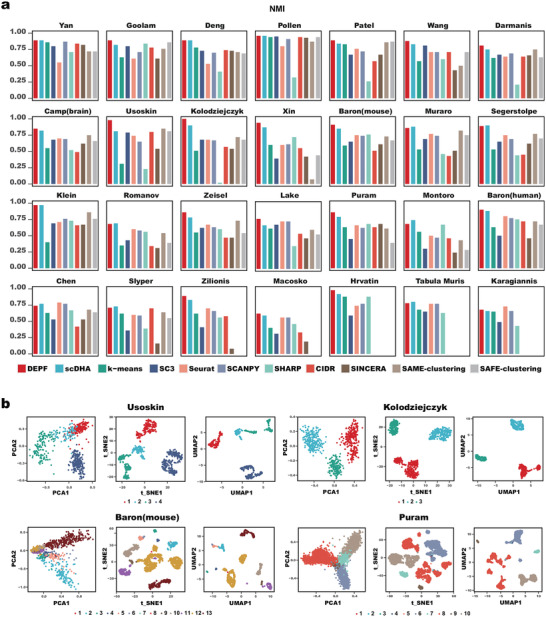
a) Clustering performance of DEPF, and 10 single‐cell clustering algorithms measured by NMI on 28 real scRNA‐seq datasets. b) 2D visualization of DEPF‐processed Usoskin, Kolodziejczyk, Baron(mouse), and Puram datasets using PCA, t‐SNE and UMAP with labels obtained from DEPF.

DEPF was compared with ten single‐cell clustering algorithms, including scDHA, k‐means, SC3, Seurat, SCANPY, SHARP, CIDR, SINCERA, SAME‐clustering, and SAFE‐clustering on 28 real scRNA‐seq datasets. **Figure** **2**a shows the Normalized Mutual Information (NMI) values obtained by comparing the predicted labels with the true partitioning. As depicted in Figure 2a and Table S1 (Supporting Information), DEPF has the highest NMI values of all the single‐cell clustering algorithms on 20 datasets, and of note, DEPF obtained an NMI value of 0.98 on the Usoskin and Hrvatin datasets and 1 on the Kolodziejczyk dataset. On the Pollen, Xin, Baron(mouse), Klein, Baron(human) datasets, DEPF yielded NMI values greater than 0.9. On Yan, Goolam, Deng, Patel and Zilionis datasets, the results were only one percent less than 0.9. In addition, on the Wang, Darmanis, Camp(brain), Zeisel, and Puram datasets, DEPF obtained NMI values above 0.8. On both the Lake and Macosko datasets, DEPF generated NMI values that exceeded the results of other single‐cell algorithms, although only reaching 0.77 and 0.62, respectively. On the remaining eight datasets, scDHA provided the best results on Muraro, Segerstolpe, Romanov, Montoro, Slyper, and TabulaMuris datasets, while the Seurat method obtained the highest NMI values on the Chen and Karagiannis datasets. Although DEPF performed poorly on these eight datasets, it generally outperformed the other single‐cell clustering algorithms. From the average NMI results, single‐cell ensemble clustering algorithms such as SAME‐clustering and SAFE‐clustering give better results than general clustering algorithms, for example, k‐means and CIDR. For the Adjusted Rand Index (ARI) metric, Table S2 (Supporting Information) reveals that the ARI values for DEPF were comparable to the NMI values on the 28 real scRNA‐seq datasets. The ARI values of DEPF were higher than other single‐cell clustering algorithms on 19 datasets. On the Kolodziejczyk dataset, DEPF generated an ARI value of 1. The number of datasets with ARI values greater than 0.9 was 11. In contrast to the results for NMI, SC3 produced the best ARI value for the Pollen dataset. On the Macosko dataset, DEPF had ARI values that were significantly lower than scDHA and SHARP.

In addition, we adopted Principal Component Analysis (PCA), t‐SNE, and UMAP to visualize the clusters of DEPF. As evident in Figure 2b, there is almost no overlap between the clusters identified by DEPF. Although the visualization after PCA is not as good as that after t‐SNE and UMAP, the outline of each cluster is still clear. In summary, DEPF performed competitively on 28 scRNA‐seq datasets of varying sample size when compared to ten other single‐cell clustering algorithms.

### 2.3 DEPF Performs Better than Several Deep Clustering Algorithms

**Figure 3:**
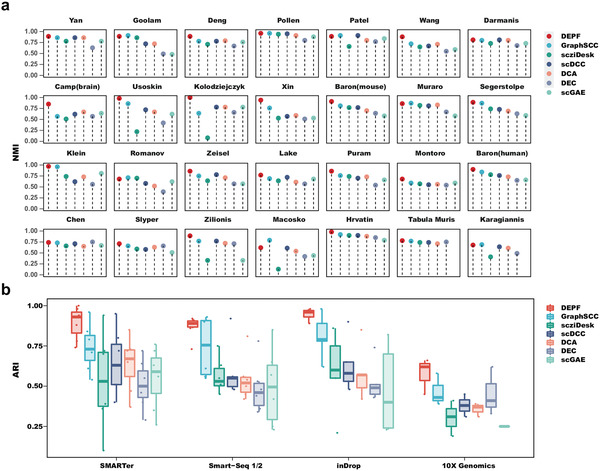
a) Clustering performance of DEPF and 6 deep clustering algorithms measured by NMI on 28 real scRNA‐seq datasets. b) Comparisons of ARI values between DEPF and 6 deep clustering algorithms across SMARTer, Smart‐Seq 1/2, InDrop, and 10X Genomics platforms.

Of the 28 real scRNA‐seq datasets analysed, these datasets across 9 platforms including SMARTer, Smart‐Seq 1/2, inDrop, 10X Genomics, Drop‐seq, STRT‐Seq, Fluidigm C1, Tang, and CEL‐Seq2. To evaluate the effect of several data sources, we first compared DEPF with six deep clustering algorithms including GraphSCC, scziDesk, scDCC, DCA, DEC, and scGAE on the 28 real scRNA‐seq datasets. **Figure** **3**a and Table S3 (Supporting Information) indicate that the NMI values obtained by DEPF on 21 datasets are much higher than those of the other deep clustering algorithms. For example, on the Kolodziejczyk dataset, DEPF has an NMI value of 1. The methods ranking second, scDCC, DCA and scGAE obtained NMI values of only 0.78, and the scziDesk method has the lowest NMI value of 0.08. In addition, as the TabulaMuris and Karagiannis datasets exceed 50k, scGAE requires more memory than the set value, so scGAE cannot manage clustering of this data. Then for data platform, DEPF showed higher NMI values than the other deep clustering algorithms on the datasets of inDrop, STRT‐Seq, Tang, and Fluidigm C1 platforms. In contrast to the results of ARI, the NMI values of DEPF are higher than the other deep clustering algorithms on the Pollen and Darmanis datasets of the SMARTer platform and Segerstolpe dataset of the Smart‐Seq 1/2 platform. In addition, on the Chen dataset of the Drop‐seq platform, DEPF obtained an NMI value of 0.74, which is only one percentage point smaller than DEC. On the inDrop, 10X Genomics, STRT‐Seq, CEL‐Seq2, Tang, and Fluidigm C1 platforms, DEPF demonstrated higher clustering performance measured by ARI than the other deep clustering algorithms (see Figure 3b and Table S4, Supporting Information). On the Pollen and Darmanis datasets from the SMARTer platform, as well as the Patel and Segerstolpe datasets from the Smart‐Seq 1/2 platform, DEPF did not have the highest ARI values, but the difference to the best results were less than 2%. In conclusion, DEPF tends to show better clustering performance than other deep clustering algorithms on datasets from different platforms. In addition, we investigated the performance of DEPF in correcting for batch effects by cascading four publicly human pancreas datasets (see Section S5, Supporting Information).

### 2.4 DEPF is Superior to Several Ensemble Clustering Algorithms

**Figure 4:**
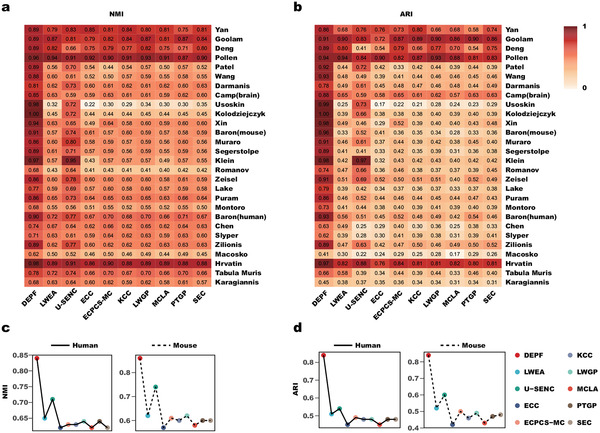
a,b) Clustering performace of DEPF and 9 ensemble clustering algorithms measured by NMI and ARI on 28 real scRNA‐seq datasets. c,d) Comparisons of NMI and ARI values between DEPF and 9 ensemble clustering algorithms across Human and Mouse.

We also compared DEPF with nine ensemble clustering algorithms including LWEA, U‐SENC, ECC, ECPCS‐MC, KCC, LWGP, MCLA, PTGP and SEC in the species perspective. From **Figure** **4**a,b, we see clearly that DEPF has higher NMI and ARI values on nearly all 28 datasets compared to the other algorithms. According to the algorithm's NMI score on each data set shown as colored boxes (the darker the color the closer the score is to 1), the nine ensemble clustering algorithms perform better on the Yan, Goolam, Deng, Pollen and Hrvatin datasets than on the other datasets, but performance is never as good as DEPF. The Yan dataset is human embryo, the Pollen dataset human tissue, the Deng dataset mouse embryo, the Goolam dataset mouse embryo and the Hrvatin dataset mouse visual cortex. From Figure 4c,d and Tables S5– S8 (Supporting Information), DEPF produces a mean value of 0.84 for NMI and ARI on the human datasets, which is higher than the other algorithms that yield NMI averages between 0.6 and 0.71, and ARI averages of close to 0.5. DEPF produced a mean NMI of 0.86 and ARI 0.84 on the remaining datasets while the other algorithms only generated a mean NMI of 0.74 and ARI of 0.60. In conclusion, DEPF demonstrated superior clustering performance compared to the other algorithms on data from different species.

### 2.5 The Running Times of DEPF and Other Methods

**Figure 5:**
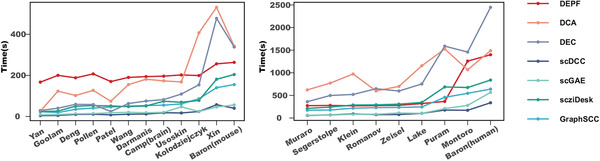
Running time of DEPF and six deep clustering algorithms.

The runtime of DEPF was compared to the other deep clustering algorithms, including DCA, DEC, scDCC, scGAE, scziDesk and GraphSCC. For a fair comparison, all algorithms were executed on a unified platform consisting of Ubuntu 18.04.6 LTS, an Intel (R) Xeon (R) Gold 5220 CPU clocked at 2.20GHz, and a Quadro RTX 6000 GPU. From **Figure** **5**, we observe that the scDCC algorithm has the shortest runtime whereas graphSCC and scGAE algorithms have approximately the same runtimes. DEC and DCA have the longest runtimes. DEPF, DCA and DEC algorithms take longer to execute as the data size increases. When the data size is less than 6000 (Puram dataset: 5902 cells), the increase in DEPF's execution time is relatively smooth. However, when the data size exceeds 7000 (Montoro dataset: 7193 cells), DEPF runtime dramatically increases. This could be because DPEF generates multiple candidate solutions for the bi‐objective function evaluation when performing dynamic integral pruning, and the increase in running time is proportional to the population size and number of evaluations.

### 2.6 Evaluations on Large Scale scRNA‐seq Data >100k

**Figure 6:**
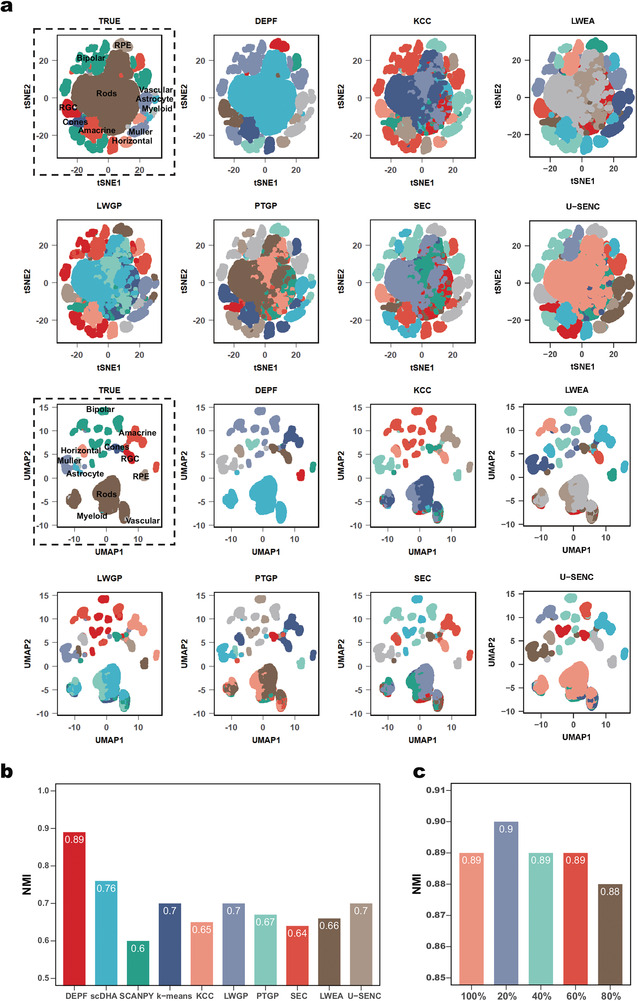
a) Color‐coded representation of the Orozco dataset using t‐SNE and UMAP. b) Clustering performance of DEPF and nine clustering algorithms measured by NMI. c) The change of NMI values from whole data to 20%, 40%, 60%, and 80% downsampling data in Orozco dataset.

We evaluated the clustering performance of DEPF on the Orozco dataset that has a cell number greater than 100k. The Orozco dataset is sampled from the human eye, using the 10X Genomics platform and contains 100055 cells and 11 truth clusters (Amacrine, Astrocyte, Bipolar, Cones, Horizontal, Muller, Myeloid, RGC, Rods, RPE, and Vascular cells). DEPF was initially compared to nine other methods, including scDHA, SCANPY, k‐means, KCC, LWGP, PTGP, SEC, LWEA, and U‐SENC. The NMI metric was used to evaluate clusters identified by the methods. t‐SNE and UMAP were employed to compare visually the true and predicted clustering labels of the algorithms. In **Figure** **6**a using color‐coded representation, we see that predicted labels of DEPF are closer to the actual labels than for the other methods. As depicted in Figure 6b, DEPF has the highest NMI value of 0.89, while none of the other methods has higher value than 0.8. We also observe that DEPF outperforms LWEA and U‐SENC in terms of NMI on the Orozco dataset. Moreover, comparing LWEA, U‐SENC, KCC, LWGP, PTGP and SEC reveals that U ‐SENC and LWGP have the highest NMI values, while PTGP, LWEA, and KCC have NMIs of 0.67, 0.66, and 0.65, respectively, and SEC the worst performance.

Furthermore, we evaluated the running time, CPU time and memory usage of DEPF on the Orozco dataset. First, we tested the respective running time, CPU time and memory usage of DEPF at different cell numbers. Indeed, we randomly selected sample rates of 20%, 40%, 60%, and 80% to test the running time, CPU time and memory usage for different numbers of cells. The results of running time, CPU time and memory usage are summarized in Figures S1 and S2 (Supporting Information). As depicted in Figures S1 and S2 (Supporting Information), we observe that the maximum running time and memory usage for the unsampled Orozco dataset are feasible for most personal computers.

Then, we compared DEPF with four other methods, including a non‐ensemble graph‐based clustering algorithm (Seurat), two ZINB model‐based autoencoder clustering algorithms (DCA and scziDesk), and a deep neural network clustering algorithm (DEC). Figure S3a (Supporting Information) shows the overall running time of DEPF with four other non‐ensemble graph‐based clustering algorithms. We can clearly observe that the running time of the traditional method, Seurat, exhibits faster running times than all deep learning approaches. Among the deep learning methods, our proposed model demonstrates shorter running times, while DEC has the longest running time. Figure S3b (Supporting Information) illustrates the memory consumption of DEPF with four other non‐ensemble graph‐based clustering algorithms. Consistent with the running time results for the Orozco dataset, Seurat consumes less memory than its counterparts. In terms of memory usage, our algorithm remains the least resource‐intensive among the deep learning methods. Although DEPF does not have the shortest running time or the smallest memory footprint, Figure S3c (Supporting Information) reveals that it achieves higher NMI and ARI values compared to the other four algorithms, suggesting its superior performance in clustering quality.

In our study, DEPF consists of four modules: Normalization, Hierarchical Autoencoder, Clustering Ensemble, and Dynamic Ensemble Pruning. The Normalization module scales expression data to a range of 0–1 for each cell, while the Hierarchical Autoencoder module maps normalized data to multiple low‐dimensional latent spaces. The Clustering Ensemble module addresses non‐linear embedding in the latent space using an effective basic clustering algorithm, generating multiple underlying cluster results to create cluster ensembles. Lastly, the Dynamic Ensemble Pruning module dynamically eliminates low‐quality basic clusterings in the ensemble to improve overall clustering accuracy. To evaluate the variation in memory usage and running time across different DEPF steps, we measured the running time and memory consumed during each step when processing the Orozco dataset, as depicted in Figure S3d,e (Supporting Information). We can observe that the Hierarchical Autoencoder and the Clustering Ensemble module require the most running time. The reason is that the Hierarchical Autoencoder, which includes a non‐negative kernel autoencoder and a stacked Bayesian autoencoder for dimensionality reduction, occupies the largest proportion of memory usage. Additionally, DEPF generates a substantial clustering ensemble to optimize clustering results, leading to the longest running time, where the ensemble size is controlled by a hyper‐parameter *T*, which can be adjusted by the users. To further demonstrate that the impact of the hyperparameter *T* on the running time and memory consumption of the algorithm, we constructed an experiment to discuss the performance of different hyperparameters *T*. Figure S3f (Supporting Information) shows the running time of the Clustering Ensemble module and the NMI and ARI values obtained by DEPF under different *T*. The results indicate that when *T* is set to 1, the running time of the module is only 233 seconds. Therefore, when there is a requirement for running time, adjusting the *T* can reduce the running time.

### 2.7 Dynamic Ensemble Pruning: Many could be Better than All

**Figure 7:**
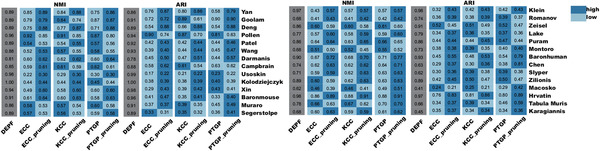
Clustering performance of ECC pruning, KCC pruning and PTGP pruning on 28 real scRNA‐seq datasets measured by NMI and ARI.

Dynamic ensemble pruning is a core model in DEPF and it may affect the clustering power of DEPF by removing unneeded costs incurred by clusters in the ensemble. We integrated dynamic ensemble pruning into the ensemble clustering algorithms of ECC (ECC pruning), KCC (KCC pruning), and PTGP (PTGP pruning) to investigate the portability of dynamic ensemble pruning on the 28 real scRNA‐seq datasets using NMI and ARI metrics to evaluate the clustering performance. From **Figure** **7**, we find that dynamic ensemble pruning has the greatest effect on the ECC technique. The NMI values obtained by ECC pruning were greater than the NMI values of the original ECC method on 19 datasets, and ARI values greater on 24 datasets. Compared to the original KCC algorithm, the KCC pruning obtains higher NMI values on 18 datasets and higher ARI values on 16 datasets. Compared to the original PTGP, the PTGP pruning method has higher NMI values on 19 datasets and higher ARI values on 17 datasets. Although none of the integrated pruning clustering algorithms outperformed the corresponding original algorithm across all datasets, results on approximately 20 out of 28 datasets demonstrated that dynamic ensemble pruning directly added to a consensus clustering algorithm enhanced the clustering performance of the original method.

### 2.8 Impact of the Bi‐Objective Function with the Optimization Direction

We added a directed bi‐objective function to DEPF; therefore, it is essential to determine the direction of optimization of the bi‐objective function for various scRNA‐seq datasets. To demonstrate the efficacy of a bi‐objective function with optimized directions, we compared five different versions of DEPF: original DEPF, a directionless DEPF, an anti‐directional DEPF and two single‐objective DEPFs (Cp‐DEPF, Dev‐DEPF), where directionless means that the direction of the bi‐objective function is only positive (“+”) and inverse means that the direction is opposite to that of the original DEPF (in the opposite direction from “+” to “‐” and “‐” to “+”). NMI metric was employed to evaluate the performance of the different versions of DEPF on the 28 scRNA‐seq datasets.

**Figure 8:**
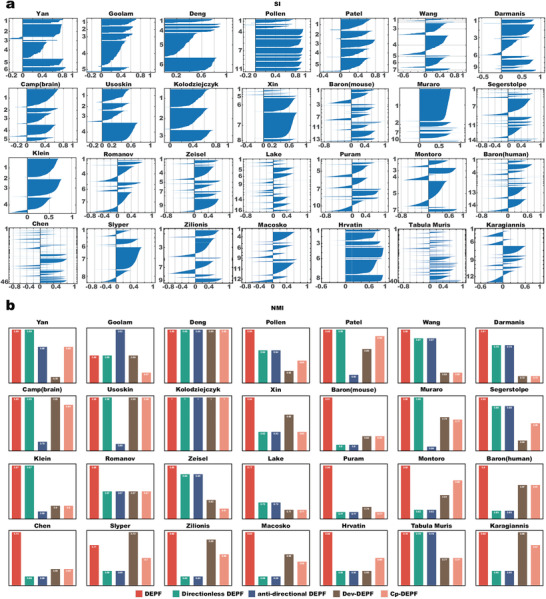
a) The value of the silhouette coefficient on 28 real scRNA‐seq datasets. b) Clustering performance of DEPF, the directionless DEPF, anti‐directional DEPF, Cp‐DEPF, Dev‐DEPF on 28 real scRNA‐seq datasets measured by NMI.

As demonstrated in **Figure** **8**a, we calculated the silhouette coefficients of each cell in the initial clustering result on the 28 scRNA‐seq datasets. With the exception of the Deng dataset, the silhouette values are less than 0 for the other 27 datasets. The closer the silhouette value is to ‐1, according to the definition of the silhouette coefficient, the more likely it is that the cell does not belong to the cluster it is in. Consequently, additional optimization of the initial clustering was required. Initially, the effect of direction was validated by comparing the original DEPF to directionless DEPF. In other aspects, from Figure 8b, the comparative results show that using the silhouette coefficient as an optimization direction for the bi‐objective function is an effective way to improve clustering accuracy. On the Yan, Goolam, Deng, Patel, Camp(brain), Usoskin, Kolodziejczyk, Muraro, and Klein datasets, original DEPF and directionless DEPF obtained equal NMI values due to the “+” direction of both. On the other 19 datasets, the “‐” direction resulted in better NMI values for original DEPF than the NMI values for directionless DEPF with the “+” orientation.

Furthermore, to illustrate that the direction is considered as “‐” when the silhouette value in the dataset is less than a threshold (ϵ) value (i.e., ‐0.6), and otherwise the direction is considered “+”, a comparison between the original DEPF and the anti‐directional DEPF (where the direction is considered “‐” when the silhouette value in the dataset is greater than ‐0.6) was performed on the 28 datasets. As depicted in Figure 8b, the NMI values obtained by the original DEPF are higher on 27 datasets but not on the Goolam dataset where the NMI value of anti‐directional DEPF is superior to that of the original DEPF. Based on the comparison of original DEPF, directionless DEPF and anti‐directional DEPF, it is better if the ϵ for determining direction is set to ‐0.6. From Figure 8a, the minimum silhouette value is approximately ‐0.2 on Yan, Camp(brain) and Usoskin datasets. On these datasets, the original DEPF and directionless DEPF have higher NMI values when the direction is set to “+”, which means that the ϵ should be set to less than ‐0.3. On 13 datasets such as Baron(mouse), as the minimum silhouette value is about ‐0.8 and the DEPF with “‐” direction has the highest NMI value, the ϵ value needs to be greater than ‐0.8. On Wang, Hrvatin and Karagiannis datasets, the minimum silhouette value is less than ‐0.6 but greater than ‐0.7. The NMI value obtained by the original DEPF is higher the direction ‘‐’. On the Patel dataset, the minimum silhouette value is between ‐0.4 and ‐0.5 and the direction is “+”. Therefore, integrating the different scenarios above, a ϵ setting of ‐0.6 enables the original DEPF to show better optimization performance on most datasets. In addition, the influence of each objective function on DEPF is considered. From Figure 8b, the highest NMI values are obtained by the original DEPF on 27 datasets except Slyper, where the highest NMI value is seen for the Dev‐DEPF.

In summary, as DEPF is an unsupervised algorithm, there is no guarantee that DEPF will yield the highest NMI values on all 28 scRNA‐seq datasets. However, on the whole, the bi‐objective function and the optimization direction are important guarantees of DEPF clustering performance.

### 2.9 DEPF can Characterize scRNA‐seq Data at Finer Resolutions

**Figure 9:**
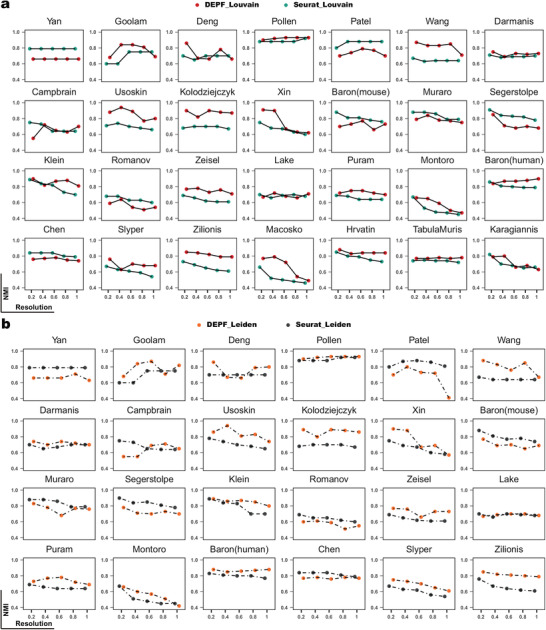
a) Clustering performance of *DEPF*_*Louvain*_ and *Seurat*_*Louvain*_ measured by NMI on 28 real scRNA‐seq datasets. b) Clustering performance of *DEPF*_*Leiden*_ and *Seurat*_*Leiden*_ measured by NMI on 24 real scRNA‐seq datasets.

To better characterize subtypes or different cell states of the same cell type, we have replaced the basic clustering algorithm in our DEPF algorithm with the Louvain and Leiden algorithms. This is because the spectral clustering algorithm used in DEPF does not have a resolution parameter, which is necessary for achieving a finer level of clustering. By using the Louvain and Leiden algorithms, we can adjust the resolution parameter and more accurately identify distinct subtypes or cell states within a given cell type. To investigate the robustness of the estimated performance at different clustering resolutions, we conducted an experiment to compare the clustering performance of *DEPF*_*Louvain*_, *DEPF*_*Leiden*_, *Seurat*_*Louvain*_, and *Seurat*_*Leiden*_ at different resolutions ranging from 0.2 to 1.0 on those twenty‐eight real scRNA‐seq datasets with known true labels. **Figure** **9** presents that the robustness of *DEPF*_*Louvain*_ and *DEPF*_*Leiden*_ is better than that of *Seurat*_*Louvain*_, and *Seurat*_*Leiden*_, respectively. From Figure 9a, it is evident that *DEPF*_*Louvain*_ with different resolutions outperforms *Seurat*_*Louvain*_ in terms of NMI values on nearly 20 datasets. Specifically, on Pollen, Wang, Darmanis, Usoskin, Kolodziejczyk, Xin, Zeisel, Puram, Montoro, Slyper, Zilionis, Macosko, Harvatin, and TabulaMuris datasets, *DEPF*_*Louvain*_ has higher NMI values at any resolutions. Among them, the results for the Wang, Usoskin, Kolodziejczyk, Zilionis, and Macosko datasets demonstrate that the difference in NMI values between *DEPF*_*Louvain*_ and *Seurat*_*Louvain*_ was almost 0.2 for partially different resolutions. Figure 9b indicates that *DEPF*_*Leiden*_ with different resolutions achieves the highest NMI values across the 17 scRNA‐seq datasets. Specifically, on Pollen, Wang, Darmanis, Usoskin, Kolodziejczyk, Xin, Baron(mouse), Klein, Zeisel, Puram, Montoro, Baron(human), Slyper, and Zilionis datasets, *DEPF*_*Leiden*_ outperforms *Seurat*_*Leiden*_ at any resolutions. Among them, the results for the Wang, Kolodziejczyk and Zilionis datasets show that the partially different resolutions makes a difference of nearly 0.2 NMI values between *DEPF*_*Leiden*_ and *Seurat*_*Leiden*_.

Based on the above analyis, we can observe that since the mechanism of Louvain and Leiden is that a larger resolution results in a larger number of clusters, it leads to fluctuations in the clustering performance of the algorithm itself. As a result, the NMI values of *DEPF*_*Louvain*_ and *DEPF*_*Leiden*_ exhibit different variations on different datasets. From the results of all datasets, the trend of NMI values with increasing resolution for (*DEPF*_*Louvain*_, *DEPF*_*Leiden*_) and (*Seurat*_*Louvain*_, *Seurat*_*Leiden*_) is consistent, and the former usually leads to higher NMI values; for instance, the NMI values of all four algorithms decrease with increasing resolution on Segerstolpe, Montoro and Zilionis datasets, while on Goolam and Patel datasets, the NMI values of the four algorithms first increase and then decrease as the resolution increases. Notably, on the Baron(human) dataset, the NMI values of *DEPF*_*Louvain*_ and *DEPF*_*Leiden*_ gradually increase with increasing resolution, while the NMI values of *Seurat*_*Louvain*_ and *Seurat*_*Leiden*_ gradually decrease. In summary, the Louvain and Leiden can be used as the basic clustering algorithms in DEPF. In particular, the DEPF embedded with the Louvain and Leiden algorithms demonstrated excellent robustness and clustering performance on those scRNA‐seq datasets, and has the potential to be able to characterize subtypes of the same cell type or different cell states.

### 2.10 DEPF can Identify Rare Cell Types and Small Clusters that Would not be Picked up by Other Methods

**Figure 10:**
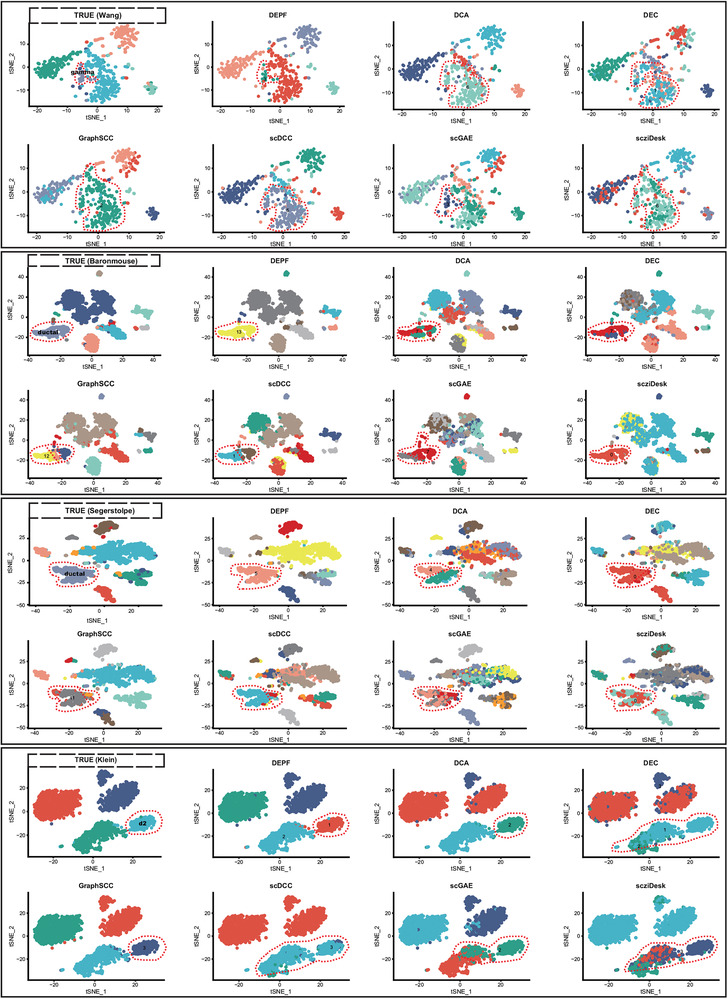
Color‐coded representations of the Wang, Baron(mouse), Segerstolpe, and Klein datasets with labels obtained from true, DEPF, DCA, DEC, GraphSCC, scDCC, scGAE, and scziDesk, respectively.

To investigate whether DEPF can detect rare cell types and small clusters not detected by other methods, we compared DEPF with six other methods, including DCA, DEC, GraphSCC, scDCC, scGAE, and scziDesk, on the Wang, Baron(mouse), Segerstolpe, and Klein datasets that contain rare cell types and small clusters for in‐depth examination. To construct a fair and unambiguous comparison, we first applied t‐SNE to project the raw single‐cell data into a 2D space and visualized it using real labels. After that, we applied DEPF and the other deep learning methods (including DCA, DEC, GraphSCC, scDCC, scGAE, and scziDesk) to obtain the clustering labels for visualization in the same 2D space. The experimental results are summarized in **Figure** **10**. As indicated in this figure, we observe that DEPF can detect rare cell types and small clusters that the other methods do not detect on these datasets; for example, on the Wang dataset, DEPF accurately identifies gamma cells, while the other algorithms mix them with other cells. On the Baron(mouse) and Segerstolpe datasets, DEPF successfully delineated ductal cells, while the other algorithms performed poorly in identifying ductal cell clusters. DCA, DEC, scDCC, scGAE, and scziDesk divided ductal cell clusters into two parts, while GraphSCC separated ductal cell clusters into three parts. In addition, DEPF, DCA, and GraphSCC effectively identified d2 clusters on the Klein dataset, whereas DEC, scDCC, scGAE, and scziDesk include cells within other clusters in d2 clusters. Overall, our proposed DEPF outperforms or complements existing methods in identifying cell types and reliably detects rare cell types and small clusters.

### 2.11 DEPF can Identify Novel Clusters that Other Traditional Methods Failed to Detect

To test whether DEPF can identify novel clusters that other traditional methods fail to detect, we applied DEPF to a colorectal cancer (CRC) dataset. This dataset is derived from the GSE144735 dataset in the NCBI Gene Expression Omnibus database and contains 27414 cells from the tumor. In their original research^[^ ^20^ ^]^ to identify different cell types, Seurat was employed to cluster the cells using a graph‐based clustering algorithm and then subsequently annotating the different clusters by SingleR^[^ ^21^ ^]^ and obtained marker genes from the different cluster groups. This resulted in six different cell types, including Epithelial cells, Myeloid cells, T cells, B cells, Stromal cells and Mast cells. To demonstrate the effectiveness of our proposed DEPF, we first used Seurat to preprocess the dataset according to the following criteria: *min.cells*=3, *min.features*=200, *nFeature_RNA* >50 and <5% of mitochondrial gene expression in UMI counts. From the filtered cells, the gene expression matrices were normalized to the total UMI counts per cell and transformed to the natural log scale.

**Figure 11:**
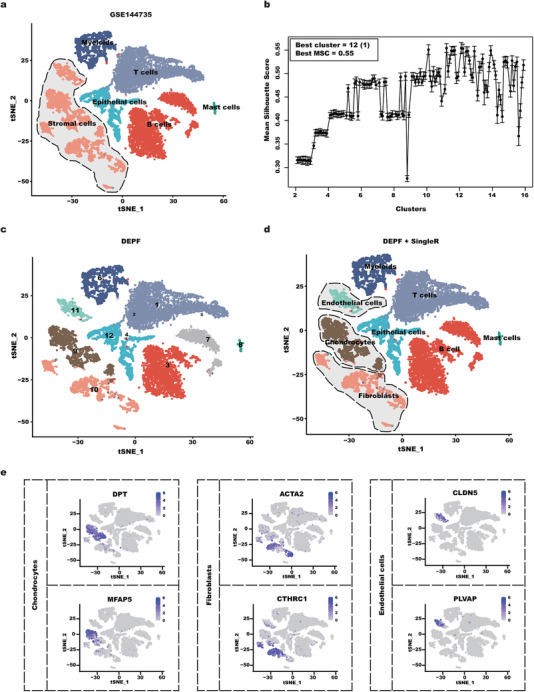
a) Color‐coded representation of the GSE14473 dataset using Seurat's standard process and t‐SNE with labels obtained from the true information. b) The value of MSC on different clusters obtained by the DEPF. c) Color‐coded representation of the CRC cells using t‐SNE with labels obtained from DEPF. d) Color‐coded representation of the CRC cells using t‐SNE with labels obtained from DEPF+singleR. e) The expression distribution of DPT and MFAP5 in the Chondrocytes cluster, ACTA2 and CTHRC1 in the Fibroblasts cluster, and CLDN5 and PLVAP in the Endothelial cells cluster.

Then, to estimate a suitable clustering number for this dataset, we first obtained different label distributions by specifying 14 swarms on DEPF for an iterative search of the cluster space from 2 to 15. After completing the iterations, the mean silhouette coefficient (MSC) was used to evaluate the labels found by the fruit flies. The results are shown in **Figure** **11**b. As depicted in this figure, we find that the highest value of MSC is 0.55 when the number of clusters is 12. Figure 11c provides a 2D visualization of DEPF by t‐SNE for the CRC dataset of 12 clusters. For a fair analysis, we also adopted the singleR method to annotate those different clusters, resulting in eight different cell types, including Epithelial cells, Myeloid cells, T cells, B cells, Mast cells, Chondrocytes, Fibroblasts, and Endothelial cells, as shown in Figure 11d. In contrast to the annotation of Figure 11a, we clearly observe that stromal cells are divided into three different subgroups, including chondrocytes, fibroblasts and endothelial cells, which also shows that the SingleR model could only annotate a very broad cell type (stromal cells) due to the insufficient refinement of the cell clustering results, while DEPF clusters the original stromal cell fraction into different clusters, providing a more refined cell type (chondrocytes, fibroblasts and endothelial cells) during SingleR annotation, thus indicating that our algorithm can identify more cell types.

In addition, to confirm whether these newly‐discovered cell types are consistent with previous findings, we interpreted the top two marker genes of each cell type determined by the Wilcoxon Rank Sum test,^[^ ^9^ ^]^ including their expression distribution in all cells. Figure 11e shows the expression distribution of DPT and MFAP5 in Chondrocytes cells, ACTA2 and CTHRC1 in Fibroblasts cells, and CLDN5 and PLVAP in Endothelial cells. From the figure, it can be seen that these differentially expressed genes were mainly distributed in the appropriate clusters. In addition, we manually matched the marker genes from different cell types in a cell marker database called CellMarker.^[^ ^22^ ^]^ We discovered that those marker genes can be matched with previously published marker genes for the corresponding cell type, demonstrating the feasibility of stromal cells being identified as three distinct subgroups: including chondrocytes, fibroblasts and endothelial cells.

**Figure 12:**
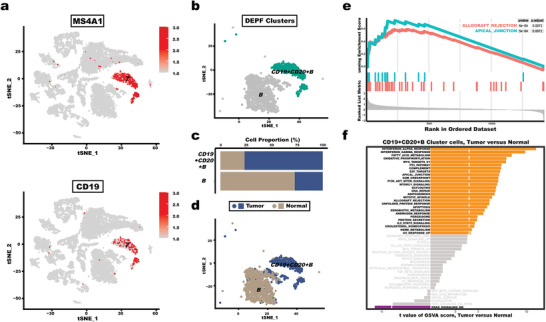
Cluster 7. a) The expression distribution of MS4A1 and CD19 in the cluster 7. b) Color‐coded representation of the CD19+CD20+B cells using t‐SNE. c) Differences in Cell Proportions of Normal and Tumor in CD19+CD20+B and B cells. d) Color‐coded representation of the Normal and Tumor using t‐SNE. e) GSEA on Hallmark pathways for tumor and normal cells. f) Differences in pathway activities scored per cell by GSVA, between tumor and normal cells in CD19+CD20+B. T values are from a linear model.

Indeed, we can observe that clusters 2 and 4 contained only 2 and 5 cells, respectively, rendering them unsuitable for further downstream biological significance analysis. It is possible that the number of clusters in the dataset estimated by our clustering algorithm is high, resulting in some clusters with a low number of cells. Therefore, we targeted to cluster 7 for a more detailed investigation to provide a richer comprehensive assessment of DEPF performance. For cluster 7, the Wilcoxon Rank Sum test was conducted to identify the differentially expressed genes between cluster 7 and other clusters. **Figure** **12**a shows the expression distribution of MS4A1 and CD19 in all clusters. As depicted in this figure, it can be seen that MS4A1 and CD19 were mainly distributed in the cluster 7. According to the previous study,^[^ ^23^ ^]^ we can find that MS4A1, the gene encoding B cell surface marker CD20, is significantly downregulated in human colorectal carcinoma. In addition, CD19 is closely associated with colorectal cancers as a marker of B cells.^[^ ^24^ ^]^ Therefore, distinguishing from the B cell types obtained from cluster 3 annotation, we labeled cluster 7 as CD19+CD20+B, as indicated in Figure 12b.

Figure 12c demonstrates that the percentage of cells with tumor in the CD19+CD20+B is 75% based on the information of tumor versus normal cells provided by the GSE144735 dataset and Figure 12d shows the distribution of tumor and normal cells in cluster B and CD19+CD20+B. We can observe that DEPF groups most of the tumor cells associated with B cells into one category. On this basis, it is indicated that the CD19+CD20+B delineated by DEPF is a subpopulation of the B‐cell population, but the underlying information is more relevant to tumor. Further, we did GSEA analysis for tumor cells and normal cells. Figure 12e illustrates two upregulation pathways with the highest enrichment score including apical junction and allograft rejection. The apical junction are the processes significantly enriched in colorectal cancer cells SW480, as evidenced by other colorectal cancer studies.^[^ ^25^ ^]^ In addition, the allograft rejection score have been reported to be associated with the number of infiltrating immune cells associated with the expression of immune checkpoint molecules, such as B cells.^[^ ^26^ ^]^ Then, we used gene set variation analysis (GSVA) to analyze the differences in pathway activities scored per cell using CD+19+CD20+B clusters in colorectal cancer, as depicted in Figure 12f. We found that Interferon‐α response pathway is a major enrichment signature for tumor cells compared to normal cells. In particular, Interferon‐α response is a signaling pathway that is triggered by the activation of immune cells, including B cells. This pathway plays a critical role in the regulation of the immune response to cancer cells by promoting the activation of T cells and the production of pro‐inflammatory cytokines. Thus, the enrichment of interferon‐α response in tumor cells may suggest that B cells are involved in the anti‐tumor immune response in colorectal cancer through the production of interferon‐α. In addition, previous studies have demonstrated that Interferon‐α is one of the human type I interferons that have been used in a variety of cancer treatments, including some B‐ and T‐cell lymphomas and certain solid tumors.^[^ ^27^ ^]^ Therefore, these findings suggest that targeting the interferon‐α response pathway may be a potential therapeutic strategy for enhancing the anti‐tumor immune response in colorectal cancer.

**Figure 13:**
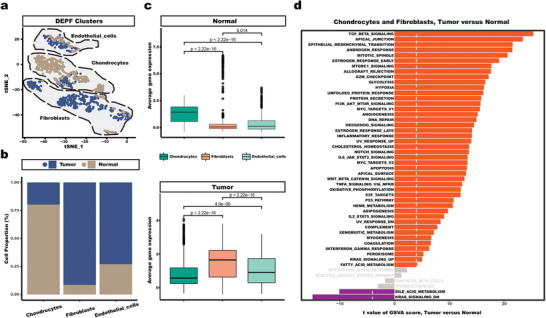
fibroblasts, chondrocytes, and endothelial cells. a) Color‐coded representation of the Normal and Tumor in fibroblasts, chondrocytes, and endothelial using t‐SNE. b) Differences in Cell Proportions of Normal and Tumor in fibroblasts, chondrocytes, and endothelial cells. c) Average gene expression for tumor and normal from fibroblasts, chondrocytes, and endothelial. d) Differences in pathway activities scored per cell by GSVA, between fibroblasts and chondrocytes. T values are from a linear model.

In addition, we also conducted a detailed analysis of stromal cells using the DEPF algorithm, which categorizes them into three distinct clusters, namely fibroblasts, chondrocytes, and endothelial cells. **Figure** **13**a,b shows the distribution and percentage of tumors and normal cells in fibroblasts, chondrocytes, and endothelial cells, respectively, based on the cellular information. Specifically, tumors account for nearly 80% of fibroblasts, while 90 percent of chondrocytes are composed of normal cells. Meanwhile, the results in Figure 13c show that fibroblasts have the highest average gene expression in tumors and chondrocytes obtain the highest average gene expression in normal cells. Subsequently, we performedGSVA on chondrocytes and fibroblasts, as tumor and normal cells, respectively, as depicted in Figure 13d. The results revealed that TGF‐beta signaling was the most enriched signature in tumor fibroblasts cells. Notably, TGF‐beta signaling pathway have been reported to associate with the cancer, which exhibits tumor‐suppressive effects in the early stages of cancer by inhibiting cell cycle progression and promoting apoptosis, and exerts a tumor‐promoting effect, increasing tumor aggressiveness and metastasis in advanced stages.^[^ ^28^ ^]^

## 3 Functional Genomic Analysis

We performed several functional genomic analyses to demonstrate the biological significance of the results obtained from our proposed algorithm. First, we describe the biological insights gained from DEPF clustering of the head and neck squamous cell carcinoma (HNSCC) dataset^[^ ^29^ ^]^ that consists of 6000 single cells from 18 patients. Indeed, HNSCC is a head and neck cancer derived from the mucosal epithelium of the larynx, oral cavity and pharynx. Currently, most patients get diagnosed with late‐stage HNSCC due to the absence of clinically significant pre‐malignant lesions.^[^ ^30^ ^]^ A portion of the cells from the 18 HNSCC patients expressed a partial epithelial‐to‐mesenchymal transition, which is thought to be the driver of the spread of epithelial tumor. The cells of partial epithelial‐to‐mesenchymal transition localize to the leading edge of the primary tumor in proximity to the cancer‐associated fibroblasts.^[^ ^30^ ^]^ Therefore, to facilitate analysis, we targeted the fibroblast cells of the HNSCC dataset.

### 3.1 DEPF can Provide Biological Interpretation of scRNA‐seq Data

**Figure 14:**
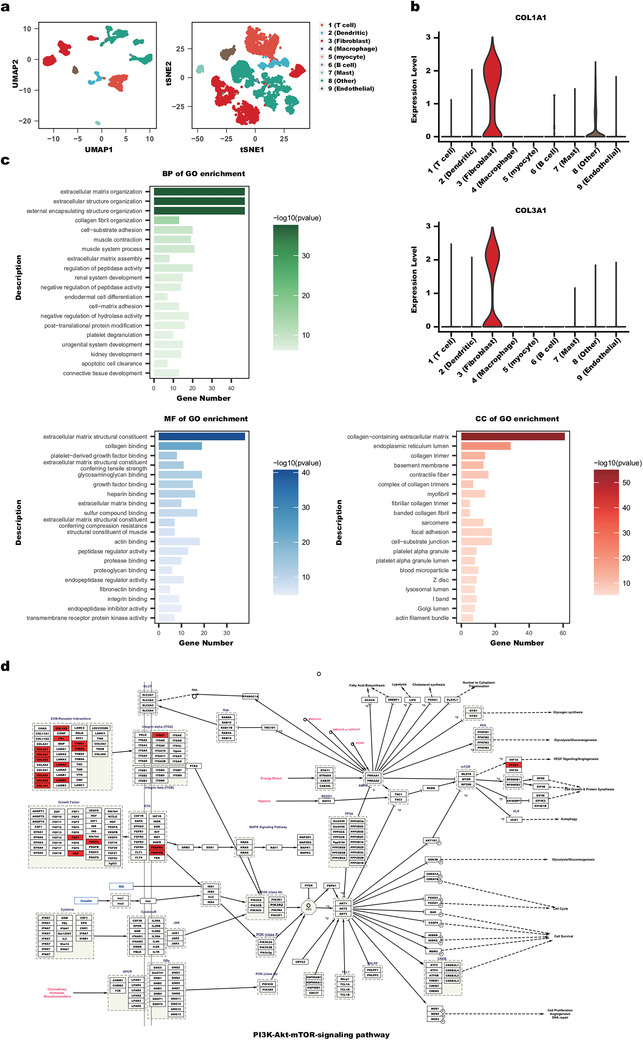
a) Color‐coded representation of the HNSCC data using t‐SNE and UMAP. b) The expression levels of COL1A1 and COL3A1 in the different clusters. c) Gene ontology enrichment analysis. d) Focal adhesion: PI3K‐Akt‐mTOR‐signaling pathway. In the schematic of the pathway, the rectangular boxes indicate genes, while the red boxes are the corresponding DEGs.

To delineate the fibroblast cells from the other 6000 single cells of the HNSCC dataset, our proposed DEPF was first used to perform unsupervised clustering of the HNSCC data. The clustering results are shown in the **Figure** **14**a. Then, based on the predicted labels obtained from DEPF, the Wilcoxon Rank Sum test was conducted using the FindAllMarker function in the Seurat package to identify the top‐200 differentially expressed genes (DEGs) between fibroblast cells and other cells. In addition, we employed other clustering methods and used the same method to extract functional genes to compare with DEPF (Section S7, Supporting Information). From the experimental results, we observed that DEPF is able to selects genes that are highly expressed in fibroblast clusters, while the other 13 clustering algorithms appeared confused or not found. After that, to describe the biological mechanisms and signaling pathways of the DEGs, we employed clusterProfiler^[^ ^31^ ^]^ for gene ontology enrichment analysis and WikiPathways analysis. Then, we obtained 2364 biological processes (BP), 278 cellular components (CC), and 261 molecular functions (MF). After that, we cut off those 3000 GO terms by the *p*‐value <0.05 && adjusted *p*‐value <0.05 && *q*‐value <0.05 and then obtain 324 BPs, 36 CCs, and 38 MFs, which are considered to be differentially GO functional pathways and are listed in Tables S9– S16 (Supporting Information). Figure 14c summarizes the top 20 terms in the categories BP, MF and CC, sorted by −*log*(*pvalue*). From Tables S9– S16 (Supporting Information) and Figure 14c, we see that at the top of the categories are cellular matrix tissue, containing 47 genes, cellular matrix structural components, involving 38 genes and collagen‐containing cellular matrix, enriched by 61 genes; that is, all related to the cellular matrix, which is very relevant to the human skin dermis and has an important role in extracellular matrix biology.^[^ ^32^ ^]^ In parallel, we fed the top 200 DEGs into WikiPathways, which yielded 10 pathways sorted by *p*‐value with a cut‐off of 0.05 and summarized in **Table** **1**. We find the Focal adhesion: PI3K‐Akt‐mTOR‐signaling pathway as detailed in Figure 14d. In this figure, 19 selected DEGs are from the Focal adhesion: PI3K‐Akt‐mTOR‐signaling pathway. Specifically, 12 of the 19 DEGs, COL3A1, COL4A1, COL4A2,COL5A2, COL6A2, COL1A1, COL1A2, FN1, LAMA4, LAMB2, THBS1, and THBS2 are enriched in the ECM‐Receptor Interactions pathway, where ECM is an abbreviation for extracellular matrix. From this gene ontology enrichment analysis, it is clear that the extracellular matrix is significantly associated with fibroblasts.

**Table 1:** Wikipathways information of the top‐200 DEGs

ID	Description	GeneRatio	pvalue	Gene ID	Count
WP2911	miRNA targets in ECM and membrane receptors	14/118	7.05E‐16	COL1A2/COL3A1/FN1/COL6A2/COL6A1/COL6A3/THBS1 /LAMA4/LAMB2/COL4A2/COL5A2/COL4A1/SDC2/THBS2	14
WP5055	Burn wound healing	16/118	7.49E‐12	TAGLN/DCN/COL1A2/PDGFRB/COL1A1/SPARC/FN1/CXCL12 /CD248/TIMP1/VIM/LGALS1/TIMP2/SFRP2/CCL2/MMP2	16
WP306	Focal adhesion	18/118	6.36E‐10	COL1A2/MYL9/PDGFRB/COL1A1/FN1/COL6A2/THBS1 /LAMA4/ITGA7/LAMB2/COL4A2/COL5A2/PDGFRA /COL4A1/MYLK/THBS2/PDGFA/PGF	18
WP3932	Focal adhesion: PI3K‐Akt‐mTOR‐signaling pathway	19/118	9.15E‐08	COL1A2/COL3A1/PDGFRB/COL1A1/FN1/COL6A2/THBS1 /LAMA4/ITGA7/LAMB2/FGF7/COL4A2/COL5A2/PDGFRA /COL4A1/EPAS1/THBS2/PDGFA/PGF	19
WP3967	miR‐509‐3p alteration of YAP1/ECM axis	6/118	1.59E‐07	COL3A1/COL1A1/SPARC/FN1/EDNRA/THBS2	6
WP4172	PI3K‐Akt signaling pathway	18/118	2.49E‐06	COL1A2/PDGFRB/COL1A1/FN1/COL6A2/COL6A1/COL6A3 /THBS1/LAMA4/ITGA7/LAMB2/FGF7/COL4A2/PDGFRA /COL4A1/THBS2/PDGFA/PGF	18
WP453	Inflammatory response pathway	6/118	4.39E‐06	COL1A2/COL3A1/COL1A1/FN1/THBS1/LAMB2	6
WP5087	Malignant pleural mesothelioma	19/118	2.54E‐05	ACTA2/PDGFRB/ADAMTS1/SERPINF1/SPARC/FN1/CXCL12 /LAMA4/LAMB2/FGF7/COL4A2/SFRP2/CCL2/PDGFRA /COL4A1/MMP2/PDGFA/PGF/CDH6	19
WP4754	IL‐18 signaling pathway	14/118	5.29E‐05	ACTA2/COL1A2/COL3A1/COL1A1/FN1/TIMP3/TIMP1/NR4A1 /CCL2/EPS8/HSPB8/RGS16/IER3/MMP2	14

### 3.2 DEPF can Discover Transcriptional and Post‐Transcriptional regulators in scRNA‐seq data

**Figure 15:**
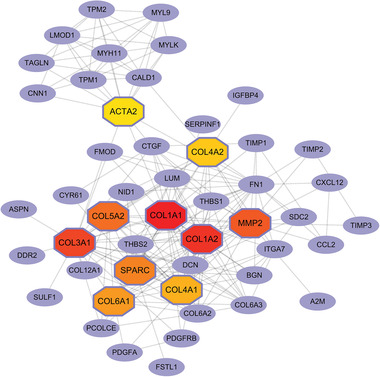
Collection of hub genes from the PPI network using the Cytohubba. Here, the hexagonal‐nodes indicate the top‐10 hub genes.

Next, we constructed a protein–protein interaction (PPI) network of the top‐200 DEGs. The PPI network was generated using the STRING database (https://string‐db.org/) (version 11.5).^[^ ^33^ ^]^ In the network, the confidence value was set to 0.7 and the disconnected nodes were hidden. Then, we input the processed PPI network into Cytoscape (v.3.9.1) to display visually the node‐to‐node connections. Accordingly, we applied Maximal Clique Centrality of cytoHubba (https://apps.cytoscape.org/apps/cytohubba) in the Cytoscape APP Store to recognize the top 10 hub genes from the PPI network. From **Figure** **15**, the top 10 hub genes are COL1A1, COL1A2, COL3A1, MMP2, COL5A2, SPARC, COL6A1, COL4A1, COL4A2, and ACTA2. In addition, we find that the COL1A1 and COL3A1 are marker genes for fibroblasts queried by CellMarker^[^ ^22^ ^]^ also tagged as hub genes, as shown in Figure 14b. Other genes could be potential markers for fibroblasts cells.

**Figure 16:**
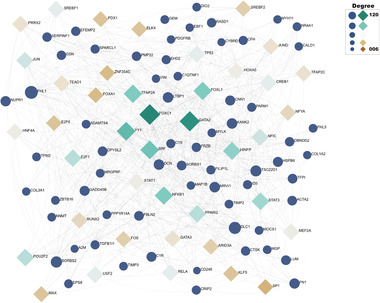
The TFs‐DEGs interaction network.The diamond nodes are TFs, and circle nodes represent genes. The degree refers to the number of edges that are connected to the specified node in the graph. The greater the degree of the node, the greater the scale.

We went on to analyze the relationship between the identified DEGs, transcription factors (TFs) and miRNAs. First, the top 200 DEGs were fed into the NetworkAnalyst platform^[^ ^34^ ^]^ to search for TFs from the JASPAR^[^ ^35^ ^]^ database. The TF‐gene network is set as the minimum network, and the network nodes with a ‘betweenness’ of less than 100 are filtered out to remove the low‐quality TFs. From **Figure** **16**, we observe that the network contains 65 genes and 41 TFs. Among the TFs, we note FOXC1, a regulator of development and function of many organs; SRF that promotes discoidin domain receptor 2 regulation of fibroblast survival and cycle progression;^[^ ^36^ ^]^ and FOXL1, expressed in lung fibroblasts and controlling a series of genes that enhance fibroblast function. FOXL1 is also implicated in the pathogenesis of pulmonary fibrosis.^[^ ^37^ ^]^

**Figure 17:**
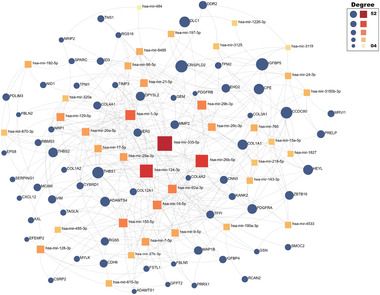
The miRNAs‐DEGs interaction network.The square nodes are miRNAs, and circle nodes represent genes. The degree refers to the number of edges that are connected to the specified node in the graph. The greater the degree of the node, the greater the scale.

We also explored the miRNA‐gene interaction to find potential transcriptional and post‐transcriptional regulators of the common DEGs using networkAnalyst in the mirTarbase (V8.0) database.^[^ ^38^ ^]^ In our study, an miRNA‐gene network was set as the minimum network and network nodes with betweenness less than 800 were filtered out to remove low quality miRNAs. From **Figure** **17**, we observe that the network contains 65 genes and 39 miRNAs. Some miRNAs are present in hypertrophic scars fibroblasts (e.g., hsa‐mir‐124‐3p),^[^ ^39^ ^]^ which essentially indicates a strong interference between them.

We investigated possible target drugs associated with the DEGs using Enrichr (https://maayanlab.cloud/Enrichr/) and the Drug Signature Database.^[^ ^40^ ^]^ **Table** **2** shows the potential top 10 drug molecules ranked according to their *p*‐value. Dasatinib has been used in Ph‐positive acute lymphoblastic leukemia or chronic myelogenous leukemia, and is being studied for use in treatment‐resistant Philadelphia chromosome‐positive leukemia.^[^ ^41^ ^]^ Retinoic acid is a non‐peptide lipophilic small molecule originating from vitamin A that maintains the growth and development of human organs.^[^ ^42^ ^]^ Cytarabine has been proposed as a treatment for acute myeloid leukaemia.^[^ ^43^ ^]^ Trichostatin A has been used to reduce the inflammatory response during polymicrobial sepsis.^[^ ^44^ ^]^ Valproic acid has been used successfully for the treatment of bipolar disorder, schizophrenia and epilepsy.^[^ ^45^ ^]^ In addition, Progesterone, Estradiol and Medroxyprogesterone acetate may be potential subjects for cancer drug research.

**Table 2:** List of the suggested drugs for head and neck squamous cell carcinoma

ID	Term	P‐value	Monoisotopic mass	Molecular formula	Chemical structure
1	Progesterone CTD 00006624	2.75327E‐39	314.224579 Da	*C*_21_ *H*_30_ *O*_2_	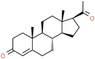
2	Dasatinib CTD 00004330	8.96112E‐27	487.155731 Da	*C*_22_ *H*_26_ *ClN*_7_ *O*_2_ *S*	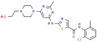
3	Estradiol CTD 00005920	2.59203E‐24	272.177643 Da	*C*_18_ *H*_24_ *O*_2_	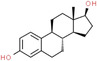
4	Retinoic acid CTD 00006918	1.08099E‐23	300.208923 Da	*C*_20_ *H*_28_ *O*_2_	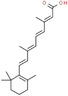
5	Medroxyprogesterone acetate CTD 00006623	2.17989E‐23	386.245697 Da	*C*_24_ *H*_34_ *O*_4_	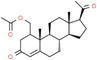
6	Cytarabine CTD 00005743	6.18736E‐23	243.085526 Da	*C*_9_ *H*_13_ *N*_3_ *O*_5_	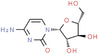
7	Trichostatin A CTD 00000660	5.86427E‐22	302.163055 Da	*C*_17_ *H*_22_ *N*_2_ *O*_3_	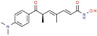
8	Tetradioxin CTD 00006848	1.79885E‐17	319.896545 Da	*C*_12_ *H*_4_ *Cl*_4_ *O*_2_	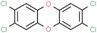
9	Butanoate CTD 00005796	6.64604E‐17	87.045151 Da	*C*_4_ *H*_7_ *O*_2_	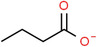
10	Valproic acid CTD 00006977	8.80745E‐17	144.115036 Da	*C*_8_ *H*_16_ *O*_2_	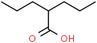

**Figure 18:**
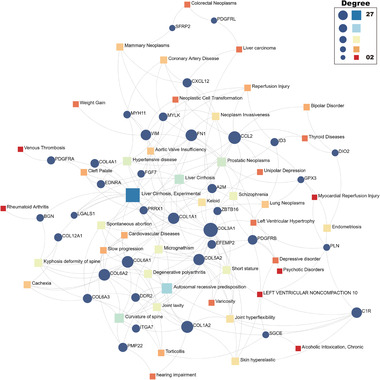
The diseases‐DEGs interaction network.The square nodes are diseases, and circle nodes represent genes. The degree refers to the number of edges that are connected to the specified node in the graph. The greater the degree of the node, the greater the scale.

Finally, to discover diseases associated with the DEGs and any chronic complications, we used the DisGeNET database^[^ ^46^ ^]^ on the NetworkAnalyst platform. The gene‐disease network was set to minimum network and the network nodes with betweenness less than 100 were filtered out to remove the low‐quality diseases. From **Figure** **18**, we find that the network contains 36 genes and 44 diseases. We note that many oncological diseases are revealed including colorectal neoplasms, mammary neoplasms, liver carcinoma and lung neoplasms. Some psychiatric disorders were also associated with the DEGs, for example, bipolar disorder, unipolar depression, schizophrenia, depressive disorder, and psychotic disorder. There are also a number of diseases that affect a person's daily life, such as venous thrombosis, hypertensive disease, liver cirrhosis, rheumatoid arthritis, cardiovascular disease, kyphosis deformity of the spine, micrognathism, joint laxity and hearing impairment.

## 4 Conclusion

In this paper, we present a DEPF to identify and explain single‐cell heterogeneity. DEPF integrates unsupervised dimensionality reduction, ensemble clustering and a bi‐objective fruit fly optimization algorithm. In particular, to guide the optimization, we designed an indicator to determine the direction of optimization of the bi‐objective function. In addition, a bi‐objective fruit fly optimization algorithm is proposed to iteratively search for the optimal clustering results. To demonstrate the effectiveness of DEPF, we applied it to 28 real scRNA‐seq datasets and one large real scRNA‐seq dataset. The experimental results show that DEPF outperforms the ten single‐cell clustering algorithms, six deep clustering algorithms and nine ensemble clustering algorithms in terms of NMI and ARI. The biological interpretability and the transcriptional and post‐transcriptional regulators demonstrate that DEPF can discover biologically meaningful patterns.

## 5 Experimental Section

### Data Collection

The 28 real scRNA‐seq datasets was described and one large‐scale real scRNA‐seq data collected to validate DEPF's ability to identify and explain single‐cell molecular heterogeneity in **Table** **3**. As seen in the Table, the sample sizes of the 28 real scRNA‐seq datasets range from 90 to 72914, and the size of the large‐scale real scRNA‐seq data exceeded 100k. For platform, the 28 real scRNA‐seq datasets came from nine platforms, including the SMARTer platform (7 datasets: Pollen, Wang, Darmanis, Camp(brain), Kolodziejczyk, Xin, Romanov), Smart‐Seq 1/2 platform (6 datasets: Goolam, Deng, Patel, Segerstolpe, Puram, Montoro), inDrop platform (5 datasets: Baron (mouse), Klein, Baron (human), Zilionis, Hrvatin), 10X Genomics platform (three datasets: Slyper, Tabula Muris, Karagiannis), the Drop‐seq platform (two datasets. Chen, Macosko), STRT‐Seq platform (two datasets: Usoskin, Zeisel), Tang platform (one dataset: Yan), Fluidigm C1 platform (1 dataset: Lake) and the CEL‐Seq2 platform (one dataset: Muraro). The large‐scale real scRNA‐seq data, the Orozco dataset, comes from the 10X genomics platform. For tissues, the datasets relate to human (17 datasets) and mouse (12 datasets). There were seven human tissues, including pancreas, brain, tissue, blood, embryo, eye and lung and seven mouse tissues, including brain, embryo, embryonic stem cells, pancreas, retina, tissue, and visual cortex.

**Table 3:** Information on the 28 real scRNA‐seq datasets and one large‐scale real scRNA‐seq dataset

No.	Dataset	Sample	Platform	Tissue
1	Yan^[^ ^47^ ^]^	90	Tang	Human embryo
2	Goolam^[^ ^48^ ^]^	124	Smart‐Seq2	Mouse embryo
3	Deng^[^ ^49^ ^]^	268	Smart‐Seq2	Mouse embryo
4	Pollen^[^ ^50^ ^]^	301	SMARTer	Human tissues
5	Patel^[^ ^51^ ^]^	430	Smart‐Seq	Human tissues
6	Wang^[^ ^52^ ^]^	457	SMARTer	Human pancreas
7	Darmanis^[^ ^53^ ^]^	466	SMARTer	Human brain
8	Camp(brain)^[^ ^54^ ^]^	553	SMARTer	Human brain
9	Usoskin^[^ ^55^ ^]^	622	STRT‐Seq	Mouse brain
10	Kolodziejczyk^[^ ^56^ ^]^	704	SMARTer	Mouse embryo stem cells
11	Xin^[^ ^57^ ^]^	1600	SMARTer	Human pancreas
12	Baron(mouse)^[^ ^58^ ^]^	1886	inDrop	Mouse pancreas
13	Muraro^[^ ^59^ ^]^	2126	CEL‐Seq2	Human pancreas
14	Segerstolpe^[^ ^60^ ^]^	2209	Smart‐Seq2	Human pancreas
15	Klein^[^ ^61^ ^]^	2717	inDrop	Mouse embryo stem cells
16	Romanov^[^ ^62^ ^]^	2881	SMARTer	Mouse brain
17	Zeisel^[^ ^63^ ^]^	3005	STRT‐Seq	Mouse brain
18	Lake^[^ ^64^ ^]^	3042	Fluidigm C1	Human brain
19	Puram^[^ ^29^ ^]^	5902	Smart‐Seq2	Human tissues
20	Montoro^[^ ^65^ ^]^	7193	Smart‐Seq2	Human pancreas
21	Baron(human)^[^ ^58^ ^]^	8569	inDrop	Human pancreas
22	Chen^[^ ^66^ ^]^	12089	Drop‐seq	Mouse brain
23	Slyper	13316	10X Genomics	Human blood
24	Zilionis^[^ ^67^ ^]^	34558	inDrop	Human lung
25	Macosko^[^ ^68^ ^]^	44808	Drop‐seq	Mouse retina
26	Hrvatin^[^ ^69^ ^]^	48266	inDrop	Mouse visual cortex
27	Tabula Muris^[^ ^70^ ^]^	54439	10X Genomics	Mouse tissues
28	Karagiannis^[^ ^71^ ^]^	72914	10X Genomics	Human blood
29	Orozco^[^ ^72^ ^]^	100055	10X Genomics	Human eye

### Unsupervised Dimensionality Reduction

To learn the essential latent representations of the scRNA‐seq data, a hierarchical autoencoder was employed to project the data onto a low‐dimensional space (compressed), which has now been demonstrated to efficiently cluster single‐cell data. In particular, the hierarchical autoencoder^[^ ^17^ ^]^ can detach noise from the normalized data, as well as map the non‐noise data to multiple low‐dimensional latent spaces, where the normalized data was converted from the original data to eliminate the effect of RNA‐sequencing technology, as follows:

(1) $$\begin{array}{c} M_{i,j}=\frac{X_{i,j}-\min\left(X_{i,\cdot }\right)}{\max\left(X_{i,\cdot }\right)-\min\left(X_{i,\cdot }\right)} \end{array}$$

where *X* was the original matrix, *M* was the normalized matrix rescaled from 0 to 1, min (· ) returns the smallest element of the array, max (· ) returns the largest element of the array, and *i*, *j* represent cells and genes, respectively. After that, the first pipeline was a non‐negative kernel autoencoder consisting of an encoder and a decoder that eliminate genes or components that do not contribute significantly to the part‐based representation of scRNA‐seq as follows:

(2) $$\begin{array}{c} e=M_{i,\cdot }W_{E}+b_{E} \end{array}$$

(3) $$\begin{array}{c} \bar{M_{i,\cdot }}=eW_{D}+b_{D} \end{array}$$

where *W*_*E*_ and *W*_*D*_ were the two non‐negative weight matrices and *b*_*E*_ and *b*_*D*_ were the two non‐negative bias vectors. The encoder consisting of *W*_*E*_ and *b*_*E*_ maps the normalized matrix to a low‐dimensional space. Accordingly, *W*_*D*_ and *b*_*D*_ in the decoder were reconstructed from the encoder space. Once the whole process was completed, a non‐noise matrix *A* was generated by deleting the low‐weight genes (noise) from the cells according to the *W*_*E*_ in the encoder.

In addition, a stacked Bayesian autoencoder was the second pipeline in the hierarchical autoencoder to perform the dimension reduction as below:

(4) $$\begin{array}{c} e=f_{E}\left(A\right) \end{array}$$

(5) $$\begin{array}{c} \mu =f_{\mu }\left(e\right) \end{array}$$

(6) $$\begin{array}{c} \sigma =f_{\sigma }\left(\mu \right) \end{array}$$

(7) $$z∼\;N(\mu ,\sigma^{2})$$

(8) $$\begin{array}{c} \bar{A}=f_{D}\left(z\right) \end{array}$$

(9) $$\begin{array}{c} Z=\left\{z_{1},z_{2},\text{…},z_{n}\right\} \end{array}$$

where *f*_*E*_ was the standard encoder, *f*_*D*_ was the standard decoder, *f*_μ_ and *f*_σ_ were the transformation functions in the stacked Bayesian autoencoder to create the distribution *N* and *z* was latent place sampled from *N*. After unsupervised dimensionality reduction, the basic clustering algorithm was used to cluster multiple compressed data *z* to yield various base clustering for cell‐type heterogeneity analysis.

### Basic Clustering Algorithm

To improve clustering performance, an effective basic clustering algorithm should be employed to address the non‐linear embedding in the subspace to produce multiple underlying cluster results to generate cluster ensembles. In this study, it was proposed to use spectral clustering as the basic clustering algorithm, which has been successfully applied in many fields. Nonetheless, as the amount of single‐cell RNA‐seq data increases, spectral clustering, which requires the calculation of cell‐cell similarity, becomes extremely time‐consuming, making it challenging for most algorithms to choose spectral clustering as the base clustering for ensemble clustering. Therefore, an ultra‐scalable spectral clustering^[^ ^73^ ^]^ with approximately linear time and space complexity was employed to cluster the multiple non‐linear embeddings produced by the unsupervised dimensionality reduction to provide a fast clustering and memory usage. In particular, the algorithm first extracts *p* representatives from *n* cells by balancing a mixture of K‐means‐based selection and random selection of representatives. Then, the sparse affinity sub‐matrix *S* between *n* cells and *p* representatives can be calculated as follows:

(10) $$\begin{array}{c} S={\left\{s_{ij}\right\}}_{n\times p} \end{array}$$

(11) $$\begin{array}{c} s_{ij}=\left\{\begin{array}{cc} exp\left(-\frac{{\left\|x_{i}-r_{j}\right\|}^{2}}{2\sigma^{2}}\right), & \;\text{if}\;r_{j}\in Knearest\left(x_{i}\right)\\ 0, & \;\text{otherwise}\; \end{array}\right. \end{array}$$

where *x*_*i*_ was *i*‐th cell, *r*_*j*_ was *j*‐th representative, *Knearest*(· ) was a set of *K* nearest representatives, σ was Gaussian kernel parameter and ‖ · ‖ computes the Euclidean distance. On this basis, it interprets the *S* as a bipartite graph and stacks a (*n* + *p*) × *m* matrix where *m* was related to the eigenvectors. Finally, the clustering results were calculated based on the stacked matrix with k‐means clustering.

### Objective Function

To guide the evolution, suitable objective functions should be devised. In this study, a bi‐objective function with an optimization direction was designed to guide the optimization of the objectives of the proposed algorithm to reduce the impact of low‐quality basic clusters on the consensus clustering algorithm. It has been observed that cell‐to‐cell and cell‐to‐center relationships were important bases for the iterative optimization of the proposed algorithm. Therefore, *Compactness* (*Cp*)^[^ ^74^ ^]^ and *Deviation* (*Dev*)^[^ ^75^ ^]^ were chosen as the objective functions. *Cp* computes the average distance between cells within the same cluster as follows:

(12) $$\begin{array}{c} Cp=\frac{1}{N}\sum_{k=1}^{K}m_{k}\left(\frac{\sum_{x_{i},x_{j}\in c_{k}}dist\left(x_{i},x_{j}\right)}{\frac{m_{k}\left(m_{k}-1\right)}{2}}\right) \end{array}$$

where *N* was the number of cells, *K* was the number of clusters, *m*_*k*_ was the number of cells in *k*‐th cluster, *dist*(· ) computes the Euclidean distance, *c*_*k*_ denotes the *k*‐th cluster, *x*_*i*_ and *x*_*j*_ represent cells. *Dev* sums the distance of the cell to the corresponding cluster center as follows:

(13) $$\begin{array}{c} Dev=\sum_{c_{k}\in C}\sum_{x_{i}\in c_{k}}dist\left(x_{i},c_{k}\right) \end{array}$$

where *C* was the cluster center set, *c*_*k*_ was the *k*‐th cluster center, *x*_*i*_ was the *i*‐th cell. Typically, the closer the cells were to each other and the closer the cells were to the center of the corresponding cluster, the greater the probability that they belong to the same cluster. Following this, it seems reasonable that the optimization direction of the objective function was the same as the default settings of *Cp* and *Dev*. However, due to the complex distribution of scRNA‐seq data from different platforms and species, the direction of association between the data and the true classes may not coincide with the direction of optimization of the objective function, allowing the algorithm to select low‐quality basic clusters, leading to worse consensus clustering results. Therefore, an indicator based on the silhouette coefficient^[^ ^76^ ^]^ was proposed for the adaptive selection of the optimization direction. The indicator adopts the silhouette coefficient (*SC*) to evaluate the relationship between each cell and the entire scRNA‐seq data on the basis of the initial clustering, as follows:

(14) $$\begin{array}{c} SC\left(x_{i}\right)=\left\{\begin{array}{cc} 1-a\left(x_{i}\right)/b\left(x_{i}\right) & \;\text{if}\;a\left(x_{i}\right)<b\left(x_{i}\right)\\ 0 & \;\text{if}\;a\left(x_{i}\right)=b\left(x_{i}\right)\\ b\left(x_{i}\right)/a\left(x_{i}\right)-1 & \;\text{if}\;a\left(x_{i}\right)>b\left(x_{i}\right) \end{array}\right. \end{array}$$

where *x*_*i*_ was the *i*‐th cell, *a*(· ) calculates the average distance of *x*_*i*_ to other cells in the same cluster, *b*(· ) calculates the average distance of *x*_*i*_ to all cells in the neighboring cluster,−1 ⩽ *SC*(*x*_*i*_) ⩽ 1. If *SC*(*x*_*i*_) was closer to 1, it means that *x*_*i*_ was more closely connected to the cells of the same cluster. In contrast, ‐1 indicates that *x*_*i*_ may not belong to the cluster separated by the initial clustering. The positive and negative characteristics of the silhouette coefficient were highly compatible with the optimization direction. If the silhouette coefficient of all cells was greater than a predetermined ϵ, the optimization direction was set to be positive. As long as the silhouette coefficient of a cell was less than the ϵ, the optimization direction was set to negative. On this basis, the two objective functions *f* can be formulated as follows:

(15) $$\begin{array}{c} f=\left\{\begin{array}{cc} +[\mathrm{Dev},\mathrm{Cp}] & s.t.\;\forall \;\mathrm{SC}\left(x_{i}\right)\in \left[\varepsilon ,1\right]\\ -[\mathrm{Dev},\mathrm{Cp}] & s.t.\;\exists \;\mathrm{SC}\left(x_{i}\right)\in \left[-1,\varepsilon \right) \end{array}\right. \end{array}$$

### Dynamic Ensemble Pruning

To optimize iteratively the bi‐objective function, the FOA was first employed.^[^ ^18^ ^]^ The original FOA was representative of a nature‐inspired biological algorithm that addresses the optimization problem by simulating a swarm of fruit flies foraging for food. In this algorithm, fruit flies first apply olfactory to explore and approach the source. After that, fruit flies rely on more sensitive vision to identify and fly toward food, where food represents the optimal solution. The original FOA has several advantages, such as the briefness in the calculation process, the convenience of converting the creature concept into computer code, and the simplicity in structure. However, the original FOA exhibits population diversity reduction and was stuck in a local dilemma facing the single‐cell optimization problem. In order to overcome the above problems and retain the advantages of the original FOA, a bi‐objective fruit fly optimization algorithm (BOFOA) was proposed that consists of four important parts: multiple sub‐population initialization, olfactory‐based equilibrium search, collaborative vision‐based search and consensus clustering algorithm.

### Multiple Sub‐Population Initialization

To enhance the fruit flies' diversity, the population *P* = {*p*_1_, *p*_2_, …, *p*_*D*_} was divided into *D* sub‐populations *p*. Further, the *D* sub‐populations were assigned to the *D* latent spaces separately. Following that, each sub‐population $p_{i}=\left\{{\left(X,Y\right)}_{i}^{1},{\left(X,Y\right)}_{i}^{2},\text{…},{\left(X,Y\right)}_{i}^{G}\right\},i=\left\{1,2,\text{…}D\right\}$ with *G* individuals was constructed, where (*X*, *Y*) denotes the coordinates of each individual in the latent space. Each individual ${\left(X,Y\right)}_{i}^{j}=\left\{{\left(X^{1},Y^{1}\right)}_{i}^{j},{\left(X^{2},Y^{2}\right)}_{i}^{j},\text{…},{\left(X^{M},Y^{M}\right)}_{i}^{j}\right\},i=\left\{1,2,\text{…},D\right\},j=\left\{1,2,\text{…},G\right\}$ was generated in the same way as the original FOA, as follows:

(16) $$\begin{array}{c} X^{m}=X_{min}^{m}+rand\left(-1,1\right)\cdot \left(X_{max}^{m}-X_{min}^{m}\right) \end{array}$$

(17) $$\begin{array}{c} Y^{m}=Y_{min}^{m}+rand\left(-1,1\right)\cdot \left(Y_{max}^{m}-Y_{min}^{m}\right) \end{array}$$

(18) $$\begin{array}{c} S^{m}=\frac{1}{\sqrt{{\left(X^{m}\right)}^{2}+{\left(Y^{m}\right)}^{2}}+\varepsilon } \end{array}$$

(19) $$\begin{array}{c} S^{m}=\left\{\begin{array}{cc} 1 & \;\text{if}\;S^{m}>0.5\\ 0 & \;\text{if}\;S^{m}<0.5 \end{array}\right. \end{array}$$

where *m* ∈ (1, 2, …, *M*), $X_{min}=\left\{X_{min}^{1},X_{min}^{2},\text{…},X_{min}^{M}\right\}$, and $Y_{min}=\left\{Y_{min}^{1},Y_{min}^{2},\text{…},Y_{min}^{M}\right\}$ were the lower bounds while $X_{max}=\left\{X_{max}^{1},X_{max}^{2},\text{…},X_{max}^{M}\right\}$ and $Y_{max}=\left\{Y_{max}^{1},Y_{max}^{2},\text{…},Y_{max}^{M}\right\}$ were the upper bounds, *rand*(− 1, 1) represents a random number that was uniformly distributed between ‐1 and 1, ε was set to ensure that there was a solution. The individual coordinate (*X*, *Y*) was converted to flavor concentration *S* by the formula ((16), (17), (18): the olfactory‐based search in the original FOA). Then *S* was discretized by Equation (19). *S* ^*m*^ = 1 means that the basic clustering π^*m*^ ∈ Π was selected. On the contrary, the *m*‐th basic clustering was dropped.

### Olfactory‐Based Equilibrium Search

After the initialization was completed, each sub‐population performs the olfactory‐based equilibrium search in its own latent space. It was worth noting that the olfactory‐based search in the original FOA was $rand\left(-1,1\right)\cdot \left(X_{max}^{m}-X_{min}^{m}\right)$. This approach, although effective for individual initialization, can leave the population in a local dilemma (i.e., weak exploration but strong exploitation) in subsequent iterations. Therefore, a balancing factor ω was designed to ensure that sub‐populations were balanced between exploration and exploitation during the olfactory‐based equilibrium search, as follows:

(20) $$\begin{array}{c} \omega =e^{\frac{FEs-1}{MaxFEs}\times ln\left(\alpha \right)} \end{array}$$

where *MaxFEs* was the maximum number of iterations, *FEs* was the current number of iteration, and α was a negative manage factor controlling the rate of change of ω. As the iterations of *FEs* close to *MaxFEs*, ω gradually increases, which means that the individual explores the entire solution space at the beginning of the iteration and finds the approximate region of the optimal solution. The individual then uses this region to exploit the final solution at the end of the iteration. Based on ω, the new individual coordinate (*X*, *Y*)_*new*_ was generated as follows:

(21) $$\begin{array}{c} X_{new}^{m}=X_{best}^{m}+e^{\frac{FEs-1}{MaxFEs}\times ln\left(\alpha \right)}\cdot rand\left(-1,1\right) \end{array}$$

(22) $$\begin{array}{c} Y_{new}^{m}=Y_{best}^{m}+e^{\frac{FEs-1}{MaxFEs}\times ln\left(\alpha \right)}\cdot rand\left(-1,1\right) \end{array}$$

where $X_{best}^{m}$ and $Y_{best}^{m}$ represent the coordinate of the individual with optimal bi‐objective value in current sub‐population. The bi‐objective value was obtained by evaluating the binary flavor concentration using bi‐objective function. The optimum was then identified by comparison and the corresponding individual coordinates were saved as (*X*, *Y*)_*best*_, where refer to the section on collaborative vision‐based search on how to compare. After that, the new individual was transformed into a binary flavor concentration *S* by Equations ((18), (19)).

### Collaborative Vision‐Based Search

Next, in the collaborative vision‐based search, the binary flavor concentration *S* (new individual) in each sub‐population was evaluated and information was exchanged between sub‐populations. Since the bi‐objective function consists of two functions, there were two evaluation solutions for *S*. Following this, the best new individual in each sub‐population was then chosen to replace the current best individual in each sub‐population, if the two generated evaluation solutions were better. After the evaluation was completed, each sub‐population contains an optimal individual. Since the search‐spaces for sub‐populations were different representatives of the original scRNA‐seq data space, making the evaluated solutions between sub‐populations asymmetric, the optimal solutions of each sub‐population was combined into a dominant solution set. Further, in order to improve the population diversity to ensure that individuals search for the optimal solution more efficiently in next iteration, the optimal individuals among the different sub‐populations share information, that is, the optimal individuals of the first sub‐population exchange the search latent space with the optimal individuals of the second sub‐population, the second optimal individuals exchange the search latent space with the optimal individuals of the third sub‐population, and the optimal individuals of the last sub‐population exchange the search latent space with the optimal individuals of the first sub‐population.

### Consensus Clustering Algorithm

After completing the optimal location search, the basic clusterings in the ensemble were selected according to the optimal binary flavor concentration *S*. Next, a consensus clustering algorithm was used to generate the final clustering results based on the pruned ensemble. To efficiently compute consensus clustering with sufficient information from the clustering ensemble, DEPF employs the locally‐weighted ensemble clustering^[^ ^77^ ^]^ as the consensus clustering algorithm. The implementation of the consensus clustering algorithm has four steps: (i) evaluating the uncertainty of each cluster *c*_*i*_ in the ensemble $\Pi^{z_{d}}=\left\{\pi_{1},\text{…}\pi_{n}\right\}$ was as follows:

(23) $$\begin{array}{c} U\left(c_{i}\right)=-\sum_{d=1}^{D}\sum_{n=1}^{N^{d}}\sum_{j=1}^{k^{n}}\frac{\left|c_{i}\cap c_{j}^{n}\right|}{\left|c_{i}\right|}log_{2}\frac{\left|c_{i}\cap c_{j}^{n}\right|}{\left|c_{i}\right|} \end{array}$$

where *D* was the number of subspaces in *Z*, *N*_*d*_ was the number of basic clustering in subspace *z*_*d*_, *k* ^*n*^ denotes the number of clusters in the basic clustering π_*n*_, ∩ represents the intersection of two clusters, | · | counts the number of cells, (ii) identifying the reliability of the uncertainty of each cluster $ECI\left(c_{i}\right)=e^{-\frac{U(c_{i})}{\theta \cdot D\cdot N}}$ where θ was an adjustment factor, (iii) constructing a co‐association matrix using *ECI* as the weight of each cluster and a consensus function on the basis of hierarchical agglomerative clustering, (iv) output the final clustering results so that the consensus function calculates the co‐association matrix.

### Evaluation Metrics

To evaluate the quality of the cell partitions in DEPF, the ARI^[^ ^78^ ^]^ and NMI^[^ ^79^ ^]^ were adopted, both of which rely on the ground truth labels. The NMI normalizes the mutual information^[^ ^80^ ^]^ between the actual partition *T* and the predicted partition *P* in the following manner:

(24) $$\begin{array}{c} NMI\left(T,P\right)=\frac{I(T,P)}{\sqrt{H(T)+H(P)}} \end{array}$$

where *I*(· ) denotes the Mutual Information between the inputs, *H*(· ) was the entropy of the partitioning and *NMI*(· ) ∈ [0, 1]. *NMI*(· ) = 0 means that there was no correlation between prediction and truth. Contrary to this, the closer the value of *NMI*(· ) was to 1, the closer the prediction was to the truth. ARI was the corrected‐for‐chance of the Rand Index^[^ ^81^ ^]^ as follows:

(25) ARI(T,P)=∑i,jnij2−∑ini2∑jnj2/n212∑ini2+∑jnj2−∑ini2∑jnj2/n2

where *n* was the number of all cells in the scRNA‐seq data, *n*_*ij*_ was the number of cells belonging to both the *i*‐th sub‐partition in *T* and the *j*‐th sub‐partition in *P*, *n*_*i*_ was the number of cells in *i*‐th sub‐partition of *T*, *n*_*j*_ was the number of cells in *j*‐th sub‐partition of *P* and *ARI*(· ) ∈ [ − 1, 1]. Approximating to the NMI, a larger value of *ARI*(· ) indicates a better performance of the clustering algorithm.

### Implementation Details

In DEPF, the ϵ for the direction of optimization of the objective function was set to ‐0.6. The influence of the ϵ was discussed in the Section '2.8 Impact of the bi‐objective function with the optimization direction.' The value of α (negative manage factor) was set to 13. The details about determining the value of α were provided to Section S8 (Supporting Information). In addition, the number of iterations was set to 20 and the population size was set to 9. The figures illustrating the optimization process and validation were provided in Section S9 (Supporting Information), including the line plots for the two objective function *Cp* and *Dev* changes with the iterations. To prevent lucky breaks, BOFOA was run 50 times independently on each scRNA‐seq data. The 50 results were then evaluated using the ARI and NMI and the average values calculated. Section S9 (Supporting Information) shows the boxplots of ARI and NMI values for all 50 BOFOA runs. The parameters of the unsupervised dimensionality reduction, the basic clustering algorithm and the consensus clustering algorithm were the same as in the original papers. In addition, the basic clustering algorithm was run 10 times in each subspace to ensure that there was as much diversity as possible in the basic clusters of the set.

### Baseline Methods

To demonstrate the effectiveness of the proposed model, three categories of methods were compared including single‐cell clustering algorithms, deep clustering algorithms, and ensemble clustering algorithms, as shown in **Table** **4**. From Table 4, the single‐cell clustering algorithms generally consist of traditional dimensionality‐reduction methods and basic clustering algorithms; for instance, SC3, Seurat, SCANPY, and CIDR all use PCA to do unsupervised dimensionality reduction. The K‐means, graph‐based clustering and hierarchical‐based clustering were employed as basic clustering algorithms. Deep clustering algorithms involving GraphSCC, scziDesk, scDCC, DCA, DEC and scGAE contain a wide variety of neural networks that include graph convolutional networks, autoencoders based on the ZINB model, deep networks and graph‐based encoders. The ensemble clustering algorithms, including LWEA, U‐SENC, ECC, ECPCS‐MC, KCC, LWGP, MCLA, PTGP and SEC were characterized by different consensus functions.

**Table 4:** Description of the nine single‐cell clustering algorithms, six deep clustering algorithms and nine ensemble clustering algorithms

Category	Name	Method type	Link
single‐cell clustering algorithm	scDHA^[^ ^17^ ^]^	hierarchical autoencoder	https://bioinformatics.cse.unr.edu/software/scDHA/
	SC3^[^ ^7^ ^]^	PCA + k‐means	http://bioconductor.org/packages/SC3
	Seurat^[^ ^9^ ^]^	PCA + graph‐based clustering	https://github.com/satijalab/seurat/
	SCANPY^[^ ^10^ ^]^	PCA + graph‐based clustering	https://anaconda.org/bioconda/scanpy
	CIDR^[^ ^8^ ^]^	PCA + hierarchical‐based clustering	https://github.com/VCCRI/CIDR
	SINCERA^[^ ^6^ ^]^	hierarchical clustering	https://github.com/xu‐lab/SINCERA
	SHARP^[^ ^12^ ^]^	weighted ensemble clustering	https://github.com/shibiaowan/SHARP
	SAME‐clustering^[^ ^13^ ^]^	five agents + expectation maximization algorithm	https://github.com/yycunc/SAMEclustering
	SAFE‐clustering^[^ ^11^ ^]^	four agents + three hypergraph‐based partitioning algorithms	https://github.com/yycunc/SAFEclustering
deep clustering algorithm	GraphSCC^[^ ^82^ ^]^	graph convolutional network	https://github.com/GeniusYx/GraphSCC
	scziDesk^[^ ^83^ ^]^	ZINB model‐based autoencoder	https://github.com/xuebaliang/scziDesk
	scDCC^[^ ^16^ ^]^	ZINB model‐based autoencoder	https://github.com/ttgump/scDCC
	DCA^[^ ^84^ ^]^	ZINB model‐based autoencoder	https://github.com/theislab/dca
	DEC^[^ ^85^ ^]^	deep neural network	https://github.com/XifengGuo/DEC‐keras
	scGAE^[^ ^15^ ^]^	graph‐based encoder	https://github.com/ZixiangLuo1161/scGAE
ensemble clustering algorithm	LWEA^[^ ^77^ ^]^	hierarchical agglomerative clustering	https://www.researchgate.net/publication/316681928
	U‐SENC^[^ ^73^ ^]^	bipartite graph + K‐means	https://www.researchgate.net/publication/330760669
	ECC^[^ ^86^ ^]^	entroy‐based utility function + K‐means	http://scholar.harvard.edu/yyl/ecc
	ECPCS‐MC^[^ ^87^ ^]^	meta‐clustering	www.researchgate.net/publication/328581758
	KCC^[^ ^88^ ^]^	utility functions + K‐means	http://hongfuliu.com/
	LWGP^[^ ^77^ ^]^	bipartite graph	https://www.researchgate.net/publication/316681928
	MCLA^[^ ^89^ ^]^	meta‐clustering	http://strehl.com/soft.html
	PTGP^[^ ^90^ ^]^	bipartite graph	https://www.researchgate.net/publication/284259332
	SEC^[^ ^91^ ^]^	spectral‐based clustering	https://github.com/Li‐Hongmin/

## Conflict of Interest

The authors declare no conflict of interest.

## Author Contributions

Y.F. and X.L. conceived the study. Y.W., F.W., H.L., K.W., and Y.Y. helped to test the methods and reproduce the analyses. Y.F. and X.L. wrote the manuscript, and all authors revised it. X.L. supervised the project. All authors read and approved the final version of the manuscript.

## Supporting information

Supporting Information

## Data Availability

The data that support the findings of this study are available in the supplementary material of this article.
