# Supplementary material for: Reliable Identification and Interpretation of Single‐Cell Molecular Heterogeneity and Transcriptional Regulation using Dynamic Ensemble Pruning
Source: Adv Sci (Weinh). 2023 Jun 8;10(22):2205442. doi: 10.1002/advs.202205442 (PMC10401140; doi:10.1002/advs.202205442)
Supplement: Supplementary file 1 — Supporting Information [file ADVS-10-2205442-s001.pdf]

ADVANCED SCIENCE
Open Access

Supporting Information

for *Adv. Sci.*, DOI 10.1002/advs.202205442

Reliable Identification and Interpretation of Single-Cell Molecular Heterogeneity and Transcriptional Regulation using Dynamic Ensemble Pruning

*Yi Fan, Yunhe Wang, Fuzhou Wang, Lei Huang, Yuning Yang, Ka-Chun Wong and Xiangtao Li**

Reliable Identification and Interpretation of Single-cell Molecular Heterogeneity and Transcriptional Regulation using Dynamic Ensemble Pruning

*Yi Fan Yunhe Wang Fuzhou Wang Lei Huang Yuning Yang Ka-chun Wong Xiangtao Li**

Y. Fan, X. Li
School of Artificial Intelligence
Jilin University
Jilin, China
Email: lixt314@jlu.edu.cn
Y. Wang
School of Artificial Intelligence
Hebei University of Technology
Tianjin, China
F. Wang, L. Huang, K. Wong
Department of Computer science
City University of Hong Kong
Hong Kong SAR
Y. Yang
Donnelly Centre for Cellular and Biomolecular Research
University of Toronto
Toronto, ON, Canada

1 DEPF can provide better performance than several single-cell clustering algorithms

Table S1: The NMI values of DEPF and 10 single-cell clustering algorithms on 28 real scRNA-seq datasets. "NA" values indicate that the method was not able to analyze the dataset (crashed or out-of-memory).

dataname	sample	DEPF	scDHA	k-means	SC3	Seurat	SCANPY	SHARP	CIDR	SINCERA	SAME-clustering	SAFE-clustering
Yan	90	0.89	0.89	0.86	0.8	0.55	0.87	0.71	0.84	0.82	0.72	0.72
Goolam	124	0.89	0.82	0.63	0.8	0.61	0.71	0.84	0.78	0.61	0.76	0.86
Deng	268	0.89	0.89	0.78	0.73	0.53	0.7	0.41	0.74	0.73	0.71	0.69
Pollen	301	0.96	0.96	0.94	0.95	0.8	0.91	0.32	0.94	0.93	0.87	0.94
Patel	430	0.89	0.84	0.83	0.67	0.76	0.72	0.26	0.57	0.67	0.86	0.87
Wang	457	0.88	0.83	0.57	0.81	0.71	0.71	0.6	0.71	0.43	0.5	0.71
Darmanis	466	0.81	0.75	0.62	0.67	0.64	0.69	0.21	0.64	0.66	0.75	0.63
Camp(brain)	553	0.85	0.82	0.55	0.68	0.7	0.69	0.52	0.49	0.62	0.75	0.66
Usoskin	622	0.98	0.81	0.31	0.79	0.74	0.65	0.23	0.8	0.54	0.85	0.81
Kolodziejczyk	704	1	0.9	0.51	0.68	0.68	0.67	0.02	0.57	0.54	0.72	0.68
Xin	1600	0.94	0.87	0.6	0.39	0.6	0.61	0.72	0.55	0.42	0.07	0.44
Baron(mouse)	1886	0.91	0.85	0.59	0.65	0.75	0.74	0.76	0.51	0.61	0.73	0.67
Muraro	2126	0.86	0.88	0.53	0.69	0.77	0.74	0.46	0.43	0.51	0.82	0.75
Segerstolpe	2209	0.89	0.9	0.53	0.65	0.75	0.69	0.44	0.45	0.62	0.77	0.7
Klein	2717	0.97	0.97	0.4	0.69	0.71	0.76	0.73	0.66	0.67	0.86	0.76
Romanov	2881	0.68	0.69	0.35	0.43	0.6	0.58	0.56	0.34	0.31	0.54	0.39
Zeisel	3005	0.86	0.78	0.55	0.62	0.67	0.63	0.6	0.47	0.47	0.73	0.54
Lake	3042	0.77	0.67	0.62	0.68	0.73	0.73	0.35	0.54	0.47	0.60	0.53
Puram	5902	0.86	0.79	0.63	0.45	0.66	0.62	0.68	0.63	0.68	0.61	0.39
Montoro	7193	0.68	0.74	0.56	0.3	0.5	0.47	0.67	0.46	0.24	0.43	0.28
Baron(human)	8569	0.9	0.88	0.63	0.5	0.8	0.77	0.75	0.72	0.46	0.72	0.67
Chen	12089	0.74	0.77	0.63	0.53	0.79	0.77	0.67	0.42	0.53	0.68	0.64
Slyper	13316	0.71	0.73	0.62	0.36	0.6	0.59	0.39	0.7	0.16	0.64	0.55
Zilionis	34558	0.89	0.83	0.62	0.41	0.7	0.66	0.56	0.58	0.08	NA	NA
Macosko	44808	0.62	0.59	0.4	0.31	0.56	0.56	0.46	0.33	0.19	NA	NA
Hrvatin	48266	0.98	0.92	0.88	0.59	0.74	0.77	0.88	NA	NA	NA	NA
TabulaMuris	54439	0.78	0.8	0.68	0.65	0.77	0.77	0.63	NA	NA	NA	NA
Karagiannis	72914	0.68	0.66	0.65	0.49	0.73	0.66	0.43	NA	NA	NA	NA
average NMI	11626.25	0.85	0.82	0.61	0.61	0.68	0.69	0.53	0.59	0.52	0.68	0.65

Table S2: The ARI values of DEPF and 10 single-cell clustering algorithms on 28 real scRNA-seq datasets. "NA" values indicate that the method was not able to analyze the dataset (crashed or out-of-memory).

dataname	sample	DEPF	scDHA	k-means	SC3	Seurat	SCANPY	SHARP	CIDR	SINCERA	SAME-clustering	SAFE-clustering
Yan	90	0.86	0.86	0.8	0.66	0.39	0.84	0.62	0.8	0.72	0.63	0.63
Goolam	124	0.91	0.84	0.48	0.6	0.42	0.42	0.86	0.7	0.3	0.58	0.89
Deng	268	0.89	0.89	0.6	0.44	0.29	0.34	0.23	0.51	0.7	0.51	0.43
Pollen	301	0.94	0.92	0.89	0.96	0.61	0.77	0.09	0.9	0.85	0.74	0.89
Patel	430	0.92	0.87	0.82	0.46	0.76	0.66	0.08	0.45	0.47	0.9	0.9
Wang	457	0.93	0.85	0.44	0.85	0.65	0.58	0.43	0.63	0.29	0.51	0.7
Darmanis	466	0.78	0.68	0.44	0.44	0.58	0.48	0.11	0.5	0.55	0.73	0.43
Camp(brain)	553	0.88	0.86	0.48	0.56	0.65	0.53	0.45	0.34	0.59	0.75	0.59
Usoskin	622	0.99	0.82	0.23	0.8	0.66	0.39	0.09	0.82	0.38	0.88	0.85
Kolodziejczyk	704	1	0.87	0.48	0.44	0.45	0.43	0.04	0.43	0.46	0.54	0.51
Xin	1600	0.98	0.95	0.44	0.14	0.42	0.32	0.77	0.57	0.16	0.02	0.15
Baron(mouse)	1886	0.96	0.88	0.29	0.26	0.49	0.39	0.82	0.47	0.39	0.47	0.4
Muraro	2126	0.91	0.91	0.34	0.38	0.57	0.46	0.31	0.22	0.32	0.72	0.62
Segerstolpe	2209	0.89	0.92	0.29	0.29	0.44	0.31	0.35	0.37	0.4	0.55	0.46
Klein	2717	0.98	0.98	0.29	0.45	0.54	0.62	0.65	0.68	0.61	0.83	0.66
Romanov	2881	0.74	0.75	0.3	0.22	0.39	0.3	0.59	0.32	0.23	0.36	0.13
Zeisel	3005	0.91	0.8	0.36	0.33	0.51	0.32	0.47	0.37	0.42	0.68	0.23
Lake	3042	0.79	0.6	0.38	0.39	0.48	0.43	0.21	0.47	0.31	0.59	0.65
Puram	5902	0.86	0.65	0.44	0.11	0.32	0.24	0.73	0.68	0.71	0.33	0.07
Montoro	7193	0.73	0.81	0.45	0.11	0.24	0.2	0.78	0.3	0.13	0.28	0.02
Baron(human)	8569	0.93	0.93	0.41	0.14	0.58	0.48	0.73	0.73	0.34	0.51	0.4
Chen	12089	0.63	0.78	0.33	0.16	0.63	0.63	0.62	0.36	0.6	0.66	0.53
Slyper	13316	0.62	0.78	0.4	0.07	0.25	0.26	0.36	0.63	0	0.41	0.44
Zilionis	34558	0.89	0.84	0.48	0.11	0.36	0.38	0.41	0.53	0.02	NA	NA
Macosko	44808	0.41	0.73	0.25	0.07	0.22	0.23	0.73	0.17	0.41	NA	NA
Hrvatin	48266	0.97	0.9	0.85	0.26	0.44	0.56	0.89	NA	NA	NA	NA
TabulaMuris	54439	0.66	0.71	0.43	0.3	0.54	0.5	0.46	NA	NA	NA	NA
Karagiannis	72914	0.45	0.54	0.39	0.26	0.42	0.35	0.36	NA	NA	NA	NA
average ARI	11626.25	0.84	0.82	0.46	0.37	0.48	0.44	0.47	0.52	0.41	0.57	0.5

2 DEPF performs better than several deep clustering algorithms

Table S3: The NMI values of DEPF and 6 deep clustering algorithms on 28 real scRNA-seq datasets. ”NA” values indicate that the method was not able to analyze the dataset (crashed or out-of-memory).

Platform	Dataset	DEPF	GraphSCC	scziDesk	scDCC	DCA	DEC	scGAE
	Pollen	0.96	0.96	0.94	0.95	0.91	0.8	0.88
	Wang	0.88	0.72	0.65	0.67	0.71	0.55	0.59
	Darmanis	0.81	0.8	0.73	0.81	0.8	0.68	0.73
SMARTer	Campbrain	0.85	0.57	0.51	0.62	0.67	0.57	0.64
	Kolodziejczyk	1	0.64	0.08	0.78	0.78	0.66	0.78
	Xin	0.94	0.76	0.53	0.57	0.59	0.51	0.53
	Romanov	0.68	0.71	0.7	0.58	0.52	0.39	0.62
	Patel	0.89	0.91	0.66	0.91	0.8	0.77	0.84
	Goolam	0.89	0.91	0.86	0.72	0.72	0.49	0.48
Smart-Seq 1/2	Deng	0.89	0.78	0.71	0.78	0.79	0.67	0.76
	Segerstolpe	0.89	0.87	0.74	0.73	0.69	0.63	0.59
	Puram	0.86	0.76	0.74	0.7	0.73	0.54	0.66
	Montoro	0.68	0.59	0.57	0.55	0.56	0.54	0.59
	Baron(mouse)	0.91	0.81	0.77	0.77	0.7	0.64	0.58
	Klein	0.97	0.96	0.74	0.62	0.73	0.56	0.81
inDrop	Baron(human)	0.9	0.84	0.78	0.76	0.73	0.65	0.66
	Zilionis	0.89	0.77	0.33	0.77	0.72	0.71	0.33
	Hrvatin	0.98	0.92	0.9	0.9	0.88	0.85	0.79
	Slyper	0.71	0.66	0.59	0.58	0.63	0.66	0.51
10X Genomics	Tabula Muris	0.78	0.77	0.74	0.74	0.71	0.75	NA
	Karagiannis	0.68	0.69	0.41	0.64	0.61	0.49	NA
Drop-seq	Chen	0.74	0.73	0.66	0.71	0.65	0.75	0.67
	Macosko	0.62	0.79	0.13	0.61	0.54	0.42	0.44
STRT-Seq	Usoskin	0.98	0.86	0.22	0.72	0.67	0.42	0.62
	Zeisel	0.86	0.75	0.64	0.78	0.71	0.57	0.57
CEL-Seq2	Muraro	0.86	0.87	0.82	0.81	0.83	0.67	0.58
Tang	Yan	0.89	0.86	0.78	0.86	0.86	0.63	0.78
Fluidigm C1	Lake	0.77	0.69	0.64	0.72	0.61	0.57	0.68
average	NMI	0.85	0.78	0.63	0.73	0.71	0.61	0.64

Table S4: The ARI values of DEPF and 6 deep clustering algorithms on 28 real scRNA-seq datasets. ”NA” values indicate that the method was not able to analyze the dataset (crashed or out-of-memory).

Platform	Dataset	DEPF	GraphSCC	scziDesk	scDCC	DCA	DEC	scGAE
	Pollen	0.94	0.96	0.94	0.95	0.85	0.72	0.76
	Wang	0.93	0.73	0.53	0.63	0.65	0.46	0.35
	Darmanis	0.78	0.79	0.72	0.8	0.72	0.55	0.56
SMARTer	Campbrain	0.88	0.54	0.39	0.51	0.67	0.5	0.63
	Kolodziejczyk	1	0.61	0.1	0.72	0.73	0.64	0.72
	Xin	0.98	0.84	0.36	0.4	0.37	0.4	0.26
	Romanov	0.74	0.71	0.7	0.51	0.47	0.29	0.59
	Patel	0.92	0.93	0.63	0.92	0.81	0.78	0.85
	Goolam	0.91	0.91	0.75	0.56	0.54	0.36	0.23
Smart-Seq 1/2	Deng	0.89	0.56	0.51	0.55	0.56	0.44	0.57
	Segerstolpe	0.89	0.9	0.5	0.55	0.42	0.48	0.25
	Puram	0.86	0.61	0.55	0.48	0.5	0.34	0.42
	Montoro	0.73	0.55	0.45	0.48	0.45	0.53	0.65
	Baronmouse	0.96	0.78	0.55	0.58	0.42	0.45	0.23
	Klein	0.98	0.98	0.75	0.48	0.57	0.43	0.82
inDrop	Baron(human)	0.93	0.79	0.6	0.53	0.49	0.49	0.4
	Zilionis	0.89	0.62	0.21	0.65	0.57	0.51	0.24
	Hrvatin	0.97	0.89	0.86	0.9	0.85	0.74	0.7
	Slyper	0.62	0.39	0.31	0.31	0.39	0.41	0.25
10X Genomics	Tabula Muris	0.66	0.58	0.41	0.45	0.37	0.62	NA
	Karagiannis	0.45	0.43	0.19	0.38	0.31	0.33	NA
Drop-seq	Chen	0.63	0.51	0.26	0.38	0.27	0.69	0.27
	Macosko	0.41	0.9	0.04	0.36	0.22	0.23	0.26
STRT-Seq	Usoskin	0.99	0.88	0.17	0.77	0.68	0.35	0.56
	Zeisel	0.91	0.76	0.61	0.78	0.58	0.46	0.41
CEL-Seq2	Muraro	0.91	0.91	0.73	0.69	0.7	0.59	0.26
Tang	Yan	0.86	0.8	0.62	0.81	0.8	0.46	0.59
Fluidigm C1	Lake	0.79	0.5	0.36	0.4	0.37	0.41	0.37
average	ARI	0.84	0.73	0.49	0.59	0.55	0.49	0.47

3 DEPF is superior to several ensemble clustering algorithms

Table S5: The NMI values of DEPF and 9 ensemble clustering algorithms on Human

Tissue	Dataset	DEPF	LWEA	U-SENC	ECC	ECPCS-MC	KCC	LWGP	MCLA	PTGP	SEC
Human blood	Karagiannis	0.68	0.63	0.64	0.6	0.62	0.59	0.62	0.58	0.61	0.57
Human blood	Slyper	0.71	0.63	0.61	0.59	0.64	0.62	0.63	0.59	0.63	0.6
Human brain	Campbrain	0.85	0.63	0.59	0.59	0.63	0.61	0.61	0.59	0.62	0.6
Human brain	Darmanis	0.81	0.62	0.73	0.6	0.61	0.62	0.61	0.62	0.6	0.63
Human brain	Lake	0.77	0.59	0.69	0.57	0.6	0.58	0.6	0.57	0.59	0.58
Human embryo	Yan	0.89	0.79	0.83	0.85	0.81	0.84	0.8	0.81	0.75	0.81
Human lung	Zilionis	0.89	0.62	0.77	0.6	0.62	0.62	0.62	0.63	0.63	0.63
Human pancreas	Baronhuman	0.9	0.72	0.77	0.67	0.7	0.68	0.7	0.66	0.71	0.67
Human pancreas	Montoro	0.68	0.55	0.56	0.51	0.55	0.52	0.55	0.52	0.55	0.5
Human pancreas	Muraro	0.86	0.6	0.8	0.58	0.59	0.57	0.59	0.56	0.6	0.56
Human pancreas	Segerstolpe	0.89	0.61	0.71	0.57	0.59	0.56	0.59	0.55	0.59	0.56
Human pancreas	Wang	0.88	0.6	0.61	0.52	0.5	0.57	0.59	0.55	0.58	0.55
Human pancreas	Xin	0.94	0.63	0.65	0.49	0.64	0.58	0.6	0.59	0.6	0.6
Human tissues	Patel	0.89	0.56	0.7	0.54	0.44	0.54	0.57	0.5	0.57	0.52
Human tissues	Pollen	0.96	0.94	0.91	0.92	0.9	0.91	0.93	0.91	0.87	0.9
Human tissues	Puram	0.86	0.65	0.73	0.64	0.65	0.63	0.65	0.63	0.66	0.64
average NMI		0.84	0.65	0.71	0.62	0.63	0.63	0.64	0.62	0.64	0.62

Table S6: The NMI values of DEPF and 9 ensemble clustering algorithms on Mouse

Tissue	Dataset	DEPF	LWEA	U-SENC	ECC	ECPCS-MC	KCC	LWGP	MCLA	PTGP	SEC
Mouse brain	Chen	0.74	0.67	0.64	0.62	0.66	0.62	0.65	0.62	0.64	0.63
Mouse brain	Romanov	0.68	0.43	0.64	0.41	0.43	0.41	0.41	0.4	0.42	0.42
Mouse brain	Usoskin	0.98	0.32	0.72	0.22	0.3	0.29	0.34	0.3	0.3	0.35
Mouse brain	Zeisel	0.86	0.6	0.78	0.6	0.6	0.6	0.6	0.58	0.61	0.59
Mouse embryo	Deng	0.89	0.82	0.66	0.75	0.79	0.77	0.82	0.75	0.71	0.8
Mouse embryo	Goolam	0.89	0.87	0.81	0.79	0.82	0.84	0.87	0.81	0.87	0.84
Mouse embryo stem cells	Klein	0.97	0.57	0.95	0.43	0.57	0.57	0.57	0.49	0.57	0.55
Mouse embryo stem cells	Kolodziejczyk	1	0.45	0.72	0.44	0.44	0.44	0.45	0.44	0.45	0.45
Mouse pancreas	Baro(nmouse)	0.91	0.57	0.74	0.61	0.57	0.6	0.59	0.57	0.58	0.59
Mouse retina	Macosko	0.62	0.5	0.52	0.46	0.5	0.46	0.49	0.44	0.49	0.45
Mouse tissues	Tabula Muris	0.78	0.72	0.74	0.66	0.7	0.67	0.7	0.66	0.7	0.66
Mouse visual cortex	Hrvatin	0.98	0.89	0.91	0.86	0.9	0.88	0.89	0.89	0.88	0.88
average NMI		0.86	0.62	0.74	0.57	0.61	0.60	0.62	0.58	0.60	0.60

Table S7: The ARI values of DEPF and 9 ensemble clustering algorithms on Human

Tissue	Dataset	DEPF	LWEA	U-SENC	ECC	ECPCS-MC	KCC	LWGP	MCLA	PTGP	SEC
Human blood	Karagiannis	0.45	0.38	0.37	0.35	0.36	0.34	0.34	0.31	0.34	0.31
Human blood	Slyper	0.62	0.39	0.28	0.3	0.41	0.39	0.38	0.31	0.39	0.41
Human brain	Campbrain	0.88	0.65	0.59	0.58	0.65	0.61	0.62	0.57	0.63	0.63
Human brain	Darmanis	0.78	0.49	0.61	0.45	0.48	0.47	0.46	0.48	0.44	0.49
Human brain	Lake	0.79	0.39	0.42	0.34	0.37	0.36	0.37	0.33	0.37	0.38
Human embryo	Yan	0.86	0.68	0.76	0.76	0.73	0.8	0.66	0.68	0.58	0.74
Human lung	Zilionis	0.89	0.47	0.63	0.42	0.47	0.5	0.46	0.49	0.5	0.52
Human pancreas	Baron(human)	0.93	0.56	0.51	0.45	0.52	0.48	0.49	0.42	0.54	0.46
Human pancreas	Montoro	0.73	0.41	0.44	0.38	0.4	0.39	0.41	0.39	0.4	0.39
Human pancreas	Muraro	0.91	0.46	0.61	0.37	0.44	0.39	0.42	0.38	0.45	0.4
Human pancreas	Segerstolpe	0.89	0.41	0.41	0.33	0.42	0.35	0.39	0.31	0.36	0.38
Human pancreas	Wang	0.93	0.48	0.49	0.39	0.41	0.44	0.46	0.46	0.45	0.46
Human pancreas	Xin	0.98	0.49	0.46	0.29	0.52	0.39	0.4	0.4	0.43	0.48
Human tissues	Patel	0.92	0.44	0.72	0.42	0.33	0.42	0.44	0.39	0.44	0.39
Human tissues	Pollen	0.94	0.94	0.84	0.9	0.82	0.87	0.93	0.88	0.81	0.83
Human tissues	Puram	0.86	0.46	0.52	0.44	0.46	0.44	0.45	0.44	0.47	0.43
average ARI		0.84	0.51	0.54	0.45	0.49	0.48	0.48	0.45	0.48	0.48

Table S8: The ARI values of DEPF and 9 ensemble clustering algorithms on Mouse

Tissue	Dataset	DEPF	LWEA	U-SENC	ECC	ECPCS-MC	KCC	LWGP	MCLA	PTGP	SEC
Mouse brain	Chen	0.63	0.49	0.25	0.29	0.4	0.3	0.36	0.25	0.38	0.33
Mouse brain	Romanov	0.74	0.47	0.66	0.36	0.46	0.38	0.37	0.37	0.39	0.45
Mouse brain	Usoskin	0.99	0.25	0.73	0.17	0.22	0.21	0.28	0.24	0.23	0.29
Mouse brain	Zeisel	0.91	0.51	0.69	0.52	0.5	0.51	0.51	0.4	0.52	0.49
Mouse embryo	Deng	0.89	0.8	0.41	0.54	0.79	0.66	0.77	0.7	0.54	0.75
Mouse embryo	Goolam	0.91	0.9	0.83	0.72	0.87	0.89	0.9	0.86	0.9	0.86
Mouse embryo stem cells	Klein	0.98	0.42	0.97	0.32	0.43	0.42	0.43	0.4	0.42	0.42
Mouse embryo stem cells	Kolodziejczyk	1	0.39	0.66	0.38	0.38	0.38	0.4	0.39	0.4	0.39
Mouse pancreas	Baron(mouse)	0.96	0.33	0.52	0.41	0.36	0.35	0.34	0.28	0.33	0.36
Mouse retina	Macosko	0.41	0.3	0.22	0.24	0.29	0.25	0.28	0.17	0.29	0.26
Mouse tissues	Tabula Muris	0.66	0.58	0.39	0.34	0.44	0.39	0.44	0.33	0.46	0.4
Mouse visual cortex	Hrvatin	0.97	0.82	0.88	0.76	0.84	0.81	0.81	0.82	0.8	0.81
average ARI		0.84	0.52	0.60	0.42	0.50	0.46	0.49	0.43	0.47	0.48

4 Evaluations on Large Scale scRNA-seq data >100k

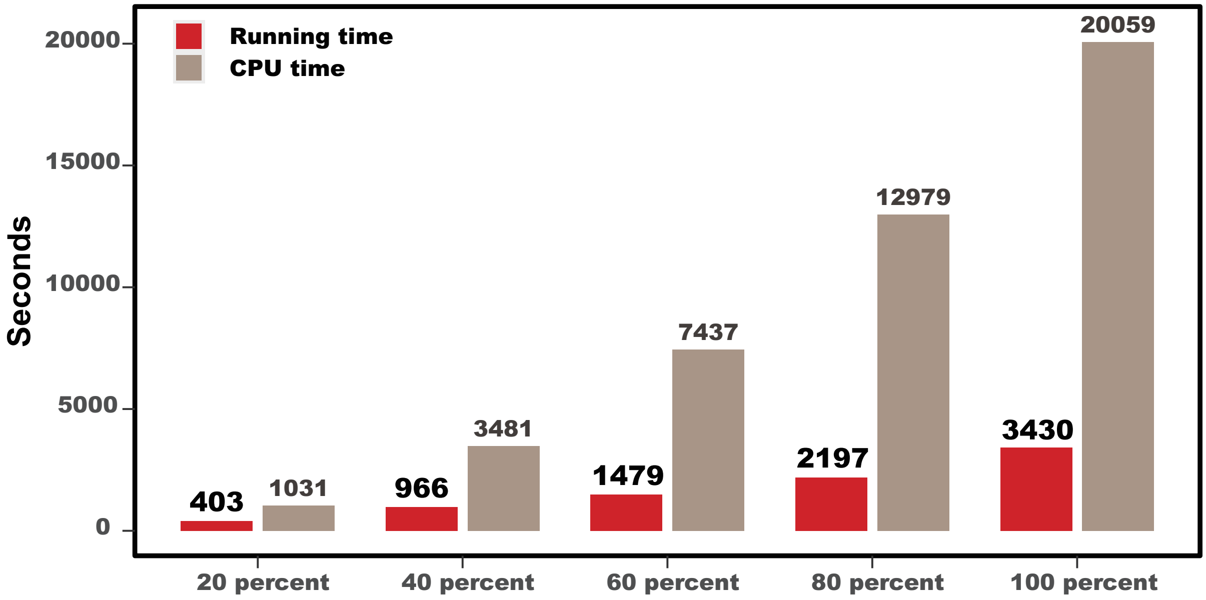

Figure S1: Running time and CPU time of DEPF for sample proportions of 20%, 40%, 60% and 80% on the Orozco dataset.

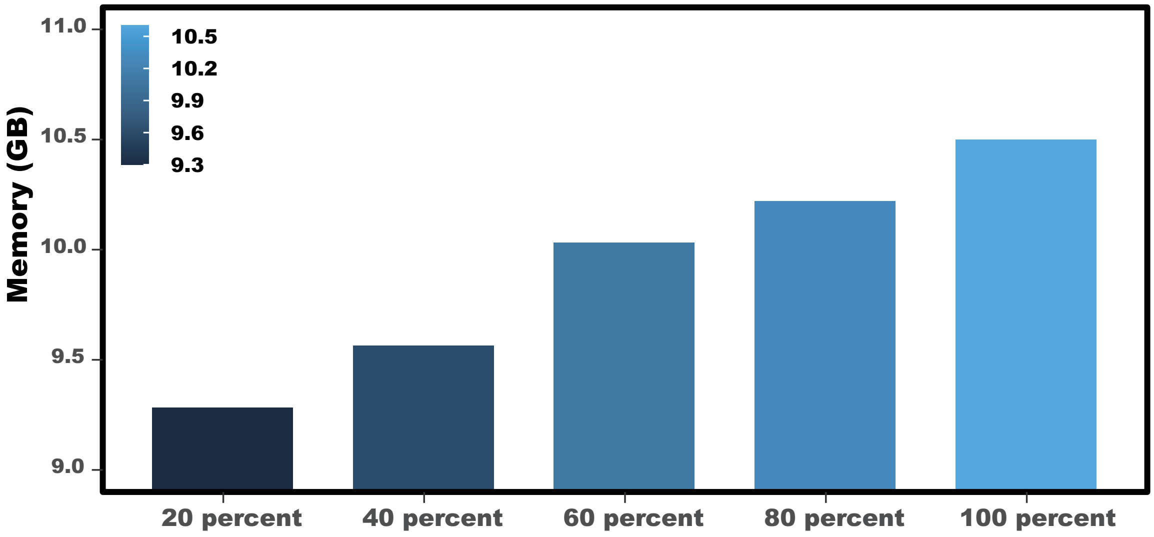

Figure S2: Memory of DEPF on the Orozco dataset for 20 percent, 40 percent, 60 percent and 80 percent sample proportions.

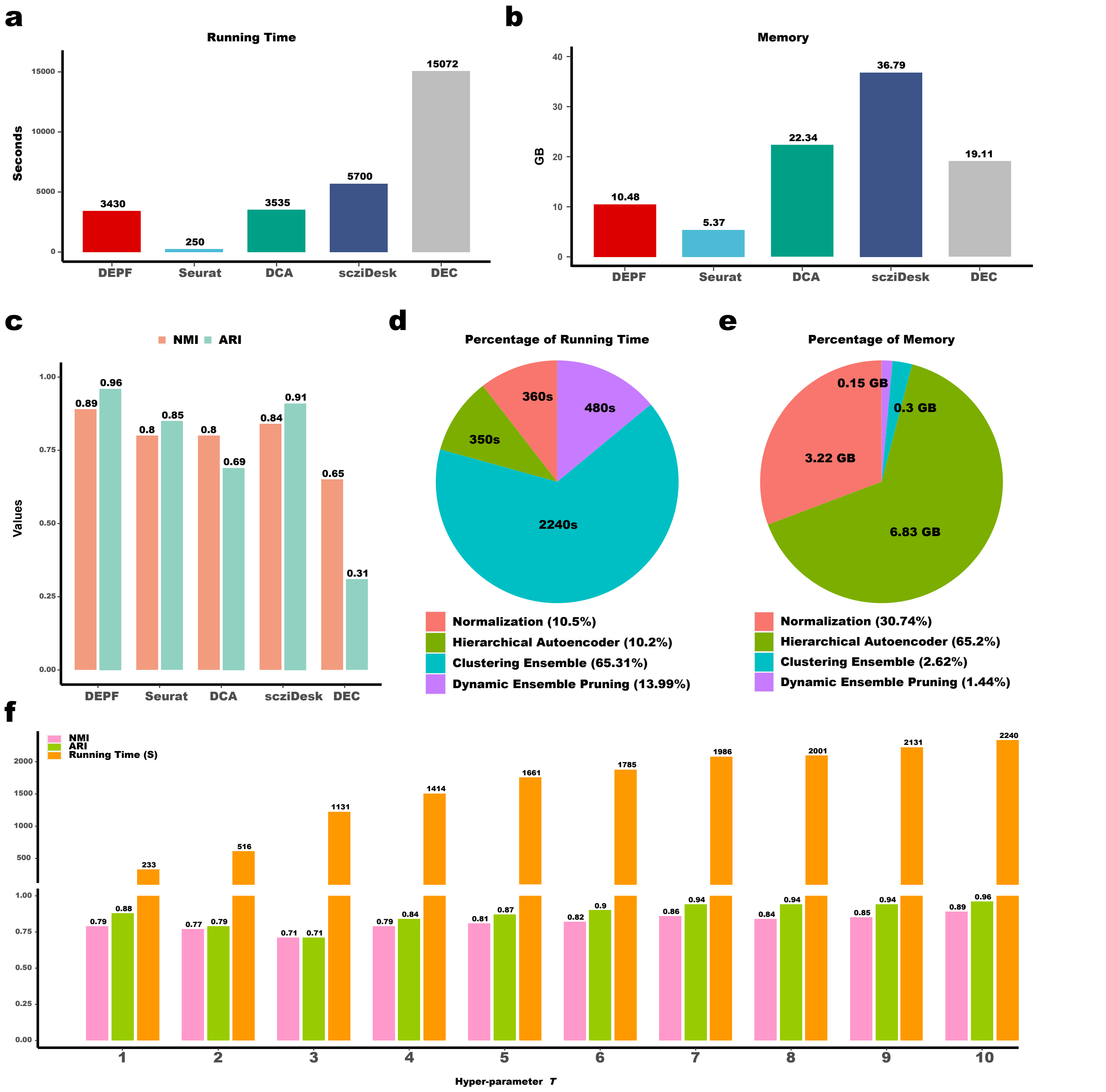

Figure S3: a) Running time of DEPF and 4 clustering algorithms. b) Memory of DEPF and 4 clustering algorithms. c) Comparisons of NMI and ARI values between DEPF and 4 clustering algorithms. d) Percentage of running time of different modules in DEPF. e) Percentage of memory of different modules in DEPF. f) The values of the NMI, ARI, and running time on different hyper-parameter T.

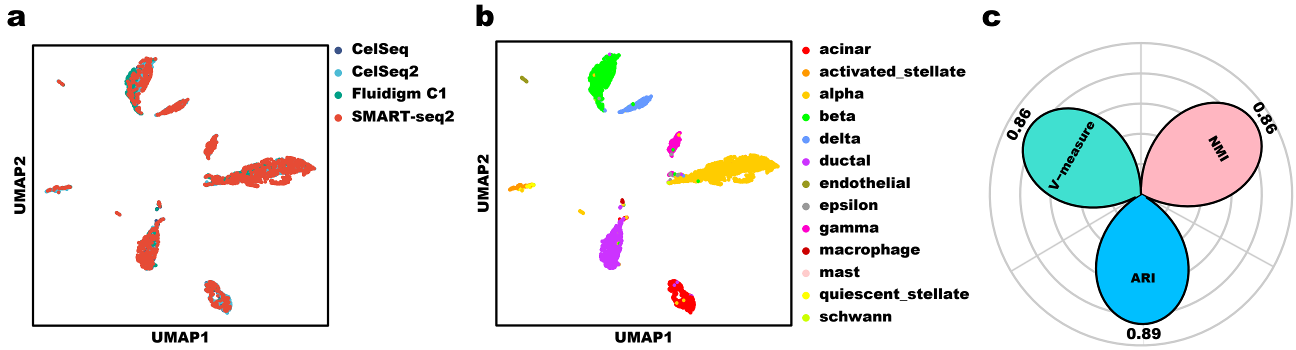

Figure S4: The performance of DEPF in batch effect correction and clustering on the pancreatic islet data generated by different scRNA-seq protocols. a) Visualizing DEPF for batch effect correction via UMAP. The cell batches are color-coded according to their respective batch data names: CelSeq, CelSeq2, Fluidigm C1, and SMART-Seq2. b) Visualizing DEPF for clustering via UMAP. The cell batches are color-coded based on their true labels. c) Clustering performance of DEPF evaluated with NMI, ARI and V-measure metrics.

5 DEPF can remove the batch effect in scRNA-seq data from different protocols

Many scRNA-seq datasets contain samples generated by different protocols, and there are batch differences between those samples that require data integration or batch effect correction. In this section, we investigated the performance of DEPF in correcting for batch effects by cascading four publicly human pancreas datasets originated from CelSeq [1], CelSeq2 [2], Fluidigm C1 [3], and SMART-Seq2 [4]. The combined dataset comprises 6321 cells, including 1004 cells from CelSeq, 2285 cells from CelSeq2, 638 cells from Fluidigm C1, and 2394 cells from SMART-Seq2. Initially, we employed UMAP to visualize the two-dimensional representation of the DEPF hidden space, as shown in Figure S4a. We observed that the cells from these four datasets are well integrated within the DEPF representation. Then, Figure S4b displays the scatter plots of cells from all batches of the human pancreas dataset in a 2D UMAP space, with cells colored according to their respective cell types. From this figure, we can observe that cells are separated according to cell types rather than batches and although a small number of cells with different color codes intermingle, the majority of cells sharing the same color code are distinctly clustered together, forming separate, well-defined groups. Additionally, we utilized metrics such as NMI, ARI, and V-mseasure to assess the robustness of DEPF. When these values are close to 1, it indicates that our algorithm can remove the batch effect well and can group the data effectively. Figure S4c provides the values of NMI, ARI, and V-measure metrics, all exceeding 0.85, demonstrating that our model has a good agreement between the clustering results and the true labels as well as that DEPF can do batch effect correction from different protocols.

6 DEPF can provide biological interpretation of scRNA-seq data

Table S9: biological processes of top-200 DEGs

Ontology	No.	ID	Description	GeneRatio	BgRatio	pvalue	p.adjust	qvalue	geneID	Count
BP	1	GO:0030198	extracellular matrix organization	47/179	393/18862	2.02227E-38	2.28183E-35	1.774E-35	DCN/COL1A2/COL3A1/A2M/CRISPLD2/MFAP4/BGN/ADAMTS1/COL1A1/SPARC/FN1/AEBP1/COL6A2/COL6A1/COL14A1/COL6A3/THBS1/CCDC80/TIMP1/FMOD/LAMA4/ITGA7/LAMB2/SMOC2/DDR2/SULF1/TIMP2/COL4A2/COL5A2/CTSK/SFRP2/LUM/PDGFRA/DPT/COL4A1/EFEMP2/NID1/COL12A1/ANTXR1/PHLDB1/FBLN5/LRP1/FBLN2/ADAMTS4/MMP2/PDGFA/MYH11	47
	2	GO:0043062	extracellular structure organization	47/179	394/18862	2.28014E-38	2.28183E-35	1.774E-35	DCN/COL1A2/COL3A1/A2M/CRISPLD2/MFAP4/BGN/ADAMTS1/COL1A1/SPARC/FN1/AEBP1/COL6A2/COL6A1/COL14A1/COL6A3/THBS1/CCDC80/TIMP1/FMOD/LAMA4/ITGA7/LAMB2/SMOC2/DDR2/SULF1/TIMP2/COL4A2/COL5A2/CTSK/SFRP2/LUM/PDGFRA/DPT/COL4A1/EFEMP2/NID1/COL12A1/ANTXR1/PHLDB1/FBLN5/LRP1/FBLN2/ADAMTS4/MMP2/PDGFA/MYH11	47
	3	GO:0045229	external encapsulating structure organization	47/179	396/18862	2.89572E-38	2.28183E-35	1.774E-35	DCN/COL1A2/COL3A1/A2M/CRISPLD2/MFAP4/BGN/ADAMTS1/COL1A1/SPARC/FN1/AEBP1/COL6A2/COL6A1/COL14A1/COL6A3/THBS1/CCDC80/TIMP1/FMOD/LAMA4/ITGA7/LAMB2/SMOC2/DDR2/SULF1/TIMP2/COL4A2/COL5A2/CTSK/SFRP2/LUM/PDGFRA/DPT/COL4A1/EFEMP2/NID1/COL12A1/ANTXR1/PHLDB1/FBLN5/LRP1/FBLN2/ADAMTS4/MMP2/PDGFA/MYH11	47
	4	GO:0030199	collagen fibril organization	13/179	54/18862	2.5752E-15	1.52194E-12	1.1832E-12	COL1A2/COL3A1/COL1A1/AEBP1/COL14A1/FMOD/DDR2/COL5A2/SFRP2/LUM/DPT/EFEMP2/COL12A1	13
	5	GO:0031589	cell-substrate adhesion	20/179	359/18862	2.2827E-10	1.07926E-07	8.3907E-08	COL3A1/COL1A1/FN1/THBS1/AXL/CCDC80/ITGA7/LGALS1/LAMB2/DLC1/EFEMP2/NID1/ANTXR1/FBLN5/NRP1/SORBS1/APOD/LRP1/FBLN2/SGCE	20
	6	GO:0006936	muscle contraction	19/179	352/18862	1.12101E-09	4.41677E-07	3.4338E-07	ACTA2/MYL9/TPM2/CALD1/PLN/HSPB6/LMOD1/VIM/SULF1/GSN/CNN1/EDNRA/CASQ2/SGCA/CRYAB/TPM1/MYLK/SORBS1/MYH11	19
	7	GO:0003012	muscle system process	21/179	453/18862	2.20874E-09	7.45923E-07	5.7992E-07	ACTA2/MYL9/TPM2/CALD1/IGFBP5/PLN/HSPB6/LMOD1/VIM/SULF1/GSN/CNN1/EDNRA/CASQ2/SGCA/CRYAB/TPM1/MYLK/SORBS1/SORBS2/MYH11	21
	8	GO:0085029	extracellular matrix assembly	8/179	45/18862	8.98416E-09	2.65482E-06	2.064E-06	COL1A2/MFAP4/LAMB2/EFEMP2/ANTXR1/PHLDB1/FBLN5/MYH11	8
	9	GO:0052547	regulation of peptidase activity	20/179	455/18862	1.33866E-08	3.51622E-06	2.7337E-06	A2M/PCOLCE/SERPING1/SERPINF1/FN1/C4B/COL6A3/C4A/THBS1/TIMP3/TIMP1/GSN/RCN3/TIMP2/TFPI/SFRP2/CRYAB/DLC1/ANTXR1/LRP1	20
	10	GO:0072001	renal system development	15/179	288/18862	1.09573E-07	2.5903E-05	2.0138E-05	ACTA2/DCN/PDGFRB/ADAMTS1/SERPINF1/ZBTB16/HEYL/LAMB2/SULF1/ID3/KANK2/PDGFRA/COL4A1/NID1/PDGFA	15
	11	GO:0010466	negative regulation of peptidase activity	14/179	257/18862	1.70822E-07	3.46001E-05	2.69E-05	A2M/SERPING1/SERPINF1/C4B/COL6A3/C4A/THBS1/TIMP3/TIMP1/TIMP2/TFPI/SFRP2/CRYAB/LRP1	14
	12	GO:0035987	endodermal cell differentiation	7/179	44/18862	1.75635E-07	3.46001E-05	2.69E-05	FN1/COL6A1/ITGA7/COL4A2/COL5A2/COL12A1/MMP2	7
	13	GO:0007160	cell-matrix adhesion	13/179	230/18862	3.13644E-07	5.70349E-05	4.4342E-05	COL3A1/FN1/THBS1/ITGA7/DLC1/EFEMP2/NID1/FBLN5/NRP1/SORBS1/APOD/LRP1/SGCE	13
	14	GO:0051346	negative regulation of hydrolase activity	18/179	456/18862	3.65996E-07	5.76956E-05	4.4856E-05	A2M/SERPING1/SERPINF1/C4B/COL6A3/C4A/THBS1/PLN/CNN3/TIMP3/TIMP1/TIMP2/TFPI/SFRP2/CRYAB/PPP1R14A/LRP1/ANGPTL4	18
	15	GO:0043687	post-translational protein modification	16/179	361/18862	3.66089E-07	5.76956E-05	4.4856E-05	SPARCL1/FN1/MXRA8/IGFBP7/C4A/IGFBP5/MFGE8/ZBTB16/TIMP1/IGFBP4/LGALS1/LAMB2/FSTL1/LTBP1/SDC2/EPAS1	16
	16	GO:0002576	platelet degranulation	10/179	128/18862	3.97636E-07	5.87507E-05	4.5676E-05	A2M/RARRES2/SERPING1/ISLR/SPARC/FN1/THBS1/TIMP3/TIMP1/PDGFA	10
	17	GO:0001655	urogenital system development	15/179	320/18862	4.24792E-07	5.9071E-05	4.5925E-05	ACTA2/DCN/PDGFRB/ADAMTS1/SERPINF1/ZBTB16/HEYL/LAMB2/SULF1/ID3/KANK2/PDGFRA/COL4A1/NID1/PDGFA	15
	18	GO:0001822	kidney development	14/179	280/18862	4.83796E-07	6.24321E-05	4.8538E-05	ACTA2/DCN/PDGFRB/ADAMTS1/SERPINF1/ZBTB16/HEYL/LAMB2/SULF1/ID3/KANK2/PDGFRA/NID1/PDGFA	14
	19	GO:0043277	apoptotic cell clearance	7/179	51/18862	5.01781E-07	6.24321E-05	4.8538E-05	C4B/C4A/THBS1/AXL/MFGE8/CCL2/LRP1	7
	20	GO:0061448	connective tissue development	13/179	243/18862	5.87017E-07	6.47627E-05	5.035E-05	ACTA2/BGN/PDGFRB/COL1A1/MGP/MUSTN1/ZBTB16/TIMP1/PRRX1/FRZB/SULF1/SFRP2/LUM	13
	21	GO:0030336	negative regulation of cell migration	15/179	330/18862	6.27163E-07	6.47627E-05	5.035E-05	DCN/COL3A1/SERPINF1/CXCL12/THBS1/IGFBP5/TIMP1/SULF1/PODN/SFRP2/CCL2/TPM1/DLC1/APOD/LRP1	15
	22	GO:0010951	negative regulation of endopeptidase activity	13/179	245/18862	6.44129E-07	6.47627E-05	5.035E-05	A2M/SERPING1/SERPINF1/C4B/COL6A3/C4A/THBS1/TIMP3/TIMP1/TIMP2/TFPI/SFRP2/CRYAB	13
	23	GO:0040013	negative regulation of locomotion	16/179	377/18862	6.51805E-07	6.47627E-05	5.035E-05	DCN/COL3A1/SERPINF1/CXCL12/THBS1/IGFBP5/TIMP1/SULF1/PODN/SFRP2/CCL2/TPM1/DLC1/NRP1/APOD/LRP1	16
	24	GO:0001706	endoderm formation	7/179	53/18862	6.57489E-07	6.47627E-05	5.035E-05	FN1/COL6A1/ITGA7/COL4A2/COL5A2/COL12A1/MMP2	7
	25	GO:0001501	skeletal system development	18/179	486/18862	9.20055E-07	8.70004E-05	6.7639E-05	COL1A2/COL3A1/BGN/COL1A1/MGP/MUSTN1/ZBTB16/TIMP1/PRRX1/FRZB/SULF1/COL5A2/SFRP2/LUM/PDGFRA/PRELP/ADAMTS4/MMP2	18
	26	GO:0022617	extracellular matrix disassembly	8/179	81/18862	1.00264E-06	9.11629E-05	7.0875E-05	A2M/TIMP1/DDR2/TIMP2/CTSK/LRP1/ADAMTS4/MMP2	8
	27	GO:0048008	platelet-derived growth factor receptor signaling pathway	7/179	57/18862	1.09229E-06	9.24849E-05	7.1903E-05	PDGFRB/PDGFRL/PDGFRA/NRP1/APOD/LRP1/PDGFA	7
	28	GO:2000146	negative regulation of cell motility	15/179	345/18862	1.09542E-06	9.24849E-05	7.1903E-05	DCN/COL3A1/SERPINF1/CXCL12/THBS1/IGFBP5/TIMP1/SULF1/PODN/SFRP2/CCL2/TPM1/DLC1/APOD/LRP1	15
	29	GO:0010810	regulation of cell-substrate adhesion	12/179	218/18862	1.19669E-06	9.75508E-05	7.5841E-05	COL1A1/FN1/THBS1/CCDC80/LGALS1/DLC1/EFEMP2/NID1/NRP1/APOD/LRP1/FBLN2	12
	30	GO:0051271	negative regulation of cellular component movement	15/179	352/18862	1.40651E-06	0.000110833	8.6167E-05	DCN/COL3A1/SERPINF1/CXCL12/THBS1/IGFBP5/TIMP1/SULF1/PODN/SFRP2/CCL2/TPM1/DLC1/APOD/LRP1	15
	31	GO:0048251	elastic fiber assembly	4/179	10/18862	1.57529E-06	0.000117623	9.1446E-05	MFAP4/EFEMP2/FBLN5/MYH11	4
	32	GO:0051216	cartilage development	11/179	185/18862	1.59219E-06	0.000117623	9.1446E-05	BGN/COL1A1/MGP/MUSTN1/ZBTB16/TIMP1/PRRX1/FRZB/SULF1/SFRP2/LUM	11
	33	GO:0071230	cellular response to amino acid stimulus	7/179	61/18862	1.7458E-06	0.000125063	9.723E-05	COL1A2/COL1A1/COL6A1/COL5A2/PDGFRA/COL4A1/MMP2	7
	34	GO:0032835	glomerulus development	7/179	62/18862	1.95236E-06	0.000135747	0.00010554	ACTA2/PDGFRB/HEYL/LAMB2/SULF1/PDGFRA/NID1	7
	35	GO:0007517	muscle organ development	14/179	317/18862	2.10955E-06	0.000142485	0.00011078	TAGLN/DCN/COL6A3/ITGA7/HEYL/ID3/FHL1/SGCA/NUPR1/CRYAB/TPM1/EFEMP2/MYLK/SGCE	14
	36	GO:0031032	actomyosin structure organization	11/179	191/18862	2.17456E-06	0.000142796	0.00011102	PDGFRB/CNN3/LMOD1/CNN1/TPM1/PDGFRA/DLC1/NRP1/SORBS1/MYH11/CSRP2	11
	37	GO:0031099	regeneration	11/179	192/18862	2.28793E-06	0.00014618	0.00011365	NNMT/CXCL12/MUSTN1/AXL/PRRX1/LAMB2/MAP1B/GSN/SGCA/APOD/LRP1	11

Table S10: biological processes of top-200 DEGs (continued 1)

Ontology	No.	ID	Description	GeneRatio	BgRatio	pvalue	p.adjust	qvalue	geneID	Count
BP	38	GO:1905048	regulation of metallopeptidase activity	5/179	25/18862	3.31508E-06	0.000206233	0.00016034	TIMP3/TIMP1/TIMP2/ANTXR1/LRP1	5
	39	GO:0038063	collagen-activated tyrosine kinase receptor signaling pathway	4/179	12/18862	3.65836E-06	0.000221753	0.0001724	COL1A1/DDR2/COL4A2/COL4A1	4
	40	GO:0071229	cellular response to acid chemical	7/179	69/18862	4.05056E-06	0.000239388	0.00018611	COL1A2/COL1A1/COL6A1/COL5A2/PDGFRA/COL4A1/MMP2	7
	41	GO:0010927	cellular component assembly involved in morphogenesis	8/179	100/18862	4.97768E-06	0.000285926	0.00022229	PDGFRB/PMP22/LMOD1/TPM1/PDGFRA/PHLDB1/MYH11/CSRP2	8
	42	GO:0007596	blood coagulation	14/179	342/18862	5.07991E-06	0.000285926	0.00022229	COL1A2/COL3A1/A2M/MYL9/SERPING1/COL1A1/FN1/THBS1/AXL/C1QTNF1/TFPI/PDGFRA/PDGFA/EHD2	14
	43	GO:1903053	regulation of extracellular matrix organization	6/179	47/18862	5.22197E-06	0.000287087	0.0002232	AEBP1/DDR2/EFEMP2/ANTXR1/PHLDB1/LRP1	6
	44	GO:0035924	cellular response to vascular endothelial growth factor stimulus	7/179	72/18862	5.39991E-06	0.000289346	0.00022495	DCN/PDGFRB/SMOC2/NR4A1/PDGFRA/NRP1/PGF	7
	45	GO:0007599	hemostasis	14/179	346/18862	5.80297E-06	0.000289346	0.00022495	COL1A2/COL3A1/A2M/MYL9/SERPING1/COL1A1/FN1/THBS1/AXL/C1QTNF1/TFPI/PDGFRA/PDGFA/EHD2	14
	46	GO:0045861	negative regulation of proteolysis	14/179	346/18862	5.80297E-06	0.000289346	0.00022495	A2M/SERPING1/SERPINF1/C4B/COL6A3/C4A/THBS1/TIMP3/TIMP1/TIMP2/TFPI/SFRP2/CRYAB/LRP1	14
	47	GO:0030195	negative regulation of blood coagulation	6/179	48/18862	5.92128E-06	0.000289346	0.00022495	SERPING1/THBS1/C1QTNF1/TFPI/PDGFRA/PDGFA	6
	48	GO:0038084	vascular endothelial growth factor signaling pathway	6/179	48/18862	5.92128E-06	0.000289346	0.00022495	DCN/PDGFRB/SMOC2/PDGFRA/NRP1/PGF	6
	49	GO:0050817	coagulation	14/179	347/18862	5.99744E-06	0.000289346	0.00022495	COL1A2/COL3A1/A2M/MYL9/SERPING1/COL1A1/FN1/THBS1/AXL/C1QTNF1/TFPI/PDGFRA/PDGFA/EHD2	14
	50	GO:1900047	negative regulation of hemostasis	6/179	49/18862	6.69471E-06	0.000316526	0.00024608	SERPING1/THBS1/C1QTNF1/TFPI/PDGFRA/PDGFA	6
	51	GO:0035791	platelet-derived growth factor receptor-beta signaling pathway	4/179	14/18862	7.28883E-06	0.000331361	0.00025762	PDGFRB/PDGFRL/LRP1/PDGFA	4
	52	GO:1905049	negative regulation of metallopeptidase activity	4/179	14/18862	7.28883E-06	0.000331361	0.00025762	TIMP3/TIMP1/TIMP2/LRP1	4
	53	GO:0007492	endoderm development	7/179	76/18862	7.76245E-06	0.000346235	0.00026918	FN1/COL6A1/ITGA7/COL4A2/COL5A2/COL12A1/MMP2	7
	54	GO:0032963	collagen metabolic process	8/179	109/18862	9.4523E-06	0.000409174	0.00031811	COL1A2/MFAP4/PDGFRB/COL1A1/VIM/RCN3/CTSK/MMP2	8
	55	GO:0050819	negative regulation of coagulation	6/179	52/18862	9.51969E-06	0.000409174	0.00031811	SERPING1/THBS1/C1QTNF1/TFPI/PDGFRA/PDGFA	6
	56	GO:0038065	collagen-activated signaling pathway	4/179	15/18862	9.86572E-06	0.000416474	0.00032379	COL1A1/DDR2/COL4A2/COL4A1	4
	57	GO:0016049	cell growth	16/179	470/18862	1.10184E-05	0.000456976	0.00035528	FN1/CXCL12/IGFBP7/IGFBP5/IGFBP4/LAMB2/FRZB/MAP1B/SFRP2/FHL1/CRYAB/DPYSL2/NRP1/SORBS2/LRP1/MEG3	16
	58	GO:0014910	regulation of smooth muscle cell migration	7/179	81/18862	1.18645E-05	0.000483581	0.00037596	PDGFRB/ADAMTS1/IGFBP5/TPM1/NRP1/LRP1/PDGFA	7
	59	GO:0001704	formation of primary germ layer	8/179	115/18862	1.40232E-05	0.000556473	0.00043263	FN1/COL6A1/ITGA7/COL4A2/COL5A2/SFRP2/COL12A1/MMP2	8
	60	GO:0052548	regulation of endopeptidase activity	15/179	426/18862	1.41237E-05	0.000556473	0.00043263	A2M/SERPING1/SERPINF1/C4B/COL6A3/C4A/THBS1/TIMP3/TIMP1/GSN/TIMP2/TFPI/SFRP2/CRYAB/DLC1	15
	61	GO:0044273	sulfur compound catabolic process	6/179	56/18862	1.47131E-05	0.000570194	0.0004433	DCN/BGN/GGT5/FMOD/LUM/PRELP	6
	62	GO:0018149	peptide cross-linking	5/179	35/18862	1.87573E-05	0.000715199	0.00055603	DCN/COL3A1/BGN/FN1/THBS1	5
	63	GO:0030168	platelet activation	9/179	157/18862	1.9372E-05	0.000724805	0.0005635	COL1A2/COL3A1/MYL9/COL1A1/FN1/AXL/C1QTNF1/PDGFRA/PDGFA	9
	64	GO:0045765	regulation of angiogenesis	13/179	335/18862	1.96225E-05	0.000724805	0.0005635	DCN/ADAMTS1/SERPINF1/SPARC/THBS1/HSPB6/SMOC2/SULF1/COL4A2/SFRP2/THBS2/PGF/ANGPTL4	13
	65	GO:0030239	myofibril assembly	6/179	59/18862	1.9943E-05	0.000725311	0.00056389	PDGFRB/LMOD1/TPM1/PDGFRA/MYH11/CSRP2	6
	66	GO:0014909	smooth muscle cell migration	7/179	88/18862	2.04872E-05	0.000733814	0.0005705	PDGFRB/ADAMTS1/IGFBP5/TPM1/NRP1/LRP1/PDGFA	7
	67	GO:0051895	negative regulation of focal adhesion assembly	4/179	18/18862	2.16292E-05	0.000741036	0.00057612	THBS1/DLC1/APOD/LRP1	4
	68	GO:0061298	retina vasculature development in camera-type eye	4/179	18/18862	2.16292E-05	0.000741036	0.00057612	PDGFRB/PDGFRA/COL4A1/NRP1	4
	69	GO:0150118	negative regulation of cell-substrate junction organization	4/179	18/18862	2.16292E-05	0.000741036	0.00057612	THBS1/DLC1/APOD/LRP1	4
	70	GO:0048660	regulation of smooth muscle cell proliferation	9/179	160/18862	2.25189E-05	0.000760497	0.00059125	PDGFRB/ADAMTS1/THBS1/IGFBP5/CNN1/TPM1/EFEMP2/APOD/MMP2	9
	71	GO:1901342	regulation of vasculature development	13/179	341/18862	2.36275E-05	0.000786697	0.00061162	DCN/ADAMTS1/SERPINF1/SPARC/THBS1/HSPB6/SMOC2/SULF1/COL4A2/SFRP2/THBS2/PGF/ANGPTL4	13
	72	GO:0048659	smooth muscle cell proliferation	9/179	162/18862	2.48498E-05	0.000815903	0.00063432	PDGFRB/ADAMTS1/THBS1/IGFBP5/CNN1/TPM1/EFEMP2/APOD/MMP2	9
	73	GO:0030324	lung development	9/179	163/18862	2.60901E-05	0.00084489	0.00065686	CRISPLD2/PDGFRB/SPARC/IGFBP5/FGF7/RCN3/PDGFRA/EPAS1/PDGFA	9
	74	GO:0006027	glycosaminoglycan catabolic process	6/179	62/18862	2.65768E-05	0.00084902	0.00066007	DCN/BGN/FMOD/LUM/SDC2/PRELP	6
	75	GO:1903034	regulation of response to wounding	9/179	164/18862	2.73825E-05	0.000863096	0.00067102	SERPING1/THBS1/SMOC2/C1QTNF1/TFPI/PDGFRA/MYLK/LRP1/PDGFA	9
	76	GO:0030323	respiratory tube development	9/179	167/18862	3.15906E-05	0.000982633	0.00076395	CRISPLD2/PDGFRB/SPARC/IGFBP5/FGF7/RCN3/PDGFRA/EPAS1/PDGFA	9
	77	GO:0045773	positive regulation of axon extension	5/179	39/18862	3.22617E-05	0.000990475	0.00077005	FN1/CXCL12/MAP1B/NRP1/LRP1	5
	78	GO:0044342	type B pancreatic cell proliferation	4/179	20/18862	3.37416E-05	0.001022631	0.00079505	IGFBP5/IGFBP4/NR4A1/NUPR1	4
	79	GO:0001558	regulation of cell growth	14/179	406/18862	3.46031E-05	0.001032364	0.00080261	FN1/CXCL12/IGFBP7/IGFBP5/IGFBP4/FRZB/MAP1B/SFRP2/FHL1/CRYAB/DPYSL2/NRP1/LRP1/MEG3	14
	80	GO:0007568	aging	12/179	304/18862	3.49362E-05	0.001032364	0.00080261	DCN/PDGFRB/SERPINF1/IGFBP5/TIMP1/GSN/TIMP2/COL4A2/CRYAB/PRELP/APOD/LRP1	12
	81	GO:0061041	regulation of wound healing	8/179	131/18862	3.60631E-05	0.001052508	0.00081827	SERPING1/THBS1/SMOC2/C1QTNF1/TFPI/PDGFRA/MYLK/PDGFA	8
	82	GO:0030193	regulation of blood coagulation	6/179	66/18862	3.80679E-05	0.00109747	0.00085323	SERPING1/THBS1/C1QTNF1/TFPI/PDGFRA/PDGFA	6
	83	GO:0010812	negative regulation of cell-substrate adhesion	6/179	67/18862	4.1486E-05	0.001167534	0.0009077	COL1A1/THBS1/LGALS1/DLC1/APOD/LRP1	6
	84	GO:1900046	regulation of hemostasis	6/179	67/18862	4.1486E-05	0.001167534	0.0009077	SERPING1/THBS1/C1QTNF1/TFPI/PDGFRA/PDGFA	6
	85	GO:0006026	aminoglycan catabolic process	6/179	68/18862	4.51457E-05	0.001255582	0.00097615	DCN/BGN/FMOD/LUM/SDC2/PRELP	6
	86	GO:0007044	cell-substrate junction assembly	7/179	100/18862	4.69578E-05	0.001290794	0.00100353	FN1/THBS1/DLC1/NRP1/SORBS1/APOD/LRP1	7
	87	GO:0014812	muscle cell migration	7/179	101/18862	5.00554E-05	0.001360125	0.00105743	PDGFRB/ADAMTS1/IGFBP5/TPM1/NRP1/LRP1/PDGFA	7
	88	GO:0048844	artery morphogenesis	6/179	70/18862	5.32403E-05	0.00141623	0.00110105	PDGFRB/PRRX1/EFEMP2/MYLK/NRP1/LRP1	6
	89	GO:0043200	response to amino acid	7/179	102/18862	5.33183E-05	0.00141623	0.00110105	COL1A2/COL1A1/COL6A1/COL5A2/PDGFRA/COL4A1/MMP2	7
	90	GO:0007369	gastrulation	9/179	179/18862	5.43404E-05	0.001427342	0.00110969	FN1/COL6A1/ITGA7/COL4A2/COL5A2/SFRP2/COL12A1/PHLDB1/MMP2	9

Table S11: biological processes of top-200 DEGs (continued 2)

Ontology	No.	ID	Description	GeneRatio	BgRatio	pvalue	p.adjust	qvalue	geneID	Count
BP	91	GO:0060537	muscle tissue development	13/179	371/18862	5.63383E-05	0.001463559	0.00113785	DCN/PDGFRB/IGFBP5/PLN/HEYL/NUPR1/TPM1/PDGFRA/ EFEMP2/MYLK/SORBS2/MYH11/CSRP2	13
	92	GO:0000302	response to reactive oxygen species	10/179	224/18862	5.73334E-05	0.001466727	0.00114031	PDGFRB/COL1A1/SOD3/AXL/CRYAB/TPM1/PDGFRA/ FBLN5/APOD/MMP2	10
	93	GO:0050818	regulation of coagulation	6/179	71/18862	5.77012E-05	0.001466727	0.00114031	SERPING1/THBS1/C1QTNF1/TFPI/PDGFRA/PDGFA	6
	94	GO:0050673	epithelial cell proliferation	14/179	428/18862	6.11763E-05	0.001538519	0.00119612	SERPINF1/SPARC/CXCL12/THBS1/IGFBP5/IGFBP4/FGF7/ SULF1/NR4A1/SFRP2/CCL2/NUPR1/NRP1/PGF	14
	95	GO:0014911	positive regulation of smooth muscle cell migration	5/179	45/18862	6.53813E-05	0.001626962	0.00126488	PDGFRB/ADAMTS1/IGFBP5/NRP1/LRP1	5
	96	GO:0150115	cell-substrate junction organization	7/179	106/18862	6.81614E-05	0.001678476	0.00130493	FN1/THBS1/DLC1/NRP1/SORBS1/APOD/LRP1	7
	97	GO:0006024	glycosaminoglycan biosynthetic process	7/179	107/18862	7.23559E-05	0.001723425	0.00133988	DCN/BGN/PDGFRB/FMOD/LUM/SDC2/PRELP	7
	98	GO:0010171	body morphogenesis	5/179	46/18862	7.27947E-05	0.001723425	0.00133988	CRISPLD2/COL1A1/PDGFRA/PHLDB1/MMP2	5
	99	GO:0048146	positive regulation of fibroblast proliferation	5/179	46/18862	7.27947E-05	0.001723425	0.00133988	PDGFRB/FN1/DDR2/PDGFRA/PDGFA	5
	100	GO:0061045	negative regulation of wound healing	6/179	74/18862	7.29029E-05	0.001723425	0.00133988	SERPING1/THBS1/C1QTNF1/TFPI/PDGFRA/PDGFA	6
	101	GO:0016525	negative regulation of angiogenesis	8/179	146/18862	7.78618E-05	0.00182243	0.00141685	DCN/ADAMTS1/SERPINF1/SPARC/THBS1/SULF1/ COL4A2/THBS2	8
	102	GO:0060541	respiratory system development	9/179	188/18862	7.93572E-05	0.001839219	0.0014299	CRISPLD2/PDGFRB/SPARC/IGFBP5/FGF7/RCN3/ PDGFRA/EPAS1/PDGFA	9
	103	GO:2000181	negative regulation of blood vessel morphogenesis	8/179	148/18862	8.56631E-05	0.001966093	0.00152854	DCN/ADAMTS1/SERPINF1/SPARC/THBS1/SULF1/ COL4A2/THBS2	8
	104	GO:1901343	negative regulation of vasculature development	8/179	149/18862	8.98003E-05	0.00204123	0.00158696	DCN/ADAMTS1/SERPINF1/SPARC/THBS1/SULF1/ COL4A2/THBS2	8
	105	GO:0032970	regulation of actin filament-based process	13/179	389/18862	9.08855E-05	0.002046221	0.00159084	PDGFRB/CXCL12/PLN/LMOD1/GSN/TPM1/PDGFRA/ DLC1/NRP1/EPS8/SYNPO2/LRP1/PDGFA	13
	106	GO:0060485	mesenchyme development	11/179	287/18862	9.66675E-05	0.002155868	0.00167608	ACTA2/PDGFRB/COL1A1/FN1/HEYL/FRZB/SFRP2/EDNRA/ TGFB1I1/PHLDB1/NRP1	11
	107	GO:0048145	regulation of fibroblast proliferation	6/179	79/18862	0.000105192	0.002324053	0.00180684	PDGFRB/FN1/DDR2/NUPR1/PDGFRA/PDGFA	6
	108	GO:0006023	aminoglycan biosynthetic process	7/179	114/18862	0.000108003	0.002342387	0.00182109	DCN/BGN/PDGFRB/FMOD/LUM/SDC2/PRELP	7
	109	GO:0048675	axon extension	7/179	114/18862	0.000108003	0.002342387	0.00182109	FN1/CXCL12/LAMB2/MAP1B/DPYSL2/NRP1/LRP1	7
	110	GO:0048144	fibroblast proliferation	6/179	80/18862	0.000112835	0.002424929	0.00188526	PDGFRB/FN1/DDR2/NUPR1/PDGFRA/PDGFA	6
	111	GO:0030449	regulation of complement activation	7/179	115/18862	0.000114095	0.002429911	0.00188914	A2M/SERPING1/C1R/C4B/C4A/C1S/CFH	7
	112	GO:0001503	ossification	13/179	401/18862	0.000123015	0.002596488	0.00201864	COL1A2/COL1A1/MGP/SPARC/COL6A1/IGFBP5/ZBTB16/ ASPN/DDR2/ID3/COL5A2/SFRP2/MMP2	13
	113	GO:0032836	glomerular basement membrane development	3/179	11/18862	0.00013112	0.002743079	0.00213261	LAMB2/SULF1/NID1	3
	114	GO:0072376	protein activation cascade	4/179	28/18862	0.000134383	0.002762452	0.00214767	A2M/SERPING1/FN1/TFPI	4
	115	GO:0072378	blood coagulation, fibrin clot formation	4/179	28/18862	0.000134383	0.002762452	0.00214767	A2M/SERPING1/FN1/TFPI	4
	116	GO:1904705	regulation of vascular associated smooth muscle cell proliferation	6/179	83/18862	0.000138443	0.002797248	0.00217472	ADAMTS1/IGFBP5/CNN1/TPM1/EFEMP2/MMP2	6
	117	GO:1990874	vascular associated smooth muscle cell proliferation	6/179	83/18862	0.000138443	0.002797248	0.00217472	ADAMTS1/IGFBP5/CNN1/TPM1/EFEMP2/MMP2	6
	118	GO:0032956	regulation of actin cytoskeleton organization	12/179	352/18862	0.000141213	0.002807363	0.00218259	PDGFRB/CXCL12/LMOD1/GSN/TPM1/PDGFRA/DLC1/ NRP1/EPS8/SYNPO2/LRP1/PDGFA	12
	119	GO:0001101	response to acid chemical	7/179	119/18862	0.000141318	0.002807363	0.00218259	COL1A2/COL1A1/COL6A1/COL5A2/PDGFRA/COL4A1/ MMP2	7
	120	GO:0035023	regulation of Rho protein signal transduction	6/179	84/18862	0.000147931	0.002914244	0.00226568	COL3A1/PDGFRB/KANK2/DLC1/NRP1/EPS8	6
	121	GO:0060325	face morphogenesis	4/179	29/18862	0.000154735	0.003023085	0.0023503	CRISPLD2/COL1A1/PDGFRA/MMP2	4
	122	GO:0048041	focal adhesion assembly	6/179	85/18862	0.000157927	0.003060168	0.00237913	THBS1/DLC1/NRP1/SORBS1/APOD/LRP1	6
	123	GO:0001867	complement activation, lectin pathway	3/179	12/18862	0.000173607	0.003308459	0.00257217	A2M/MFAP4/SERPING1	3
	124	GO:0042340	keratan sulfate catabolic process	3/179	12/18862	0.000173607	0.003308459	0.00257217	FMOD/LUM/PRELP	3
	125	GO:0001667	ameboidal-type cell migration	14/179	473/18862	0.00017494	0.003308459	0.00257217	DCN/SERPINF1/SPARC/FN1/THBS1/TIMP1/SMOC2/ FGF7/DDR2/NR4A1/FSTL1/KANK2/NRP1/PTP4A3	14
	126	GO:0071711	basement membrane organization	4/179	30/18862	0.000177224	0.003325058	0.00258507	LAMB2/COL4A1/NID1/PHLDB1	4
	127	GO:0090130	tissue migration	12/179	365/18862	0.000197548	0.003677189	0.00285884	ACTA2/DCN/SERPINF1/SPARC/THBS1/SMOC2/ FGF7/NR4A1/FSTL1/KANK2/NRP1/PTP4A3	12
	128	GO:0032387	negative regulation of intracellular transport	5/179	57/18862	0.000204406	0.003773345	0.00293359	PLN/MAP1B/CRYAB/PKIG/APOD	5
	129	GO:0055001	muscle cell development	8/179	168/18862	0.000205906	0.003773345	0.00293359	PDGFRB/LMOD1/TPM1/PDGFRA/EFEMP2/SORBS2/ MYH11/CSRP2	8
	130	GO:1903035	negative regulation of response to wounding	6/179	90/18862	0.000216213	0.003931751	0.00305674	SERPING1/THBS1/C1QTNF1/TFPI/PDGFRA/PDGFA	6
	131	GO:0031102	neuron projection regeneration	5/179	58/18862	0.000221986	0.004005915	0.0031144	PRRX1/LAMB2/MAP1B/APOD/LRP1	5
	132	GO:1902903	regulation of supramolecular fiber organization	12/179	370/18862	0.000223826	0.004008512	0.00311642	AEBP1/CXCL12/LMOD1/MAP1B/GSN/CRYAB/ TPM1/DLC1/EFEMP2/NRP1/EPS8/SYNPO2	12
	133	GO:0035767	endothelial cell chemotaxis	4/179	32/18862	0.000229133	0.004042312	0.0031427	THBS1/SMOC2/NR4A1/NRP1	4
	134	GO:1901889	negative regulation of cell junction assembly	4/179	32/18862	0.000229133	0.004042312	0.0031427	THBS1/DLC1/APOD/LRP1	4
	135	GO:0030516	regulation of axon extension	6/179	92/18862	0.000243788	0.004268998	0.00331894	FN1/CXCL12/MAP1B/DPYSL2/NRP1/LRP1	6
	136	GO:0050678	regulation of epithelial cell proliferation	12/179	374/18862	0.000246941	0.004292422	0.00333715	SERPINF1/SPARC/CXCL12/THBS1/FGF7/SULF1/ NR4A1/SFRP2/CCL2/NUPR1/NRP1/PGF	12
	137	GO:0035909	aorta morphogenesis	4/179	33/18862	0.000258816	0.004433639	0.00344694	PDGFRB/EFEMP2/MYLK/LRP1	4
	138	GO:0060323	head morphogenesis	4/179	33/18862	0.000258816	0.004433639	0.00344694	CRISPLD2/COL1A1/PDGFRA/MMP2	4
	139	GO:0045444	fat cell differentiation	9/179	221/18862	0.000268391	0.004555893	0.00354198	RARRES2/ZBTB16/FRZB/NR4A1/SFRP2/TGFB1I1/ PDGFRA/ADIRF/DIO2	9
	140	GO:0048771	tissue remodeling	8/179	175/18862	0.000271735	0.004555893	0.00354198	BGN/IGFBP5/AXL/TIMP1/PDK4/DDR2/EPAS1/MMP2	8
	141	GO:0051897	positive regulation of protein kinase B signaling	8/179	175/18862	0.000271735	0.004555893	0.00354198	PDGFRB/THBS1/IGFBP5/AXL/FGF7/C1QTNF1/ PDGFRA/PDGFA	8
	142	GO:1904035	regulation of epithelial cell apoptotic process	6/179	94/18862	0.000274057	0.004562477	0.0035471	THBS1/CD248/GSN/CCL2/NUPR1/ANGPTL4	6
	143	GO:0033002	muscle cell proliferation	9/179	222/18862	0.000277485	0.004587233	0.00356635	PDGFRB/ADAMTS1/THBS1/IGFBP5/CNN1/TPM1/ EFEMP2/APOD/MMP2	9
	144	GO:0043491	protein kinase B signaling	10/179	273/18862	0.000288959	0.004696252	0.00365111	PDGFRB/THBS1/IGFBP5/AXL/FGF7/RCN3/ C1QTNF1/CCL2/PDGFRA/PDGFA	10
	145	GO:0060840	artery development	6/179	95/18862	0.000290259	0.004696252	0.00365111	PDGFRB/PRRX1/EFEMP2/MYLK/NRP1/LRP1	6
	146	GO:0001569	branching involved in blood vessel morphogenesis	4/179	34/18862	0.000291165	0.004696252	0.00365111	SFRP2/EDNRA/COL4A1/NRP1	4
	147	GO:0001936	regulation of endothelial cell proliferation	8/179	177/18862	0.000293416	0.004696252	0.00365111	SPARC/CXCL12/THBS1/SULF1/NR4A1/CCL2/NRP1/PGF	8
	148	GO:0007266	Rho protein signal transduction	7/179	134/18862	0.000294012	0.004696252	0.00365111	COL1A2/COL3A1/PDGFRB/KANK2/DLC1/NRP1/EPS8	7
	149	GO:0050920	regulation of chemotaxis	9/179	224/18862	0.000296439	0.00470324	0.00365654	RARRES2/PDGFRB/CXCL12/THBS1/SMOC2/ CCL2/PDGFRA/NRP1/PGF	9
	150	GO:0006956	complement activation	8/179	178/18862	0.000304776	0.004803269	0.00373431	A2M/MFAP4/SERPING1/C1R/C4B/C4A/C1S/CFH	8

Table S12: biological processes of top-200 DEGs (continued 3)

Ontology	No.	ID	Description	GeneRatio	BgRatio	pvalue	p.adjust	qvalue	geneID	Count
BP	151	GO:0048662	negative regulation of smooth muscle cell proliferation	5/179	63/18862	0.000327759	0.005131272	0.003989314	IGFBP5/CNN1/TPM1/EFEMP2/APOD	5
	152	GO:0006979	response to oxidative stress	13/179	444/18862	0.000331407	0.00515425	0.004007178	PDGFRB/COL1A1/GPX3/SOD3/AXL/CRYAB/TPM1 /PDGFRA/FBLN5/EPAS1/APOD/MMP2/MSRB3	13
	153	GO:0072224	metanephric glomerulus development	3/179	15/18862	0.000351597	0.005312691	0.004130358	PDGFRB/LAMB2/PDGFRA	3
	154	GO:0002920	regulation of humoral immune response	7/179	138/18862	0.000351619	0.005312691	0.004130358	A2M/SERPING1/C1R/C4B/C4A/C1S/CFH	7
	155	GO:0072006	nephron development	7/179	138/18862	0.000351619	0.005312691	0.004130358	ACTA2/PDGFRB/HEYL/LAMB2/SULF1/PDGFRA/NID1	7
	156	GO:0051893	regulation of focal adhesion assembly	5/179	64/18862	0.000352831	0.005312691	0.004130358	THBS1/DLC1/NRP1/APOD/LRP1	5
	157	GO:0090109	regulation of cell-substrate junction assembly	5/179	64/18862	0.000352831	0.005312691	0.004130358	THBS1/DLC1/NRP1/APOD/LRP1	5
	158	GO:0050921	positive regulation of chemotaxis	7/179	139/18862	0.000367346	0.005496245	0.004273062	RARRES2/PDGFRB/CXCL12/THBS1/SMOC2/NRP1/PGF	7
	159	GO:0048762	mesenchymal cell differentiation	9/179	231/18862	0.000371427	0.005522353	0.00429336	COL1A1/FN1/HEYL/FRZB/SFRP2/EDNRA/TGFB1I1 /PHLDB1/NRP1	9
	160	GO:0006939	smooth muscle contraction	6/179	100/18862	0.000382887	0.005657152	0.00439816	ACTA2/SULF1/CNN1/EDNRA/MYLK/MYH11	6
	161	GO:0045600	positive regulation of fat cell differentiation	5/179	66/18862	0.000407299	0.005980465	0.00464952	RARRES2/ZBTB16/FRZB/SFRP2/ADIRF	5
	162	GO:0044344	cellular response to fibroblast growth factor stimulus	7/179	142/18862	0.000417938	0.006098805	0.004741524	COL1A1/THBS1/SMOC2/FGF7/SULF1/NR4A1/CCL2	7
	163	GO:0050766	positive regulation of phagocytosis	5/179	67/18862	0.0004368	0.006296313	0.004895076	C4B/C4A/MFGE8/CCL2/LRP1	5
	164	GO:0050918	positive chemotaxis	5/179	67/18862	0.0004368	0.006296313	0.004895076	CXCL12/FGF7/NRP1/LRP1/PGF	5
	165	GO:0055002	striated muscle cell development	6/179	103/18862	0.000448648	0.006427908	0.004997385	PDGFRB/LMOD1/TPM1/PDGFRA/MYH11/CSRP2	6
	166	GO:0061387	regulation of extent of cell growth	6/179	104/18862	0.000472421	0.006727726	0.005230479	FN1/CXCL12/MAP1B/DPYSL2/NRP1/LRP1	6
	167	GO:0001935	endothelial cell proliferation	8/179	191/18862	0.000488114	0.006909595	0.005371873	SPARC/CXCL12/THBS1/SULF1/NR4A1/CCL2/NRP1/PGF	8
	168	GO:0150116	regulation of cell-substrate junction organization	5/179	69/18862	0.000500604	0.007044218	0.005476536	THBS1/DLC1/NRP1/APOD/LRP1	5
	169	GO:0010544	negative regulation of platelet activation	3/179	17/18862	0.000518173	0.00724829	0.005635192	C1QTNF1/PDGFRA/PDGFA	3
	170	GO:0007229	integrin-mediated signaling pathway	6/179	106/18862	0.000522903	0.007271433	0.005653184	COL3A1/ADAMTS1/FN1/TIMP1/ITGA7/NRP1	6
	171	GO:0071774	response to fibroblast growth factor	7/179	148/18862	0.000535801	0.007407212	0.005758746	COL1A1/THBS1/SMOC2/FGF7/SULF1/NR4A1/CCL2	7
	172	GO:0071634	regulation of transforming growth factor beta production	4/179	40/18862	0.000548912	0.00750074	0.00583146	FN1/THBS1/LUM/LTBP1	4
	173	GO:1904037	positive regulation of epithelial cell apoptotic process	4/179	40/18862	0.000548912	0.00750074	0.00583146	THBS1/CD248/GSN/CCL2	4
	174	GO:0051896	regulation of protein kinase B signaling	9/179	247/18862	0.000602777	0.008109909	0.006305059	PDGFRB/THBS1/IGFBP5/AXL/FGF7/RCN3/C1QTNF1 /PDGFRA/PDGFA	9
	175	GO:0001953	negative regulation of cell-matrix adhesion	4/179	41/18862	0.000603783	0.008109909	0.006305059	THBS1/DLC1/APOD/LRP1	4
	176	GO:0090279	regulation of calcium ion import	4/179	41/18862	0.000603783	0.008109909	0.006305059	PDGFRB/CXCL12/PLN/CCL2	4
	177	GO:0010631	epithelial cell migration	11/179	357/18862	0.000626556	0.008368233	0.006505893	DCN/SERPINF1/SPARC/THBS1/SMOC2/FGF7/NR4A1 /FSTL1/KANK2/NRP1/PTP4A3	11
	178	GO:0007178	transmembrane receptor protein serine/threonine kinase signaling pathway	11/179	358/18862	0.00064118	0.00851545	0.006620347	COL1A2/COL3A1/THBS1/VIM/FMOD/ASPN/SULF1 /FSTL1/SFRP2/TGFB1I1/LTBP1	11
	179	GO:0071604	transforming growth factor beta production	4/179	42/18862	0.000662436	0.008748599	0.006801609	FN1/THBS1/LUM/LTBP1	4
	180	GO:0090132	epithelium migration	11/179	360/18862	0.000671285	0.008816008	0.006854017	DCN/SERPINF1/SPARC/THBS1/SMOC2/FGF7/NR4A1 /FSTL1/KANK2/NRP1/PTP4A3	11
	181	GO:0071560	cellular response to transforming growth factor beta stimulus	9/179	251/18862	0.000676078	0.008816008	0.006854017	COL1A2/COL3A1/COL1A1/THBS1/FMOD/ASPN/ COL4A2/TGFB1I1/LTBP1	9
	182	GO:0051017	actin filament bundle assembly	7/179	154/18862	0.000678728	0.008816008	0.006854017	CALD1/TPM1/DLC1/NRP1/EPS8/SORBS1/SYNPO2	7
	183	GO:0060326	cell chemotaxis	10/179	306/18862	0.000704992	0.009107109	0.007080333	RARRES2/PDGFRB/CXCL12/THBS1/SMOC2/NR4A1 /CCL2/PDGFRA/NRP1/PGF	10
	184	GO:1904738	vascular associated smooth muscle cell migration	4/179	43/18862	0.000725013	0.009264496	0.007202694	ADAMTS1/IGFBP5/TPM1/LRP1	4
	185	GO:1904752	regulation of vascular associated smooth muscle cell migration	4/179	43/18862	0.000725013	0.009264496	0.007202694	ADAMTS1/IGFBP5/TPM1/LRP1	4
	186	GO:0045785	positive regulation of cell adhesion	12/179	425/18862	0.000771388	0.009804097	0.007622208	FN1/CXCL12/ZBTB16/CCDC80/LGALS1/SFRP2/CCL2 /TPM1/EFEMP2/NID1/NRP1/FBLN2	12
	187	GO:0030203	glycosaminoglycan metabolic process	7/179	158/18862	0.000789718	0.009902013	0.007698332	DCN/BGN/PDGFRB/FMOD/LUM/SDC2/PRELP	7
	188	GO:0061572	actin filament bundle organization	7/179	158/18862	0.000789718	0.009902013	0.007698332	CALD1/TPM1/DLC1/NRP1/EPS8/SORBS1/SYNPO2	7
	189	GO:0060324	face development	4/179	44/18862	0.000791658	0.009902013	0.007698332	CRISPLD2/COL1A1/PDGFRA/MMP2	4
	190	GO:0071559	response to transforming growth factor beta	9/179	257/18862	0.000799559	0.009948199	0.00773424	COL1A2/COL3A1/COL1A1/THBS1/FMOD/ASPN /COL4A2/TGFB1I1/LTBP1	9
	191	GO:0003206	cardiac chamber morphogenesis	6/179	115/18862	0.00080412	0.009952569	0.007737637	ADAMTS1/HEYL/SFRP2/TPM1/NRP1/CPE	6
	192	GO:0048678	response to axon injury	5/179	77/18862	0.000828037	0.010195208	0.007926277	LGALS1/LAMB2/MAP1B/APOD/LRP1	5
	193	GO:1904019	epithelial cell apoptotic process	6/179	116/18862	0.000841411	0.010306193	0.008012562	THBS1/CD248/GSN/CCL2/NUPR1/ANGPTL4	6
	194	GO:0050680	negative regulation of epithelial cell proliferation	7/179	160/18862	0.000850348	0.010313165	0.008017983	SERPINF1/SPARC/THBS1/SULF1/SFRP2/CCL2/NUPR1	7
	195	GO:0090280	positive regulation of calcium ion import	3/179	20/18862	0.000850705	0.010313165	0.008017983	PDGFRB/CXCL12/CCL2	3
	196	GO:0050772	positive regulation of axonogenesis	5/179	78/18862	0.000878042	0.010590263	0.008233413	FN1/CXCL12/MAP1B/NRP1/LRP1	5
	197	GO:0007015	actin filament organization	12/179	435/18862	0.000943232	0.011318787	0.008799805	TPM2/CXCL12/CALD1/LMOD1/GSN/TPM1/DLC1 /NRP1/EPS8/SORBS1/SYNPO2/SORBS2	12
	198	GO:1990138	neuron projection extension	7/179	164/18862	0.000982611	0.011731777	0.009120884	FN1/CXCL12/LAMB2/MAP1B/DPYSL2/NRP1/LRP1	7
	199	GO:0010811	positive regulation of cell-substrate adhesion	6/179	121/18862	0.001048296	0.012453122	0.009681695	FN1/CCDC80/EFEMP2/NID1/NRP1/FBLN2	6
	200	GO:0001656	metanephros development	5/179	82/18862	0.001100819	0.012958038	0.010074243	PDGFRB/LAMB2/ID3/PDGFRA/PDGFA	5
	201	GO:0051146	striated muscle cell differentiation	9/179	269/18862	0.001101762	0.012958038	0.010074243	PDGFRB/IGFBP5/LMOD1/TPM1/PDGFRA/SORBS2 /EHD2/MYH11/CSRP2	9
	202	GO:0060343	trabecula formation	3/179	22/18862	0.001133289	0.013262846	0.010311216	ADAMTS1/COL1A1/MMP2	3
	203	GO:0070482	response to oxygen levels	11/179	385/18862	0.001158168	0.013487242	0.010485673	PDGFRB/COL1A1/CXCL12/THBS1/SOD3/EDNRA /CRYAB/EPAS1/MMP2/PGF/ANGPTL4	11
	204	GO:0001952	regulation of cell-matrix adhesion	6/179	125/18862	0.001240253	0.014302236	0.011119291	THBS1/DLC1/EFEMP2/NRP1/APOD/LRP1	6
	205	GO:0032355	response to estradiol	6/179	125/18862	0.001240253	0.014302236	0.011119291	PDGFRB/COL1A1/GGT5/MAP1B/TFPI/CRYAB	6
	206	GO:0034109	homotypic cell-cell adhesion	5/179	85/18862	0.001293523	0.014456336	0.011239096	MYL9/FN1/LGALS1/C1QTNF1/PDGFRA	5
	207	GO:0051043	regulation of membrane protein ectodomain proteolysis	3/179	23/18862	0.001294235	0.014456336	0.011239096	TIMP3/TIMP1/TIMP2	3
	208	GO:1903055	positive regulation of extracellular matrix organization	3/179	23/18862	0.001294235	0.014456336	0.011239096	DDR2/EFEMP2/PHLDB1	3
	209	GO:1904754	positive regulation of vascular associated smooth muscle cell migration	3/179	23/18862	0.001294235	0.014456336	0.011239096	ADAMTS1/IGFBP5/LRP1	3
	210	GO:2000353	positive regulation of endothelial cell apoptotic process	3/179	23/18862	0.001294235	0.014456336	0.011239096	THBS1/CD248/CCL2	3

Table S13: biological processes of top-200 DEGs (continued 4)

Ontology	No.	ID	Description	GeneRatio	BgRatio	pvalue	p.adjust	qvalue	geneID	Count
BP	211	GO:0006022	aminoglycan metabolic process	7/179	172/18862	0.001295469	0.014456336	0.011239096	DCN/BGN/PDGFRB/FMOD/LUM/SDC2/PRELP	7
	212	GO:0048588	developmental cell growth	8/179	222/18862	0.001296423	0.014456336	0.011239096	FN1/CXCL12/LAMB2/MAP1B/DPYSL2/NRP1 /SORBS2/LRP1	8
	213	GO:0061138	morphogenesis of a branching epithelium	7/179	173/18862	0.001339497	0.014866414	0.011557912	FGF7/SULF1/SFRP2/EDNRA/COL4A1/NRP1/PDGFA	7
	214	GO:0014066	regulation of phosphatidylinositol 3-kinase signaling	6/179	127/18862	0.001345775	0.014866414	0.011557912	DCN/PDGFRB/FN1/PDGFRA/IER3/PDGFA	6
	215	GO:0032964	collagen biosynthetic process	4/179	51/18862	0.001384143	0.015150545	0.01177881	PDGFRB/COL1A1/VIM/RCN3	4
	216	GO:0043542	endothelial cell migration	9/179	278/18862	0.001384314	0.015150545	0.01177881	DCN/SERPINF1/SPARC/THBS1/SMOC2/NR4A1 /FSTL1/NRP1/PTP4A3	9
	217	GO:0060560	developmental growth involved in morphogenesis	8/179	225/18862	0.00141172	0.01537929	0.011956648	FN1/CXCL12/LAMB2/MAP1B/SFRP2/DPYSL2 /NRP1/LRP1	8
	218	GO:0031103	axon regeneration	4/179	52/18862	0.001488504	0.016141388	0.012549142	LAMB2/MAP1B/APOD/LRP1	4
	219	GO:0033273	response to vitamin	5/179	89/18862	0.001587852	0.017140096	0.013325589	COL1A1/SPARC/MAP1B/GSN/KANK2	5
	220	GO:0072012	glomerulus vasculature development	3/179	25/18862	0.001657587	0.017811527	0.013847594	ACTA2/PDGFRB/PDGFRA	3
	221	GO:0014068	positive regulation of phosphatidylinositol 3-kinase signaling	5/179	90/18862	0.001668536	0.017848052	0.01387599	DCN/PDGFRB/FN1/PDGFRA/PDGFA	5
	222	GO:0062197	cellular response to chemical stress	10/179	347/18862	0.001811219	0.01928703	0.014994725	GPX3/SOD3/AXL/EFHD1/TPM1/PDGFRA/MYLK /FBLN5/EPAS1/MMP2	10
	223	GO:0035904	aorta development	4/179	55/18862	0.001834306	0.019445286	0.015117761	PDGFRB/EFEMP2/MYLK/LRP1	4
	224	GO:0061437	renal system vasculature development	3/179	26/18862	0.001860805	0.019550855	0.015199837	ACTA2/PDGFRB/PDGFRA	3
	225	GO:0061440	kidney vasculature development	3/179	26/18862	0.001860805	0.019550855	0.015199837	ACTA2/PDGFRB/PDGFRA	3
	226	GO:0045598	regulation of fat cell differentiation	6/179	136/18862	0.001907866	0.019956621	0.0155153	RARRES2/ZBTB16/FRZB/SFRP2/TGFB1I1/ADIRF	6
	227	GO:0050764	regulation of phagocytosis	5/179	94/18862	0.002021627	0.021000656	0.016326986	C4B/C4A/MFGE8/CCL2/LRP1	5
	228	GO:0001763	morphogenesis of a branching structure	7/179	186/18862	0.002025444	0.021000656	0.016326986	FGF7/SULF1/SFRP2/EDNRA/COL4A1/NRP1/PDGFA	7
	229	GO:0090287	regulation of cellular response to growth factor stimulus	9/179	296/18862	0.002124199	0.02192841	0.01704827	DCN/THBS1/SMOC2/ASPN/SULF1/FSTL1/ SFRP2/TGFB1I1/LTBP1	9
	230	GO:0048661	positive regulation of smooth muscle cell proliferation	5/179	96/18862	0.002217298	0.022789965	0.017718087	PDGFRB/ADAMTS1/THBS1/IGFBP5/MMP2	5
	231	GO:2000351	regulation of endothelial cell apoptotic process	4/179	58/18862	0.002232154	0.022843338	0.017759581	THBS1/CD248/CCL2/ANGPTL4	4
	232	GO:0046578	regulation of Ras protein signal transduction	7/179	190/18862	0.002283465	0.023189716	0.018028874	COL3A1/PDGFRB/TIMP2/KANK2/DLC1/NRP1 /EPS8	7
	233	GO:0090257	regulation of muscle system process	8/179	243/18862	0.002285619	0.023189716	0.018028874	MYL9/IGFBP5/PLN/HSPB6/CNN1/CASQ2/ SGCA/TPM1	8
	234	GO:0018146	keratan sulfate biosynthetic process	3/179	28/18862	0.002312242	0.023339669	0.018145455	FMOD/LUM/PRELP	3
	235	GO:0060191	regulation of lipase activity	5/179	97/18862	0.002320145	0.023339669	0.018145455	PDGFRB/EDNRA/PDGFRA/LRP1/ANGPTL4	5
	236	GO:0045926	negative regulation of growth	8/179	245/18862	0.00240432	0.024083951	0.018724097	IGFBP5/FRZB/SFRP2/FHL1/CRYAB/NRP1/ MT1M/MEG3	8
	237	GO:0048015	phosphatidylinositol-mediated signaling	7/179	192/18862	0.002421633	0.024155024	0.018779354	DCN/PDGFRB/FN1/GSN/PDGFRA/IER3/PDGFA	7
	238	GO:0042692	muscle cell differentiation	10/179	362/18862	0.002463472	0.024469111	0.01902354	PDGFRB/IGFBP5/LMOD1/TPM1/PDGFRA/ EFEMP2/SORBS2/EHD2/MYH11/CSRP2	10
	239	GO:0044272	sulfur compound biosynthetic process	7/179	193/18862	0.002493089	0.02465968	0.019171699	DCN/BGN/GGT5/FMOD/PDK4/LUM/PRELP	7
	240	GO:0060041	retina development in camera-type eye	6/179	145/18862	0.002632037	0.02592556	0.020155859	PDGFRB/SERPINF1/LAMB2/PDGFRA/COL4A1 /NRP1	6
	241	GO:0010975	regulation of neuron projection development	11/179	428/18862	0.002656753	0.026060435	0.020260717	SERPINF1/FN1/CXCL12/PRRX1/VIM/LGALS1/ MAP1B/SFRP2/SDC2/NRP1/LRP1	11
	242	GO:0048017	inositol lipid-mediated signaling	7/179	196/18862	0.002717234	0.02654356	0.020636323	DCN/PDGFRB/FN1/GSN/PDGFRA/IER3/PDGFA	7
	243	GO:0010667	negative regulation of cardiac muscle cell apoptotic process	3/179	30/18862	0.00282607	0.027493125	0.021374564	HSPB6/SFRP2/NUPR1	3
	244	GO:0042698	ovulation cycle	4/179	62/18862	0.002850065	0.027500221	0.021380081	ADAMTS1/SERPINF1/AXL/PDGFRA	4
	245	GO:0070527	platelet aggregation	4/179	62/18862	0.002850065	0.027500221	0.021380081	MYL9/FN1/C1QTNF1/PDGFRA	4
	246	GO:0043434	response to peptide hormone	11/179	435/18862	0.003006909	0.02882307	0.022408532	RARRES2/COL1A1/SPARC/CXCL12/IGFBP5/PLN /TIMP1/MAP1B/PDK4/NR4A1/SORBS1	11
	247	GO:0031532	actin cytoskeleton reorganization	5/179	103/18862	0.003011548	0.02882307	0.022408532	FGF7/GSN/PDGFRA/ANTXR1/NRP1	5
	248	GO:0010952	positive regulation of peptidase activity	7/179	200/18862	0.003039809	0.02897624	0.022527614	PCOLCE/FN1/GSN/RCN3/SFRP2/DLC1/ANTXR1	7
	249	GO:0072577	endothelial cell apoptotic process	4/179	64/18862	0.003198952	0.03007039	0.023378262	THBS1/CD248/CCL2/ANGPTL4	4
	250	GO:0007179	transforming growth factor beta receptor signaling pathway	7/179	202/18862	0.003211701	0.03007039	0.023378262	COL1A2/COL3A1/THBS1/FMOD/ASPN/ TGFB1I1/LTBP1	7
	251	GO:0009612	response to mechanical stimulus	7/179	202/18862	0.003211701	0.03007039	0.023378262	DCN/COL3A1/COL1A1/CXCL12/THBS1/ MAP1B/GSN	7
	252	GO:0003205	cardiac chamber development	6/179	151/18862	0.003218193	0.03007039	0.023378262	ADAMTS1/HEYL/SFRP2/TPM1/NRP1/CPE	6
	253	GO:0008360	regulation of cell shape	6/179	151/18862	0.003218193	0.03007039	0.023378262	FN1/ITGA7/CCL2/TPM1/DLC1/EPS8	6
	254	GO:1902905	positive regulation of supramolecular fiber organization	7/179	203/18862	0.003300385	0.030716968	0.023880945	LMOD1/MAP1B/GSN/TPM1/EFEMP2/NRP1 /SYNPO2	7
	255	GO:0010664	negative regulation of striated muscle cell apoptotic process	3/179	32/18862	0.003404956	0.031565944	0.024540982	HSPB6/SFRP2/NUPR1	3
	256	GO:0006959	humoral immune response	10/179	380/18862	0.003482236	0.032153535	0.024997806	A2M/RARRES2/MFAP4/SERPING1/C1R/C4B /C4A/C1S/CCL2/CFH	10
	257	GO:0051056	regulation of small GTPase mediated signal transduction	9/179	319/18862	0.003495541	0.032153535	0.024997806	COL3A1/A2M/PDGFRB/TIMP2/ARHGEF17 /KANK2/DLC1/NRP1/EPS8	9
	258	GO:0014065	phosphatidylinositol 3-kinase signaling	6/179	154/18862	0.003545369	0.032485476	0.025255873	DCN/PDGFRB/FN1/PDGFRA/IER3/PDGFA	6
	259	GO:0034446	substrate adhesion-dependent cell spreading	5/179	108/18862	0.003692327	0.033689897	0.026192252	FN1/AXL/LAMB2/ANTXR1/NRP1	5
	260	GO:0042339	keratan sulfate metabolic process	3/179	33/18862	0.00371957	0.033689897	0.026192252	FMOD/LUM/PRELP	3
	261	GO:1901020	negative regulation of calcium ion transmembrane transporter activity	3/179	33/18862	0.00371957	0.033689897	0.026192252	PLN/GEM/CASQ2	3
	262	GO:0014820	tonic smooth muscle contraction	2/179	10/18862	0.003833403	0.034456897	0.026788558	EDNRA/MYLK	2
	263	GO:0032060	bleb assembly	2/179	10/18862	0.003833403	0.034456897	0.026788558	PMP22/MYLK	2
	264	GO:0010543	regulation of platelet activation	3/179	34/18862	0.004051351	0.036278006	0.028204381	C1QTNF1/PDGFRA/PDGFA	3
	265	GO:0034614	cellular response to reactive oxygen species	6/179	159/18862	0.004144791	0.036883283	0.028674955	SOD3/AXL/TPM1/PDGFRA/FBLN5/MMP2	6
	266	GO:0071675	regulation of mononuclear cell migration	5/179	111/18862	0.004150149	0.036883283	0.028674955	RARRES2/CXCL12/THBS1/CCL2/APOD	5
	267	GO:0003208	cardiac ventricle morphogenesis	4/179	69/18862	0.004196126	0.03715222	0.02888404	HEYL/SFRP2/TPM1/CPE	4
	268	GO:0021782	glial cell development	5/179	112/18862	0.004311378	0.037704621	0.029313504	MXRA8/VIM/LAMB2/GSN/LRP1	5
	269	GO:0071214	cellular response to abiotic stimulus	9/179	330/18862	0.00435968	0.037704621	0.029313504	MFAP4/COL1A1/TIMP1/SFRP2/CRYAB /EFHD1/ADIRF/MYLK/MMP2	9
	270	GO:0104004	cellular response to environmental stimulus	9/179	330/18862	0.00435968	0.037704621	0.029313504	MFAP4/COL1A1/TIMP1/SFRP2/CRYAB /EFHD1/ADIRF/MYLK/MMP2	9

Table S14: biological processes of top-200 DEGs (continued 5)

Ontology	No.	ID	Description	GeneRatio	BgRatio	pvalue	p.adjust	qvalue	geneID	Count
BP	271	GO:0040036	regulation of fibroblast growth factor receptor signaling pathway	3/179	35/18862	0.004400573	0.037704621	0.029313504	THBS1/SMOC2/SULF1	3
	272	GO:0006937	regulation of muscle contraction	6/179	161/18862	0.004404416	0.037704621	0.029313504	MYL9/PLN/HSPB6/CNN1/CASQ2/TPM1	6
	273	GO:0009408	response to heat	6/179	161/18862	0.004404416	0.037704621	0.029313504	CXCL12/IGFBP7/THBS1/TGFB1I1/CRYAB /HSPB8	6
	274	GO:0030307	positive regulation of cell growth	6/179	161/18862	0.004404416	0.037704621	0.029313504	FN1/CXCL12/MAP1B/SFRP2/NRP1/LRP1	6
	275	GO:1902904	negative regulation of supramolecular fiber organization	6/179	161/18862	0.004404416	0.037704621	0.029313504	LMOD1/MAP1B/GSN/CRYAB/DLC1/EPS8	6
	276	GO:0010517	regulation of phospholipase activity	4/179	70/18862	0.004418012	0.037704621	0.029313504	PDGFRB/EDNRA/PDGFRA/LRP1	4
	277	GO:1901880	negative regulation of protein depolymerization	4/179	70/18862	0.004418012	0.037704621	0.029313504	LMOD1/MAP1B/GSN/EPS8	4
	278	GO:0032102	negative regulation of response to external stimulus	10/179	394/18862	0.004486034	0.038147428	0.029657765	A2M/SERPING1/SERPINF1/THBS1/ C1QTNF1/TFPI/CCL2/PDGFRA/NRP1/PDGFA	10
	279	GO:0110053	regulation of actin filament organization	8/179	273/18862	0.004630243	0.038621391	0.030026248	CXCL12/LMOD1/GSN/TPM1/DLC1/ NRP1/EPS8/SYNPO2	8
	280	GO:0043129	surfactant homeostasis	2/179	11/18862	0.004656132	0.038621391	0.030026248	RCN3/EPAS1	2
	281	GO:0043589	skin morphogenesis	2/179	11/18862	0.004656132	0.038621391	0.030026248	COL1A2/COL1A1	2
	282	GO:0061299	retina vasculature morphogenesis in camera-type eye	2/179	11/18862	0.004656132	0.038621391	0.030026248	COL4A1/NRP1	2
	283	GO:0071492	cellular response to UV-A	2/179	11/18862	0.004656132	0.038621391	0.030026248	TIMP1/MMP2	2
	284	GO:0097048	dendritic cell apoptotic process	2/179	11/18862	0.004656132	0.038621391	0.030026248	CXCL12/AXL	2
	285	GO:2000668	regulation of dendritic cell apoptotic process	2/179	11/18862	0.004656132	0.038621391	0.030026248	CXCL12/AXL	2
	286	GO:0014912	negative regulation of smooth muscle cell migration	3/179	36/18862	0.004767498	0.03940687	0.03063692	IGFBP5/TPM1/LRP1	3
	287	GO:1903510	mucopolysaccharide metabolic process	5/179	115/18862	0.004821843	0.039717202	0.030878188	DCN/BGN/FMOD/LUM/PRELP	5
	288	GO:1903036	positive regulation of response to wounding	4/179	72/18862	0.004885234	0.040099632	0.031175509	THBS1/SMOC2/MYLK/LRP1	4
	289	GO:0007584	response to nutrient	6/179	165/18862	0.004959429	0.040567786	0.031539476	COL1A1/SPARC/MAP1B/GSN/SFRP2 /KANK2	6
	290	GO:0042246	tissue regeneration	4/179	73/18862	0.005130815	0.041824989	0.03251689	MUSTN1/GSN/SGCA/APOD	4
	291	GO:0009743	response to carbohydrate	7/179	221/18862	0.005239176	0.042561552	0.033089531	SERPINF1/SPARC/COL6A2/THBS1/LGALS1 /MAP1B/LRP1	7
	292	GO:0048608	reproductive structure development	10/179	405/18862	0.005424614	0.043436547	0.033769797	DCN/PDGFRB/ADAMTS1/SERPINF1/AXL /SULF1/SFRP2/NUPR1/PDGFRA/EPAS1	10
	293	GO:0010642	negative regulation of platelet-derived growth factor receptor signaling pathway	2/179	12/18862	0.005552637	0.043436547	0.033769797	APOD/LRP1	2
	294	GO:0030208	dermatan sulfate biosynthetic process	2/179	12/18862	0.005552637	0.043436547	0.033769797	DCN/BGN	2
	295	GO:0032908	regulation of transforming growth factor beta1 production	2/179	12/18862	0.005552637	0.043436547	0.033769797	THBS1/LUM	2
	296	GO:0042308	negative regulation of protein import into nucleus	2/179	12/18862	0.005552637	0.043436547	0.033769797	PKIG/APOD	2
	297	GO:0061430	bone trabecula morphogenesis	2/179	12/18862	0.005552637	0.043436547	0.033769797	COL1A1/MMP2	2
	298	GO:0061469	regulation of type B pancreatic cell proliferation	2/179	12/18862	0.005552637	0.043436547	0.033769797	NR4A1/NUPR1	2
	299	GO:1904590	negative regulation of protein import	2/179	12/18862	0.005552637	0.043436547	0.033769797	PKIG/APOD	2
	300	GO:0010463	mesenchymal cell proliferation	3/179	38/18862	0.00555544	0.043436547	0.033769797	PRRX1/FGF7/PDGFA	3
	301	GO:0048246	macrophage chemotaxis	3/179	38/18862	0.00555544	0.043436547	0.033769797	RARRES2/THBS1/CCL2	3
	302	GO:1904706	negative regulation of vascular associated smooth muscle cell proliferation	3/179	38/18862	0.00555544	0.043436547	0.033769797	CNN1/TPM1/EFEMP2	3
	303	GO:0002688	regulation of leukocyte chemotaxis	5/179	119/18862	0.005567375	0.043436547	0.033769797	RARRES2/CXCL12/THBS1/CCL2/PGF	5
	304	GO:0001937	negative regulation of endothelial cell proliferation	4/179	75/18862	0.005646513	0.043909068	0.03413716	SPARC/THBS1/SULF1/CCL2	4
	305	GO:0061458	reproductive system development	10/179	408/18862	0.005705574	0.044222878	0.034381132	DCN/PDGFRB/ADAMTS1/SERPINF1/AXL /SULF1/SFRP2/NUPR1/PDGFRA/EPAS1	10
	306	GO:0070252	actin-mediated cell contraction	5/179	120/18862	0.005765778	0.044543465	0.034630372	TPM2/PLN/VIM/GSN/TPM1	5
	307	GO:0030049	muscle filament sliding	3/179	39/18862	0.00597692	0.045578832	0.035435319	TPM2/VIM/TPM1	3
	308	GO:0033275	actin-myosin filament sliding	3/179	39/18862	0.00597692	0.045578832	0.035435319	TPM2/VIM/TPM1	3
	309	GO:0072210	metanephric nephron development	3/179	39/18862	0.00597692	0.045578832	0.035435319	PDGFRB/LAMB2/PDGFRA	3
	310	GO:1903170	negative regulation of calcium ion transmembrane transport	3/179	39/18862	0.00597692	0.045578832	0.035435319	PLN/GEM/CASQ2	3
	311	GO:0001666	response to hypoxia	9/179	348/18862	0.00612423	0.046552023	0.036191928	CXCL12/THBS1/SOD3/EDNRA/CRYAB /EPAS1/MMP2/PGF/ANGPTL4	9
	312	GO:0055013	cardiac muscle cell development	4/179	77/18862	0.006195704	0.046944375	0.036496963	PDGFRB/PDGFRA/SORBS2/MYH11	4
	313	GO:0008361	regulation of cell size	6/179	173/18862	0.006221308	0.046987768	0.036530699	FN1/CXCL12/MAP1B/DPYSL2/NRP1/LRP1	6
	314	GO:0042476	odontogenesis	5/179	123/18862	0.006390859	0.048025512	0.037337494	COL1A2/COL1A1/ASPN/ID3/PDGFRA	5
	315	GO:0014706	striated muscle tissue development	9/179	351/18862	0.006465296	0.048025512	0.037337494	DCN/PDGFRB/PLN/HEYL/NUPR1/ TPM1/PDGFRA/SORBS2/MYH11	9
	316	GO:0002921	negative regulation of humoral immune response	2/179	13/18862	0.006521459	0.048025512	0.037337494	A2M/SERPING1	2
	317	GO:0030205	dermatan sulfate metabolic process	2/179	13/18862	0.006521459	0.048025512	0.037337494	DCN/BGN	2
	318	GO:0032905	transforming growth factor beta1 production	2/179	13/18862	0.006521459	0.048025512	0.037337494	THBS1/LUM	2
	319	GO:0043568	positive regulation of insulin-like growth factor receptor signaling pathway	2/179	13/18862	0.006521459	0.048025512	0.037337494	IGFBP5/IGFBP4	2
	320	GO:0048875	chemical homeostasis within a tissue	2/179	13/18862	0.006521459	0.048025512	0.037337494	RCN3/EPAS1	2
	321	GO:1902043	positive regulation of extrinsic apoptotic signaling pathway via death domain receptors	2/179	13/18862	0.006521459	0.048025512	0.037337494	THBS1/TIMP3	2
	322	GO:0045766	positive regulation of angiogenesis	6/179	175/18862	0.006570245	0.048025512	0.037337494	THBS1/HSPB6/SMOC2/SFRP2/PGF/ANGPTL4	6
	323	GO:1904018	positive regulation of vasculature development	6/179	175/18862	0.006570245	0.048025512	0.037337494	THBS1/HSPB6/SMOC2/SFRP2/PGF/ANGPTL4	6
	324	GO:0010038	response to metal ion	9/179	352/18862	0.006582177	0.048025512	0.037337494	SERPINF1/SPARC/THBS1/SOD3/PLN /GSN/CASQ2/CYBRD1/MT1M	9

Table S15: cellular components of top-200 DEGs

Ontology	No.	ID	Description	GeneRatio	BgRatio	pvalue	p.adjust	qvalue	geneID	Count
CC	1	GO:0062023	collagen-containing extracellular matrix	61/183	423/19520	9E-56	1.71E-53	1.3E-53	DCN/COL1A2/COL3A1/SPARCL1/A2M/RARRES2/MFAP4/PCOLCE/BGN/ADAMTS1/SERPING1/COL1A1/SERPINF1/MGP/SPARC/FN1/AEBP1/CXCL12/COL6A2/IGFBP7/COL6A1/COL14A1/COL6A3/THBS1/SOD3/MFGE8/TIMP3/CCDC80/TIMP1/ANGPTL2/FMOD/LAMA4/LGALS1/LAMB2/SMOC2/ASPN/SULF1/PODN/TIMP2/COL4A2/COL5A2/SFRP2/ANGPTL1/TGFB1I1/LUM/DPT/LTBP1/COL4A1/EFEMP2/SDC2/NID1/COL12A1/ABI3BP/FBLN5/THBS2/PRELP/FBLN2/ADAMTS4/MMP2/SRPX/ANGPTL4	61
	2	GO:0005788	endoplasmic reticulum lumen	29/183	306/19520	6.24E-21	5.93E-19	4.5E-19	COL1A2/COL3A1/SPARCL1/COL1A1/FN1/MXRA8/COL6A2/IGFBP7/COL6A1/COL14A1/COL6A3/C4A/THBS1/IGFBP5/MFGE8/TIMP1/IGFBP4/LGALS1/LAMB2/RCN3/COL4A2/COL5A2/FSTL1/CASQ2/LTBP1/COL4A1/SDC2/COL12A1/PDGFA	29
	3	GO:0005581	collagen trimer	14/183	87/19520	7.31E-14	4.63E-12	3.51E-12	DCN/COL1A2/COL3A1/COL1A1/COL6A2/COL6A1/COL14A1/COL6A3/COL4A2/COL5A2/C1QTNF1/LUM/COL4A1/COL12A1	14
	4	GO:0005604	basement membrane	13/183	94/19520	4.37E-12	2.08E-10	1.58E-10	ADAMTS1/SERPINF1/SPARC/FN1/CCDC80/TIMP1/LAMA4/LAMB2/SMOC2/COL4A2/COL4A1/EFEMP2/NID1	13
	5	GO:0043292	contractile fiber	16/183	231/19520	5.89E-10	1.87E-08	1.42E-08	ACTA2/MYL9/TPM2/CALD1/LMOD1/FHL5/CASQ2/CRYAB/TPM1/NEXN/PDLIM3/SYNPO2/SORBS2/MMP2/MYH11/CSRP2	16
	6	GO:0098644	complex of collagen trimers	7/183	21/19520	5.9E-10	1.87E-08	1.42E-08	COL1A2/COL3A1/COL1A1/COL4A2/COL5A2/LUM/COL4A1	7
	7	GO:0030016	myofibril	14/183	224/19520	2.69E-08	7.3E-07	5.54E-07	MYL9/TPM2/CALD1/LMOD1/FHL5/CASQ2/CRYAB/TPM1/NEXN/PDLIM3/SYNPO2/SORBS2/MMP2/CSRP2	14
	8	GO:0005583	fibrillar collagen trimer	5/183	12/19520	5.15E-08	1.09E-06	8.25E-07	COL1A2/COL3A1/COL1A1/COL5A2/LUM	5
	9	GO:0098643	banded collagen fibril	5/183	12/19520	5.15E-08	1.09E-06	8.25E-07	COL1A2/COL3A1/COL1A1/COL5A2/LUM	5
	10	GO:0030017	sarcomere	13/183	203/19520	6.46E-08	1.23E-06	9.31E-07	MYL9/TPM2/LMOD1/FHL5/CASQ2/CRYAB/TPM1/NEXN/PDLIM3/SYNPO2/SORBS2/MMP2/CSRP2	13
	11	GO:0005925	focal adhesion	18/183	416/19520	7.97E-08	1.38E-06	1.04E-06	PDGFRB/TNS1/CNN3/VIM/DDR2/GSN/CNN1/FHL1/TGFB1I1/DLC1/NEXN/NRP1/SORBS1/SYNPO2/SORBS2/LRP1/CSRP2/MCAM	18
	12	GO:0030055	cell-substrate junction	18/183	423/19520	1.02E-07	1.62E-06	1.23E-06	PDGFRB/TNS1/CNN3/VIM/DDR2/GSN/CNN1/FHL1/TGFB1I1/DLC1/NEXN/NRP1/SORBS1/SYNPO2/SORBS2/LRP1/CSRP2/MCAM	18
	13	GO:0031091	platelet alpha granule	9/183	91/19520	1.86E-07	2.72E-06	2.06E-06	A2M/SERPING1/ISLR/SPARC/FN1/THBS1/TIMP1/THBS2/PDGFA	9
	14	GO:0031093	platelet alpha granule lumen	8/183	67/19520	2.08E-07	2.83E-06	2.15E-06	A2M/SERPING1/ISLR/SPARC/FN1/THBS1/TIMP1/PDGFA	8
	15	GO:0072562	blood microparticle	10/183	146/19520	1.21E-06	1.53E-05	1.16E-05	A2M/SERPING1/FN1/C1R/C4B/C4A/C1S/GSN/CFH/ANGPTL4	10
	16	GO:0030018	Z disc	9/183	122/19520	2.26E-06	2.68E-05	2.03E-05	MYL9/FHL5/CASQ2/CRYAB/NEXN/PDLIM3/SYNPO2/SORBS2/CSRP2	9
	17	GO:0043202	lysosomal lumen	8/183	96/19520	3.36E-06	3.76E-05	2.85E-05	DCN/BGN/PDGFRB/FMOD/CTSK/LUM/SDC2/PRELP	8
	18	GO:0031674	I band	9/183	134/19520	4.9E-06	5.17E-05	3.93E-05	MYL9/FHL5/CASQ2/CRYAB/NEXN/PDLIM3/SYNPO2/SORBS2/CSRP2	9
	19	GO:0005796	Golgi lumen	8/183	102/19520	5.3E-06	5.21E-05	3.96E-05	DCN/BGN/SOD3/FMOD/LUM/SDC2/PRELP/PDGFA	8
	20	GO:0032432	actin filament bundle	7/183	73/19520	5.49E-06	5.21E-05	3.96E-05	MYL9/CRYAB/TPM1/MYLK/SORBS1/PDLIM3/SYNPO2	7
	21	GO:0034774	secretory granule lumen	13/183	322/19520	1.14E-05	0.000104	7.86E-05	A2M/CRISPLD2/RARRES2/SERPING1/ISLR/SPARC/FN1/THBS1/TIMP3/TIMP1/GSN/TIMP2/PDGFA	13
	22	GO:0060205	cytoplasmic vesicle lumen	13/183	326/19520	1.3E-05	0.000113	8.55E-05	A2M/CRISPLD2/RARRES2/SERPING1/ISLR/SPARC/FN1/THBS1/TIMP3/TIMP1/GSN/TIMP2/PDGFA	13
	23	GO:0031983	vesicle lumen	13/183	328/19520	1.39E-05	0.000115	8.73E-05	A2M/CRISPLD2/RARRES2/SERPING1/ISLR/SPARC/FN1/THBS1/TIMP3/TIMP1/GSN/TIMP2/PDGFA	13
	24	GO:0001725	stress fiber	6/183	64/19520	2.99E-05	0.000227	0.000172	MYL9/TPM1/MYLK/SORBS1/PDLIM3/SYNPO2	6
	25	GO:0097517	contractile actin filament bundle	6/183	64/19520	2.99E-05	0.000227	0.000172	MYL9/TPM1/MYLK/SORBS1/PDLIM3/SYNPO2	6
	26	GO:0042641	actomyosin	6/183	74/19520	6.83E-05	0.000499	0.000379	MYL9/TPM1/MYLK/SORBS1/PDLIM3/SYNPO2	6
	27	GO:0001527	microfibril	3/183	12/19520	0.000168	0.001179	0.000895	MFAP4/LTBP1/EFEMP2	3
	28	GO:0005775	vacuolar lumen	8/183	173/19520	0.000232	0.001576	0.001196	DCN/BGN/PDGFRB/FMOD/CTSK/LUM/SDC2/PRELP	8
	29	GO:0016528	sarcoplasm	5/183	79/19520	0.000882	0.005779	0.004386	THBS1/PLN/GSN/CASQ2/RASD1	5
	30	GO:0005865	striated muscle thin filament	3/183	22/19520	0.001095	0.006933	0.005262	TPM2/LMOD1/TPM1	3
	31	GO:0042383	sarcolemma	6/183	134/19520	0.001667	0.010217	0.007755	BGN/COL6A2/COL6A1/COL6A3/SGCA/SGCE	6
	32	GO:0036379	myofilament	3/183	26/19520	0.001798	0.010676	0.008103	TPM2/LMOD1/TPM1	3
	33	GO:0016529	sarcoplasmic reticulum	4/183	70/19520	0.004235	0.024382	0.018506	THBS1/PLN/CASQ2/RASD1	4
	34	GO:0031252	cell leading edge	10/183	411/19520	0.005534	0.030927	0.023473	ACTA2/VIM/GSN/TPM1/DLC1/MYLK/ANTXR1/EPS8/SORBS2/SGCE	10
	35	GO:0031089	platelet dense granule lumen	2/183	14/19520	0.007387	0.040098	0.030435	RARRES2/TIMP3	2
	36	GO:0005859	muscle myosin complex	2/183	15/19520	0.008471	0.044706	0.033932	MYL9/MYH11	2

Table S16: molecular functions of top-200 DEGs

Ontology	No.	ID	Description	GeneRatio	BgRatio	pvalue	p.adjust	qvalue	geneID	Count
MF	1	GO:0005201	extracellular matrix structural constituent	38/177	170/18337	1.73032E-41	4.51614E-39	3.78849E-39	DCN/COL1A2/COL3A1/MFAP4/PCOLCE/BGN/COL1A1/MGP/SPARC/FN1/AEBP1/COL6A2/IGFBP7/COL6A1/COL14A1/COL6A3/THBS1/MFGE8/FMOD/LAMA4/LAMB2/ASPN/PODN/COL4A2/COL5A2/LUM/DPT/LTBP1/COL4A1/EFEMP2/NID1/COL12A1/ABI3BP/FBLN5/THBS2/PRELP/FBLN2/SRPX	38
	2	GO:0005518	collagen binding	19/177	68/18337	4.2397E-23	5.53281E-21	4.64136E-21	DCN/SPARCL1/PCOLCE/SPARC/FN1/AEBP1/COL6A2/COL6A1/COL14A1/THBS1/ASPN/DDR2/PODN/C1QTNF1/CTSK/LUM/NID1/ANTXR1/PDGFA	19
	3	GO:0048407	platelet-derived growth factor binding	8/177	11/18337	1.03488E-14	9.0035E-13	7.55284E-13	COL1A2/COL3A1/PDGFRB/COL1A1/COL6A1/PDGFRA/COL4A1/PDGFA	8
	4	GO:0030020	extracellular matrix structural constituent conferring tensile strength	11/177	41/18337	1.21812E-13	7.94823E-12	6.6676E-12	COL1A2/COL3A1/COL1A1/COL6A2/COL6A1/COL14A1/COL6A3/COL4A2/COL5A2/COL4A1/COL12A1	11
	5	GO:0005539	glycosaminoglycan binding	19/177	228/18337	8.10754E-13	4.23214E-11	3.55025E-11	DCN/CRISPLD2/PCOLCE/BGN/ADAMTS1/FN1/THBS1/SOD3/CCDC80/SMOC2/FGF7/SULF1/FSTL1/EFEMP2/NRP1/THBS2/PRELP/CFH/PGF	19
	6	GO:0019838	growth factor binding	15/177	137/18337	4.51601E-12	1.90201E-10	1.59556E-10	COL1A2/COL3A1/A2M/PDGFRB/COL1A1/IGFBP7/COL6A1/THBS1/IGFBP5/IGFBP4/PDGFRA/LTBP1/COL4A1/NRP1/PDGFA	15
	7	GO:0008201	heparin binding	16/177	164/18337	5.10119E-12	1.90201E-10	1.59556E-10	CRISPLD2/PCOLCE/ADAMTS1/FN1/THBS1/SOD3/CCDC80/SMOC2/FGF7/FSTL1/EFEMP2/NRP1/THBS2/PRELP/CFH/PGF	16
	8	GO:0050840	extracellular matrix binding	10/177	55/18337	1.09316E-10	3.56645E-09	2.99182E-09	DCN/SPARCL1/BGN/SPARC/THBS1/CD248/LGALS1/SMOC2/NID1/FBLN2	10
	9	GO:1901681	sulfur compound binding	17/177	260/18337	6.19891E-10	1.79768E-08	1.50804E-08	CRISPLD2/PCOLCE/ADAMTS1/FN1/THBS1/SOD3/CCDC80/SMOC2/FGF7/FSTL1/EFEMP2/NRP1/THBS2/PRELP/LRP1/CFH/PGF	17
	10	GO:0030021	extracellular matrix structural constituent conferring compression resistance	7/177	22/18337	1.04624E-09	2.73068E-08	2.29071E-08	DCN/BGN/FMOD/ASPN/PODN/LUM/PRELP	7
	11	GO:0008307	structural constituent of muscle	7/177	42/18337	1.40663E-07	3.33755E-06	2.7998E-06	MYL9/TPM2/TPM1/NEXN/SORBS2/MYH11/CSRP2	7
	12	GO:0003779	actin binding	18/177	439/18337	2.6643E-07	5.79485E-06	4.86118E-06	TAGLN/TPM2/CALD1/TNS1/CNN3/LMOD1/MAP1B/GSN/CNN1/TPM1/MYLK/ANTXR1/NEXN/EPS8/SORBS1/PDLIM3/SYNPO2/MYH11	18
	13	GO:0061134	peptidase regulator activity	13/177	230/18337	3.77999E-07	7.58905E-06	6.3663E-06	A2M/PCOLCE/SERPING1/SERPINF1/FN1/C4B/COL6A3/C4A/TIMP3/TIMP1/TIMP2/TFPI/SFRP2	13
	14	GO:0002020	protease binding	10/177	131/18337	5.73573E-07	1.0693E-05	8.97016E-06	COL1A2/COL3A1/A2M/COL1A1/FN1/TIMP3/TIMP1/TIMP2/LRP1/ADAMTS4	10
	15	GO:0043394	proteoglycan binding	6/177	36/18337	1.13807E-06	1.98024E-05	1.66118E-05	FN1/THBS1/CTSK/NID1/LRP1/CFH	6
	16	GO:0061135	endopeptidase regulator activity	11/177	192/18337	2.68202E-06	4.37504E-05	3.67013E-05	A2M/SERPING1/SERPINF1/C4B/COL6A3/C4A/TIMP3/TIMP1/TIMP2/TFPI/SFRP2	11
	17	GO:0001968	fibronectin binding	5/177	28/18337	6.50025E-06	9.9798E-05	8.37184E-05	THBS1/IGFBP5/CCDC80/CTSK/SFRP2	5
	18	GO:0005178	integrin binding	9/177	142/18337	9.86565E-06	0.000138855	0.000116482	COL3A1/FN1/CXCL12/THBS1/MFGE8/LAMB2/TIMP2/SFRP2/FBLN5	9
	19	GO:0004866	endopeptidase inhibitor activity	10/177	180/18337	1.01082E-05	0.000138855	0.000116482	A2M/SERPING1/SERPINF1/C4B/COL6A3/C4A/TIMP3/TIMP1/TIMP2/TFPI	10
	20	GO:0019199	transmembrane receptor protein kinase activity	7/177	80/18337	1.21704E-05	0.000158823	0.000133233	PDGFRB/AXL/DDR2/PDGFRL/PDGFRA/LTBP1/NRP1	7
	21	GO:0030414	peptidase inhibitor activity	10/177	187/18337	1.41063E-05	0.000175321	0.000147073	A2M/SERPING1/SERPINF1/C4B/COL6A3/C4A/TIMP3/TIMP1/TIMP2/TFPI	10
	22	GO:0004857	enzyme inhibitor activity	14/177	385/18337	2.31624E-05	0.00027479	0.000230515	A2M/SERPING1/SERPINF1/C4B/COL6A3/C4A/PLN/TIMP3/TIMP1/TIMP2/TFPI/PKIG/PPP1R14A/ANGPTL4	14
	23	GO:0004714	transmembrane receptor protein tyrosine kinase activity	6/177	61/18337	2.65717E-05	0.000301531	0.000252948	PDGFRB/AXL/DDR2/PDGFRL/PDGFRA/NRP1	6
	24	GO:0005161	platelet-derived growth factor receptor binding	3/177	15/18337	0.000369389	0.004017101	0.003369861	PDGFRB/PDGFRA/PDGFA	3
	25	GO:0008191	metalloendopeptidase inhibitor activity	3/177	16/18337	0.000451413	0.004712753	0.003953429	TIMP3/TIMP1/TIMP2	3
	26	GO:0005516	calmodulin binding	8/177	198/18337	0.00069	0.006926	0.00581	AEBP1/CALD1/CNN3/GEM/CNN1/MYLK/RGS16/MYH11	8
	27	GO:0005212	structural constituent of eye lens	3/177	23/18337	0.001358	0.013132	0.011016	HSPB6/VIM/CRYAB	3
	28	GO:0004713	protein tyrosine kinase activity	6/177	135/18337	0.001999	0.018636	0.015633	PDGFRB/AXL/DDR2/PDGFRL/PDGFRA/NRP1	6
	29	GO:0004867	serine-type endopeptidase inhibitor activity	5/177	98/18337	0.002609	0.022617	0.018973	A2M/SERPING1/SERPINF1/COL6A3/TFPI	5
	30	GO:0005520	insulin-like growth factor binding	3/177	29/18337	0.002686	0.022617	0.018973	IGFBP7/IGFBP5/IGFBP4	3
	31	GO:0043236	laminin binding	3/177	29/18337	0.002686	0.022617	0.018973	THBS1/LGALS1/NID1	3
	32	GO:0043548	phosphatidylinositol 3-kinase binding	3/177	32/18337	0.00357	0.029119	0.024427	PDGFRB/AXL/GSN	3
	33	GO:0051015	actin filament binding	7/177	208/18337	0.004132	0.032681	0.027415	TAGLN/TPM2/GSN/TPM1/ANTXR1/NEXN/MYH11	7
	34	GO:0097493	structural molecule activity conferring elasticity	2/177	11/18337	0.004812	0.036224	0.030388	FBLN5/FBLN2	2
	35	GO:0017022	myosin binding	4/177	71/18337	0.004932	0.036224	0.030388	MYL9/CALD1/AXL/GSN	4
	36	GO:0042805	actinin binding	3/177	36/18337	0.004996	0.036224	0.030388	PDLIM3/SYNPO2/CSRP2	3
	37	GO:0032036	myosin heavy chain binding	2/177	12/18337	0.005738	0.040477	0.033955	MYL9/AXL	2
	38	GO:0031994	insulin-like growth factor I binding	2/177	13/18337	0.006739	0.046284	0.038826	IGFBP5/IGFBP4	2

7 Performance comparison of DEPF and other clustering methods in identifying fibroblast cell types

To investigate whether DEPF can detect fibroblast cell types that cannot be detected by other methods, we compared our proposed DEPF with the other 13 clustering algorithms including scGAE, scDCC, GraphSCC, DEC, DCA, LWEA, ECC, ECPCS-MC, KCC, LWGP, MCLA, PTGP, and SEC on the HNSCC dataset. To construct a fair and unambiguous comparison, we first applied t-SNE to project the raw HNSCC dataset into a two-dimensional space and visualized it using true labels. After that, we applied DEPF and other 13 clustering algorithms to obtain the clustering labels for visualization in the same two-dimensional space. The experimental results are summarized in Figure S5. As depicted in this figure, we observed that DEPF detects intact fibroblast clusters, while other methods separate them. scDCC and DCA divide fibroblast clusters into two parts, while scGAE, GraphSCC, DEC, LWEA, ECC, EXPCS-MC, KCC, MCLA, PTGP, and SEC divide fibroblast clusters into three parts and LWGP incorrectly divides the fibroblast clusters into four parts. Overall, our proposed DEPF outperforms other existing approaches in identifying fibroblast cell types, further illustrating the reliability of our downstream analysis.

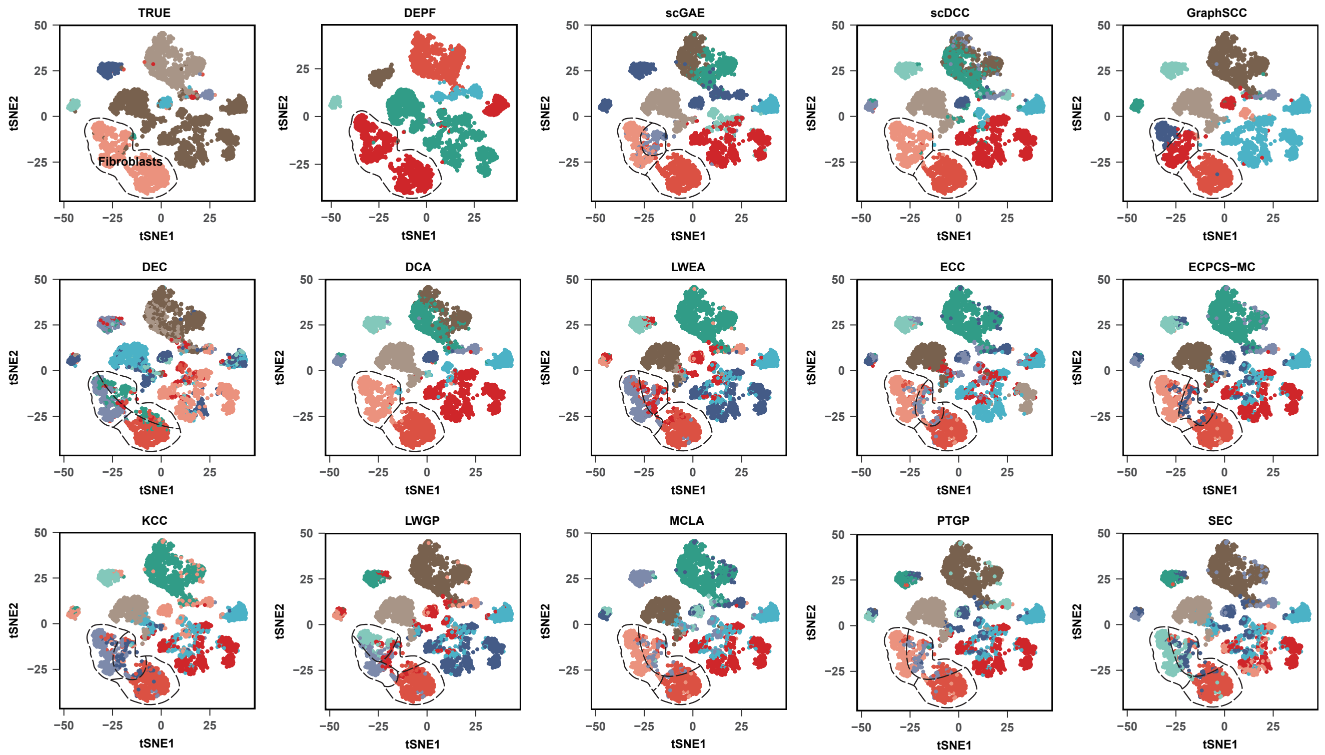

Figure S5: Color-coded representations of the HNSCC data with labels obtained from TRUE, DEPF, scGAE, scDCC, GraphSCC, DEC, DCA, LWEA, ECC, ECPCS-MC, KCC, LWGP, MCLA, PTGP and SEC, respectively.

In addition, we conducted a comparative analysis of the ability of DEPF and alternative clustering approaches to identify differentially expressed genes. To this end, we employed the Wilcoxon Rank Sum test to identify 200 differentially expressed genes for each clustering algorithm based on their predicted labels when fibroblast cells were contrasted against other cells. We subsequently performed multiple hypothesis tests using the Benjamini-Hochberg correction to adjust for the correct p values (*adj-p-values*). Finally, we computed the *adj-p-values* for those 200 differential expressed genes of each method as the calling probability of the differential genes, while those of the real differentially expressed genes, obtained from the annotated labels in the data, were treated as the true measurements. Figure S6 provides the intersection of the 200 differentially expressed genes from the truth labels with the 200 differentially expressed genes predicted from the 14 clustering algorithms. Out of the 200 differentially expressed genes predicted by DEPF, a total of 185 differentially expressed genes were confirmed to be differentially

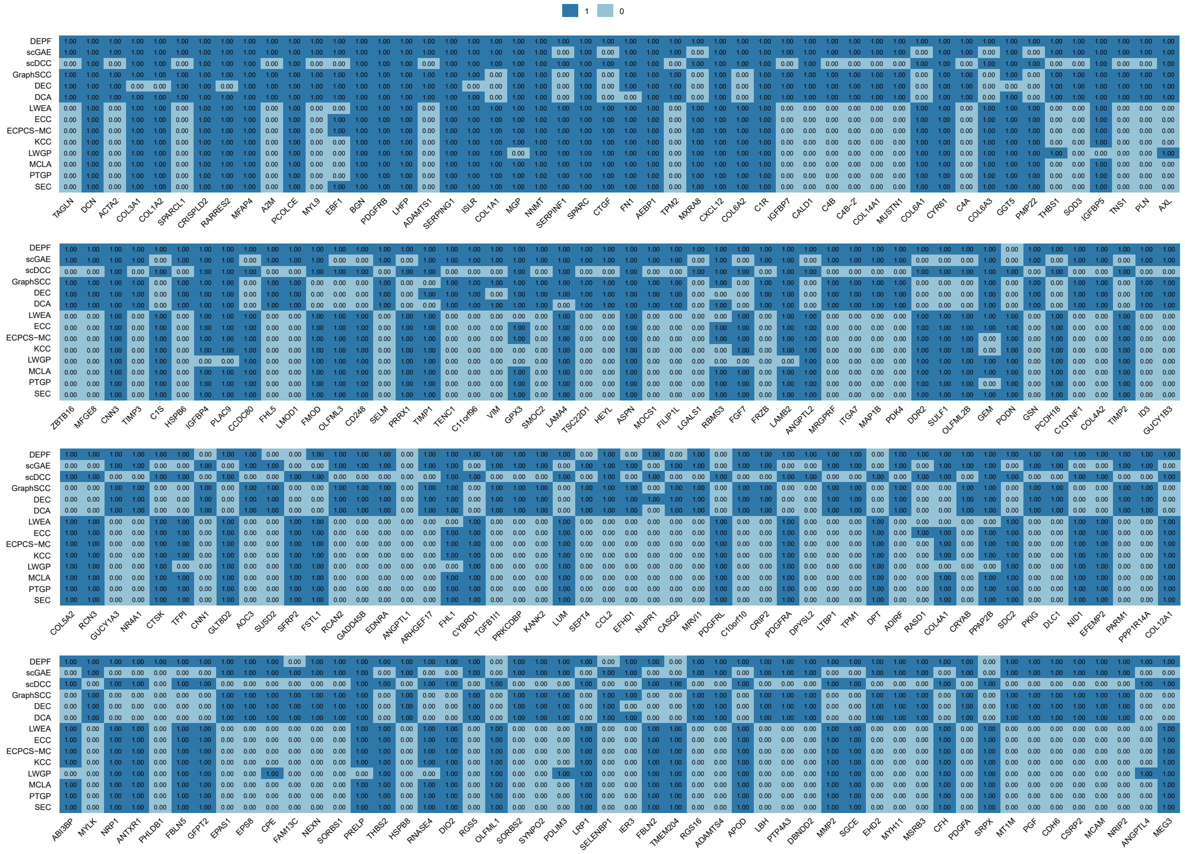

Figure S6: The intersection of the 200 differentially expressed genes from truth with the 200 differentially expressed genes from each of the 14 predictions. '1' indicates that the gene is both present in truth and predictions. '0' means the gene is not included in the prediction.

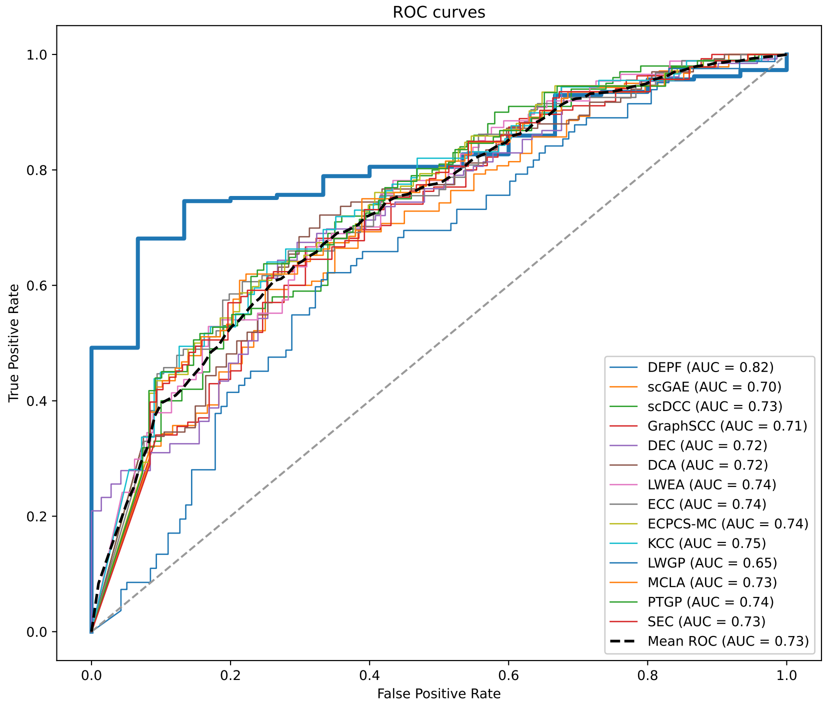

Figure S7: Accuracy measurements of differentially expressed genes in fibroblasts by different clustering methods.

expressed, whereas scGAE, scDCC, GraphSCC, DEC, DCA, LWEA, ECC, ECPCS-MC, KCC, LWGP, MCLA, PTGP, and SEC detected 140, 100, 135, 129, 133, 87, 94, 92, 89, 82, 92, 91, and 93, respectively. To further elaborate the performance of our algorithms, we also evaluated the area under the ROC curve (AUC) for each method and plotted the ROC curve, as summarized in Figure S7. In summary, DEPF (AUC = 0.82) is generally superior to other clustering methods.

8 Effect of different values of α (negative manage factor)

In our study, the negative manage factor α is designed to control the rate of change of the balancing factor ω, which is developed to ensure that fruit flies are balanced between exploration and exploitation in subsequent iterations. That is, fruit flies explore every corner of the space as much as possible in the early stage of the iteration to prevent themselves from getting into a local dilemma. The Figure S8 provides the relationship between the curves of α and ω. From this figure, we can observe that when the value of α rises, the rate of change of ω increases as well. To demonstrate the effect of different α values, we have conducted an experiment to evaluate the performance of the algorithm for different α values from the set {7, 9, 11, 13, 15, 17, 19}. The Figure S9 summarizes the clustering performance of different negative management coefficient α values on our 28 real scRNA-seq datasets in terms of the NMI metric. As depicted in this figure, We can clearly observe that DEPF obtained the best NMI values in 19 scRNA-seq datasets when α=13. On the Deng dataset, Usoskin dataset, Kolodziejczyk dataset, and Slyper dataset, the DEPF is unaffected by the change in α. Meanwhile, on the other 5 datasets, the results with different negative manage factors α are fairly similar. As a result, we determined that α should be 13 according to the DEPF results for the 28 real scRNA-seq datasets.

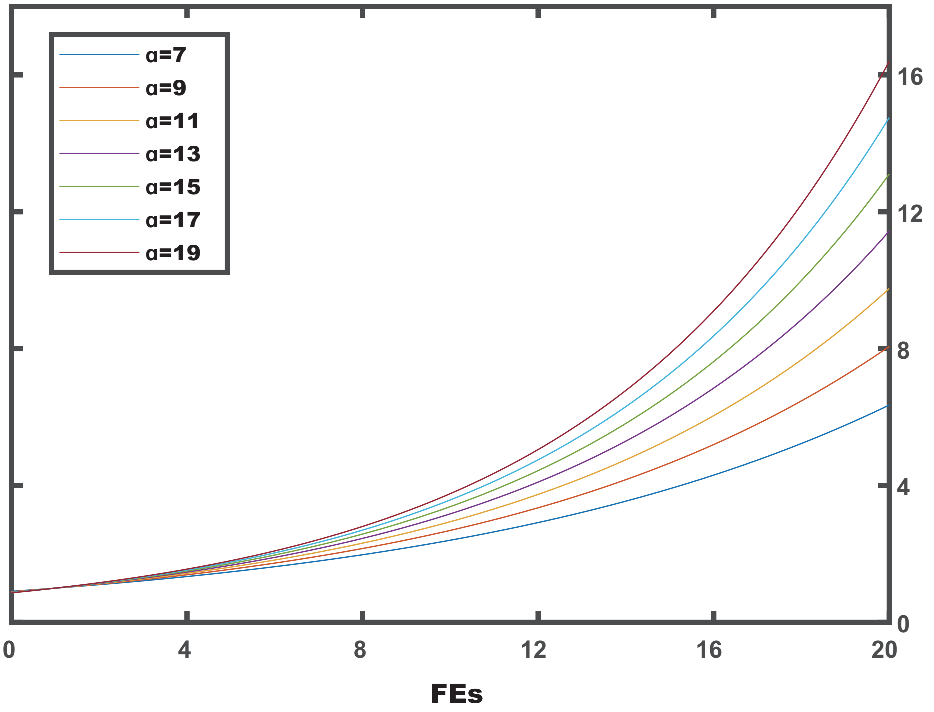

Figure S8: The curves of the balancing factor ω controlled by the the negative manage factor α.

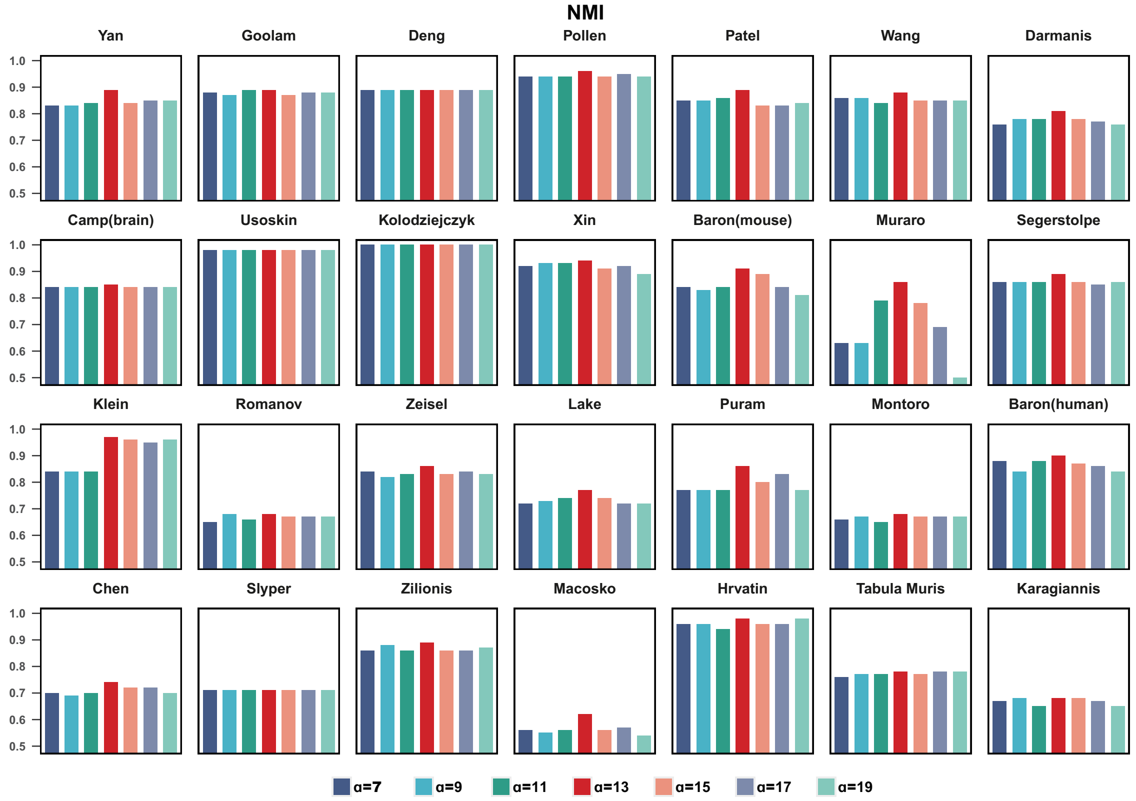

Figure S9: Clustering performance of DEPF with different α value measured by NMI on 28 real scRNA-seq datasets.

9 Implementation details

The Figure S10 provides line plots of the two objective functions, Cp and Dev changing with iteration. As depicted in this figure, we can observe that the convergence curves of Cp and Dev are consistent with the iterative process, i.e., better fruit fly generate a better solution, and a better solution produces a better generation. Specifically, both Cp and Dev converge gradually with the number of iterations of those 28 scRNA-seq datasets. Due to the different data distribution and complexity of those 28 scRNA-seq datasets, the convergence curves of Cp and Dev also exhibit diversity; for instance, on the Yan, Wang, Darmanis, Segerstolpe, Lake, and Chen datasets, the values of Cp and Dev are updated in each iteration. On the Goolam, Deng, Usoskin, Kolodziejczyk and Montoro datasets, the values of Cp and Dev do not change after the first population update is completed. On the Pollen, Camp(brain) and Hrvatin datasets, the rate of change of the curve is almost zero at approximately less than 10 iterations. Once this number is exceeded, Cp and Dev converge to a more optimal solution with a larger rate of change. There is also a case where the Cp and Dev curves on the Xin, Baron(mouse), Muraro, Puram,Tabula Muris, and Karagiannis datasets exhibit varying degrees of stagnation during convergence; for example, on the Muraro dataset, the Cp and Dev values are not updated for 1 to 15 iterations. The curves on the Baron(human), Slyper, Zilionis, and Macosko datasets show that Cp and Dev converge to the optimal solution by 10 ierations.

In addition, BOFOA is run 50 times independently on each scRNA-seq dataset to prevent ”lucky breaks”. The Figure S11 summarizes the boxplots of ARI and NMI for all results. In Figure S11, we can observe that the NMI and ARI results for Yan, Goolam, Deng, Camp(brain), Usoskin and Kolodziejczyk are very stable. There are some extreme values in the Pollen, Xin, Segerstolpe, Klein, Montoro, Slyper and Hrvatin datasets. The distribution of results for Wang, Romanov, Zeisel, Macosko, Tabula Muris, and kargiannis is relatively concentrated, whereas the distribution of results for Baron(mouse), Puram, Chen, Baron(human), and Zilionis is not ideal, but the average is still better than the other algorithms that were compared.

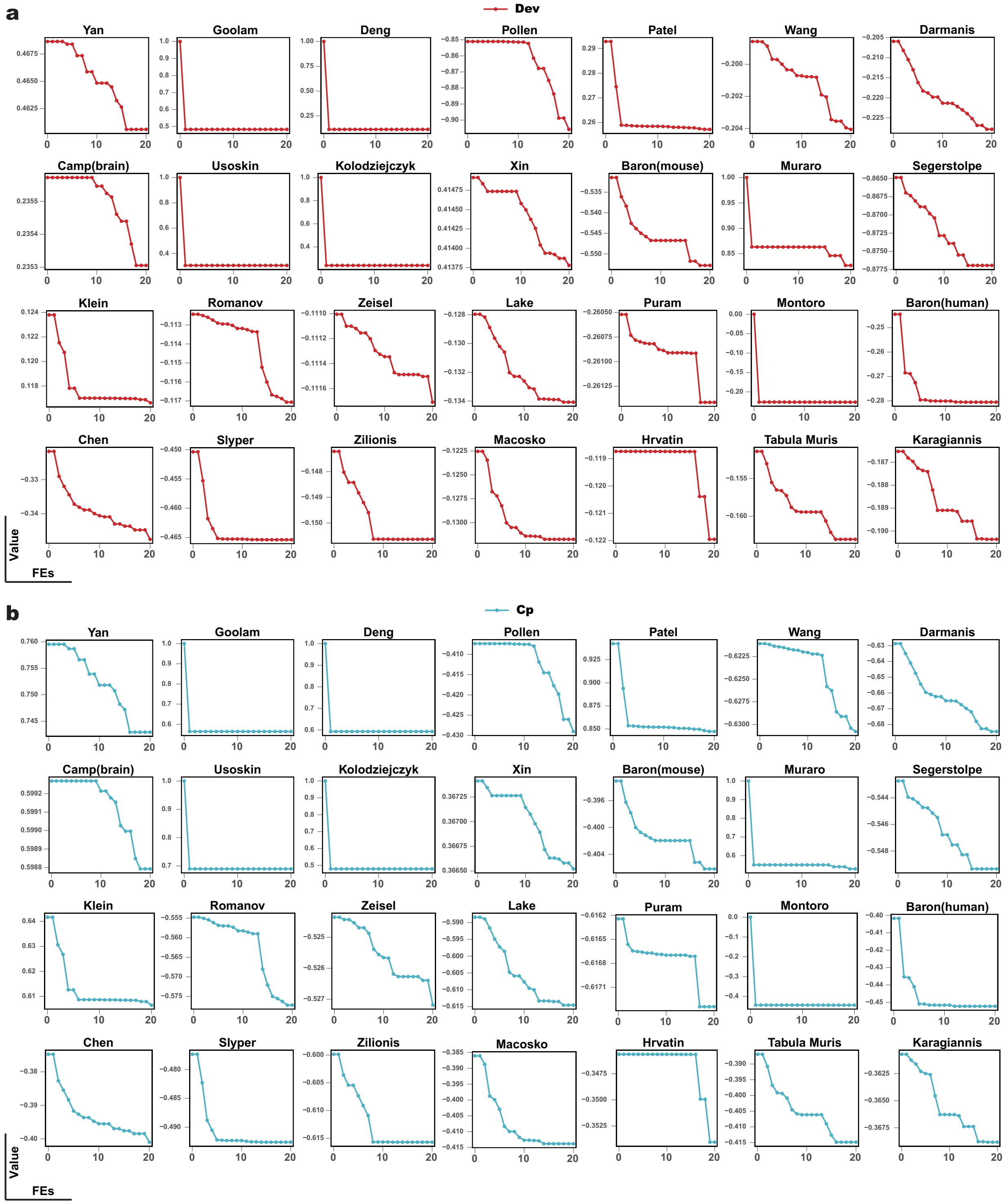

Figure S10: The line plots for two objective functions Cp and Dev changing with the iterations.

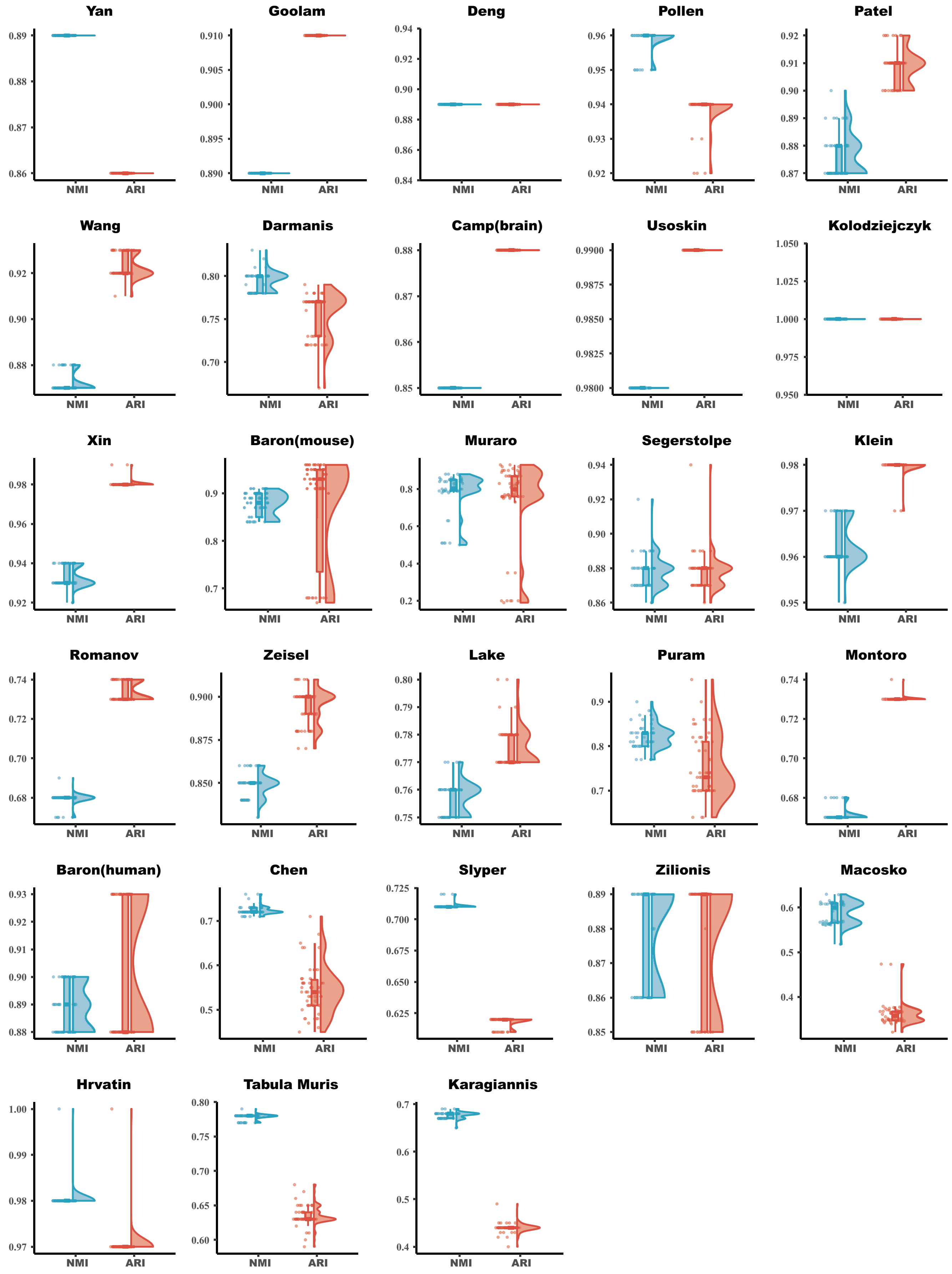

Figure S11: The boxplots of ARI and NMI for all 50 times of BOFOA running.

References

[1] D. Grün, M. J. Muraro, J.-C. Boisset, K. Wiebrands, A. Lyubimova, G. Dharmadhikari, M. van den Born, J. Van Es, E. Jansen, H. Clevers, et al., *Cell stem cell* **2016**, *19*, 2 266.

[2] M. J. Muraro, G. Dharmadhikari, D. Grün, N. Groen, T. Dielen, E. Jansen, L. Van Gurp, M. A. Engelse, F. Carlotti, E. J. De Koning, et al., *Cell systems* **2016**, *3*, 4 385.

[3] N. Lawlor, J. George, M. Bolisetty, R. Kursawe, L. Sun, V. Sivakamasundari, I. Kycia, P. Robson, M. L. Stitzel, *Genome research* **2017**, *27*, 2 208.

[4] Å. Segerstolpe, A. Palasantza, P. Eliasson, E.-M. Andersson, A.-C. Andréasson, X. Sun, S. Picelli, A. Sabirsh, M. Clausen, M. K. Bjursell, et al., *Cell metabolism* **2016**, *24*, 4 593.
